# Adjusting for switches to multiple treatments: Should switches be handled separately or combined?

**DOI:** 10.1177/09622802241300049

**Published:** 2025-01-17

**Authors:** Helen Bell Gorrod, Shahrul Mt-Isa, Jingyi Xuan, Kristel Vandormael, William Malbecq, Victoria Yorke-Edwards, Ian R White, Nicholas Latimer

**Affiliations:** 1 Sheffield Centre for Health and Related Research, School of Medicine and Population Health, University of Sheffield, Sheffield, South Yorkshire, UK; 2 Biostatistics and Research Decision Sciences (BARDS) Health Technology Assessment (HTA) Statistics, 2793 MSD, Zurich, Switzerland; 3 MRC Clinical Trials Unit at UCL, 4919 University College London, London, UK; 4 Biostatistics and Research Decision Sciences (BARDS) Health Technology Assessment (HTA) Statistics, 2793 MSD, Brussels, Belgium; 5 Statistique mathématique et Probabilités, 26659 Université libre de Bruxelles, Bruxelles, Belgium; 6 Centre for Advanced Research Computing, 4919 University College London, London, UK

**Keywords:** Treatment switching, multiple treatments, counterfactual, health technology assessment, oncology, overall survival, survival analysis, time-dependent confounding, time-to-event outcomes

## Abstract

Treatment switching is common in randomised controlled trials (RCTs). Participants may switch onto a variety of different treatments, all of which may have different treatment effects. Adjustment analyses that target hypothetical estimands – estimating outcomes that would have been observed in the absence of treatment switching – have focused primarily on a single type of switch. In this study, we assess the performance of applications of inverse probability of censoring weights (IPCW) and two-stage estimation (TSE) which adjust for multiple switches by either (i) adjusting for each type of switching separately (‘treatments separate’) or (ii) adjusting for switches combined without differentiating between switched-to treatments (‘treatments combined’). We simulate 48 scenarios in which RCT participants may switch to multiple treatments. Switch proportions, treatment effects, number of switched-to treatments and censoring proportions were varied. Method performance measures included mean percentage bias in restricted mean survival time and the frequency of model convergence. Similar levels of bias were produced by treatments combined and treatments separate in both TSE and IPCW applications. In the scenarios examined, there was no demonstrable advantage associated with adjusting for each type of switch separately, compared with adjusting for all switches together.

## 1 Introduction

Treatment switching in randomised controlled trials (RCTs) refers to a situation where trial participants switch onto a treatment that they were not randomised to.^1–8^ Participants may switch from the experimental treatment onto the control arm treatment, or may switch to another subsequent treatment.^7^ This becomes a problem for health care decision making if the switched-to treatment is not considered to be part of the standard treatment pathway. Typically, health technology assessment (HTA) agencies assess the effectiveness and cost-effectiveness of inserting a new treatment into the existing treatment pathway. Therefore, if treatment switches observed in an RCT are not consistent with the existing treatment pathway (for the control group), or are not consistent with the potential treatment pathway (for the experimental group), the RCT will not directly address the decision problem faced by the HTA agency. Instead, interest lies in a comparison between randomised groups of the outcomes that would have been observed in the absence of treatment switching, termed a hypothetical estimand.^9^ Statistical methods, here called adjustment methods, to estimate this quantity are available.^1–8^ However, research on adjustment methods has typically focused on adjusting for switches between randomised treatments, rather than scenarios where there is more than one treatment that patients could switch on to. In this study, we evaluate methods for adjusting for switches to multiple treatments, focusing on the first switch away from the treatment pathway of interest.

The most commonly used methods to adjust for treatment switching are the rank preserving structural failure time model (RPSFTM), inverse probability of censoring weights (IPCW) and two-stage estimation (TSE).^7^ These methods are described in National Institute for Health and Care Excellence Decision support Unit technical support document 16 and have been used in HTA around the world.^2,6,7^ Previous simulation studies have focused on the application of these methods in scenarios where trial participants switch from the control group onto the experimental treatment.^4,10,11^ Although Xu et al. consider the situation where patients in the control group of a trial switch treatments and receive two different levels of treatment effect,^12^ no previous study has thoroughly examined the performance of the most commonly used adjustment methods across a range of scenarios where there is more than one treatment that patients could switch on to.

The RPSFTM assumes a common treatment effect – it is generally assumed that patients switch from the control group onto the experimental treatment, and that the treatment effect received by switchers is the same as that received by patients originally randomised to the experimental group.^13^ If control group patients switch onto some other treatment that does not have the same effect as the experimental treatment, the common treatment effect assumption will not hold. Furthermore, if patients in the experimental group switch onto other treatments and adjustment is required, the RPSFTM is unlikely to be suitable. Extensions of the RPSFTM to allow the estimation of multiple treatment effects (and therefore for switches to multiple treatments) have been tested,^14,15^ but have failed due to the reliance of the method on identifying treatment effects solely based on the power of randomisation.

IPCW and TSE methods are more suited to situations where patients switch onto multiple treatments with differing effects, because they do not rely on a common treatment effect assumption. The IPCW makes no assumptions about the effects of the treatments being switched to, because switchers are simply censored from the analyses. The TSE requires that the effect of subsequent treatments can be estimated, but there is no restriction on what that effect (or effects) might be. There are various ways that IPCW and TSE can be applied in the context of switches to multiple treatments. Broadly, these approaches can be described as either (i) treatments combined or (ii) treatments separated. In a treatments combined approach, adjustments are made by grouping switches together regardless of the treatment switched to, whereas the treatments separated approach adjusts for each treatment switched to separately. Whilst in the interests of accuracy it may seem preferable to adjust for each type of switching separately, it is not clear that this approach is superior. Adjusting for each type of switching separately may be prone to error when switching numbers are small, and it may be adequate to adjust based on an ‘average’ effect of switching (for TSE) or based on the similarity of non-switchers to all switchers combined (for IPCW).

Our aims are (1) to describe different ways to handle switches to multiple treatments using IPCW and TSE, using the separate and combined approaches; (2) to assess the performance of these applications of IPCW and TSE using a simulation study in scenarios typical in oncology RCTs, with switching to multiple treatments; (3) to provide guidance on applying IPCW and TSE in these contexts based on our results.

## 2 Methods

### 2.1 Treatment switching adjustment methods

A summary of the IPCW and TSE applications is provided below. Further technical details are provided in Appendix 1.

#### 2.1.1 IPCW

The IPCW method involves censoring switchers at the time of switch, and weighting non-switchers according to their similarity in prognostic characteristics to switchers.^16,17^ Censoring switchers is likely to result in informative censoring, because switchers are likely to be prognostically different to non-switchers. By weighting non-switchers according to their prognostic similarity to switchers (that is, upweighting non-switchers who have similar prognostic characteristics to switchers), the selection bias induced by the informative censoring is removed. Weights (stabilised or unstabilised) are generated based on the baseline and time-dependent characteristics of patients using logistic models. A key assumption for the IPCW method is no unmeasured confounding, which requires that variables influencing the probability of switching and survival are included in the switching model used to derive weights. The positivity assumption is also imposed, requiring that no participants are certain to switch.

In the case of switching to multiple treatments, there are different ways that weights can be estimated. The aim is to use the weights to correct for the selection bias caused by censoring switchers. This could be achieved by modelling all types of switch with one model (combining the treatments switched-to), or by modelling each type of switch separately. We tested these two approaches, described in more detail below. We used stabilised weights, though applications with unstabilised weights were also tested. See Appendix 1 for more details on the denominator and numerator switching models, and Appendix 2 for results of unstablised analyses).

**IPCW-C: Application of IPCW with treatments combined**

IPCW-C involved combining the treatments switched-to, with no distinction made in the switching models between the switched-to treatments. Patients were censored at the time of switch and switching models were estimated using a binary logistic model with switch versus no switch as the dependent variable and confounders as explanatory variables. This approach assumes that the selection bias caused by censoring switchers can be corrected for adequately by combining all switchers and using one binary switching model. Weights were calculated using the probability of switching derived from the switching models. It is important to note that an IPCW analysis that combines switchers in this way is valid provided there are no unmeasured confounders and that the model is specified correctly – even if switchers to different treatments have consistently different prognostic characteristics. However, an expected limitation of this approach is that if the characteristics of patients switching to different treatments *are* very different, then specifying an appropriate model for the probability of switching may be difficult – the challenge here is a modelling problem, rather than a methodological problem.

**IPCW-S: Application of IPCW with treatments separate**

In IPCW-S, we distinguished between switching to different types of treatment. The switching model was estimated using a multinomial logistic model, where the dependent variable indicated zero to represent no switch, and *n* = 1, 2, … *N* to represent a treatment switch to treatment n. Weights were obtained using the probability of not switching extracted from the switching model. Because IPCW-S models each type of switch separately, in instances where switchers to different treatments have different prognostic characteristics, specifying accurate switching models may be more straightforward than under the IPCW-C approach. However, the multinomial logistic model may be expected to be more susceptible to convergence issues than a binary logistic model, and it is unclear whether estimating weights specific to each switching type will result in improved adjustment compared to estimating weights based on all switches combined.

#### 2.1.2 TSE

Both the simple TSE method^11^ and the more complex TSE that incorporates g-estimation were applied.^10^^,^^18^ All applications of TSE were applied with and without recensoring. The recensoring process is described in Appendix 1. Results of applications that did not include recensoring are presented in Appendix 2.

##### 2.1.2.1 Simple TSE

The simple TSE method, referred to as TSEsimp, requires that switching only occurs after a specific disease-related timepoint, such as disease progression, which is referred to as a secondary baseline. TSEsimp uses an accelerated failure time (AFT) regression model to calculate the treatment effect associated with switching (in the form of a time ratio), which is then used to derive counterfactual survival times that would have occurred in the absence of switching. Simple regression methods cannot deal with time-dependent confounding, and so TSEsimp requires that (i) switching occurs immediately at the secondary baseline time-point, or (ii) no time-dependent confounding occurs between the secondary baseline time-point and the time of switch, and (iii) the no unmeasured confounding assumption holds at the secondary baseline time-point, such that any prognostic differences between switchers and non-switchers can be controlled for. We tested two applications of TSEsimp to adjust for switches to multiple treatments:

**TSEsimp-C: Application of TSEsimp with treatments combined**

TSEsimp-C involved estimating the time ratio treatment effect associated with switching by combining all switches together. The resulting treatment effect represented an average of the effects of the switched-to treatments. To generate the counterfactual survival times, observed survival times for all switchers were adjusted using the same time-ratio treatment effect. The underlying assumption of the ‘combined’ approach when applying TSEsimp is that switching can be adequately adjusted for by combining all switchers and estimating an average treatment effect associated with switching, and then using this effect to generate counterfactual survival times for all switchers. As described above, the combined IPCW approach (IPCW-C) is valid provided there is no unmeasured confounding and correct model specification – the model can estimate appropriate weights for all patients, even if switchers to different treatments have different prognostic characteristics. For TSEsimp, the combined approach is more methodologically problematic: if the effects of different treatments that patients switch to are different, then adjusting using an average effect will not be accurate for individual patients. However, the ‘average’ counterfactual survival for a treatment group – which is often the estimand of interest – could still be accurate.

**TSEsimp-S: Application of TSEsimp with treatments separate**

In TSEsimp-S, one AFT model was used to estimate all separate time-ratio treatment effects for the different switched-to treatments, by specifying a model with indicator variables for each switch type. The treatment effect associated with treatment 1 was used to adjust the survival times for patients that switched to treatment 1, and the effect associated with treatment 2 was used to adjust the survival times for patients that switched to treatment 2, etc, for each of the *N* treatments switched onto. The benefit of estimating separate treatment effects is that differences in the effect of the switched-to treatments are allowed for, and therefore accurate adjustments can theoretically be made at both the individual and the treatment group level. However, a potential limitation is that due to small sample sizes, estimates of separate treatment effects may be more prone to error.

##### 2.1.2.2 TSE with g-estimation

TSE with g-estimation (referred to as TSEgest) extends TSEsimp by using a structural nested AFT model and g-estimation rather than simple regression to estimate the treatment effect associated with switching. This allows the method to deal with time-dependent confounding, and means that TSEgest does not require that no confounding occurs between the secondary baseline time-point and the time of switch – providing that confounders are measured during this period (i.e., the no unmeasured confounding assumption holds).^9^ In fact, TSEgest does not require that a secondary baseline exists at all, because any time-dependent confounding between the original study baseline and the time of switch can be adjusted for (again, providing confounders are measured). In this study, switching was only permitted post-progression, and therefore the structural nested AFT with g-estimation was applied to post-progression survival in the arm of the trial affected by switching. Therefore, TSEgest exactly matched TSEsimp, except a structural nested AFT model with g-estimation was used to estimate treatment effects, rather than a simple regression AFT model. We tested two applications of TSEgest:

**TSEgest-C: Application of TSEgest with treatments combined**

TSEgest-C combined all switchers and estimated an average treatment effect across all switched-to treatments, matching TSEsimp-C. This treatment effect was applied to adjust survival times for all switchers. The underlying assumption of the ‘combined’ TSEgest approach is the same as that of TSEsimp-C – that switching can be adequately adjusted for by combining all switchers and estimating an average treatment effect associated with switching, and then using this effect to generate counterfactual survival times for all switchers. Again, the expected limitation of this approach is that if the switched-to treatments have different effects, then adjusting using an average effect will not be accurate at the individual level – but may provide accurate counterfactual survival times at a treatment group level.

**TSEgest-S: Application of TSEgest with treatments separate**

The application of TSEsimp-S that estimated separate treatment effects for all switched-to treatments in one model cannot be applied using TSEgest, because it is only possible to extract one treatment effect from the structural nested AFT model. Instead, to estimate treatment effects for each switched-to treatment, separate AFT models with g-estimation were applied. The models compared post-progression survival in patients switching to treatment n, with non-switchers in the control arm. This was repeated for each switched-to treatment. Counterfactual survival times were estimated by adjusting survival times for patients switching to treatment n using the treatment effect associated with a switch to treatment n. As for TSEsimp-S, the benefit of estimating separate treatment effects is that differences in the effect of the switched-to treatments are allowed for, and therefore accurate adjustments can theoretically be made at both the individual and the treatment group level. However, due to small sample sizes, estimates of separate treatment effects may be more prone to error.

**ITT and ‘No Switching’ Analyses**

Alongside the switching adjustment methods, we conducted standard intention-to-treat (ITT) analyses and ‘No Switching’ analyses. It is standard practice to conduct ITT analyses of RCTs, and presenting these results provides context for the performance of the adjustment methods – demonstrating the bias associated with an ITT analysis in a context where it is desirable to adjust for treatment switches, and providing information on how this bias can be reduced by using adjustment methods. The ‘No Switching’ analyses represent the results of ITT analyses undertaken on each simulated dataset with no switching applied. Essentially, this represents the ‘truth’ for each simulated dataset within each scenario, and represents the best performance that an adjustment method could possibly achieve.

### 2.2 Simulation study

We describe the simulation study using the ADEMP structure recommended by Morris et al. (2019).^19^ Further technical details are provided in the Appendices.

#### 2.2.1 Aims

The simulation study was undertaken to test the performance of IPCW and TSE combined and separate approaches for adjusting for switches to multiple treatments. Forty-four scenarios were defined to represent plausible oncology RCTs, with switching permitted in the control group after disease progression. The methods aim to estimate survival outcomes that would have been observed in the absence of treatment switching. Four additional scenarios were performed to test the sensitivity of the results to less clinically plausible scenarios.

#### 2.2.2 Data generating mechanisms

The data generating process is described in detail in Appendix 3. The steps of the process are summarised as follows:

1. **Underlying survival times** – Initial survival times were generated for an RCT with 1:1 randomisation (sample size 500 or 1000) using the survsim command in Stata,^20^ with a 2-component mixture Weibull survival function with shape and scale parameters that remained the same across scenarios. A binary prognosis variable (good/bad) was also created. Time was divided into 21-day periods from randomisation to death, where a 21-day period is representative of the typical cycle separating two clinic visits in oncology.
2. **Time to disease progression** – Overall survival time was multiplied by a random number selected from a beta distribution with scale and shape parameters (5,10) to obtain time to disease progression.
3. **Time-dependent confounding** – A time-dependent metastatic disease variable, *M*, was created. Metastatic disease (*M = 1*) could occur at the four visits following disease progression, with probability depending on the treatment received and baseline prognosis. If *M = 1*, the remaining survival time was reduced and the probability of switching was altered.
4. **Time of switching** – For simplicity, switching was only permitted in the control group. Switching could happen at the time of disease progression or at any of the subsequent five visits following disease progression. A maximum of one switch was modelled for each participant.
5. **Nature of switching** – The probability of switching was a function of baseline prognosis group and whether or not *M* had occurred. Each switcher was allocated a treatment to which they switch. The treatment switched-to depended on baseline prognosis and was allocated using a binomial random draw. The allocation was designed to allow the prognostic characteristics of patients that switched to treatment 1 to differ, on average, from the prognostic characteristics of patients that switched to treatment 2. In scenarios 1–40, patients in the control arm could switch to one of two treatments. In scenarios 41–44, patients in the control arm could switch to one of five treatment options.
6. **Effect of switching on metastatic event and survival times** – For switchers that did not have metastatic disease prior to switching, the occurrence of *M* was recalculated on the basis that the probability of metastatic disease occurring at time *t* would be reduced following a switch to a beneficial treatment. Survival times were recalculated according to whether or not *M* occurred after switching, and were further adjusted according to the effect of the treatment switched to. *M* was recalculated for any additional survival time lived.
7. **Apply censoring** – Censoring was applied at 730 days (20% censoring) in scenarios 1–16 and 41–44, 500 days (40% censoring) in scenarios 17–32 and 300 days (70% censoring) in scenarios 33–40.

#### 2.2.3 Parameters

**Figure 1.**
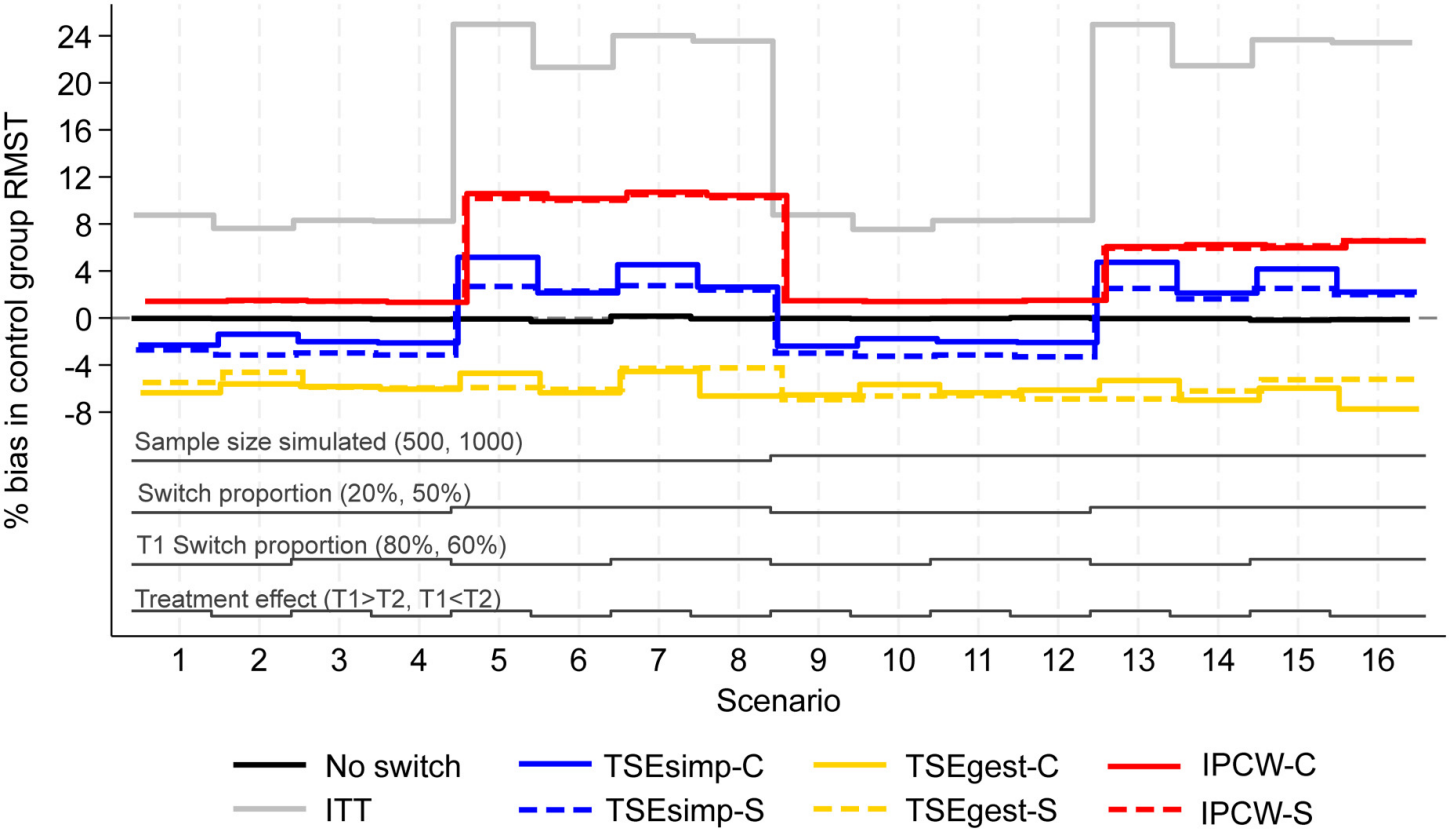
Percentage bias in control group RMST, scenarios 1–16 (censoring approximately 20%).

Parameters relating to the underlying survival model, baseline prognosis, and metastatic event remained consistent across all scenarios, and were chosen to represent realistic survival time distributions similar to those used in previous simulation studies.^10^ Using a mixture Weibull model allowed us to simulate a hazard function with a turning point, which is common in oncology.^21,22^ Typical survival curves produced by simulations within scenario 1 are presented in Figure 1, Appendix 4. Parameters relating to the proportions of switchers, treatment effects of the switched-to treatments, the proportion of censored events, and the number of switched-to treatments were varied, resulting in 48 scenarios. Appendix 5 lists the scenarios and associated model parameters, including switch proportions and treatment effect sizes associated with each scenario. The time ratio treatment effects of 1.5 and 1.2, or 1.2 and 1.7 in scenarios 1–40, were specifically selected to present upper and lower bounds of realistic differences in expected treatment effect, with the expectation that clinicians would not choose to switch patients onto a treatment when a far more effective alternative treatment was available for patients to switch onto. Scenarios 41–44 allow for switches to five different treatments, with time ratios ranging from 1.2 to 1.8. Scenarios 45–48 tested the sensitivity of the results to a larger difference in treatment effects, with time ratios of 1.1 and 2.2 for treatments 1 and 2 respectively.

#### 2.2.4 Estimand

Our estimand was the restricted mean survival time (RMST) that would have been observed in the control group in the absence of treatment switching. This matches the estimand used in several previous simulation studies undertaken on switching adjustment methods.^4,10,11,23,24^ Although the difference between treatment arms is generally the parameter of interest in RCTs, we focus on control group RMST because we only simulated switching in the control group and our aim was to investigate the performance of adjustment methods in estimating survival times that would have been observed in the absence of switching.

Because the survival function generated by our data generating mechanism was analytically intractable, we estimated the true RMST value for each scenario by simulating a large number of patients (100,000) without including treatment switching, and then calculating the area under the Kaplan-Meier survivor function up to the maximum censoring time for each scenario.

No switch represents an analysis on the simulated data before the switch was incorporated, which relates to steps 1 and 2 only in the data generating process described in Section 2.2.2. Other analyses were performed on simulated data with switch incorporated (steps 1–5 of the data generating process described in Section 2.2.2). ITT represents the intention-to treat-analysis (no adjustment made for switching), TSEsimp, TSEgest and IPCW represent the application of the treatment switching adjustment methods, -C suffix represents the combined adjustment strategy and -S suffix represents the separate adjustment strategy.

#### 2.2.5 Adjustment methods compared

We investigated the adjustment methods described in Section 2.1. To obtain estimates of control group RMST for each adjustment method, we fitted flexible parametric models to the counterfactual data (for TSEsimp and TSEgest) and to the weighted data (for IPCW) and derived the survivor function (extrapolated if necessary) up to the maximum censoring time for each scenario. Using parametric models was necessary because applications of TSEsimp and TSEgest that incorporate recensoring can lead to final follow-up times in adjusted data-sets being less than the maximum unadjusted censoring times. In such instances, using parametric models allowed extrapolation to a common survival time, ensuring that all RMST comparisons compared ‘like with like’. Using flexible parametric models is recommended when extrapolating complex hazard functions.^21^ We used the Stata command stpm2 to fit the flexible parametric models.^25^

#### 2.2.6 Performance measures

Results from each method were captured for 1000 iterations of each scenario. To assess the performance of the methods, the results were compared against truths for each scenario. Performance of the methods was assessed using the following measures: (i) convergence of the method, (ii) percentage bias, (iii) empirical standard error and (iv) root mean squared error. A Monte Carlo standard error (MCSE) was calculated alongside each of these performance measures, to assess the level of uncertainty surrounding these measures resulting from the selected number of simulated iterations.

**Figure 2.**
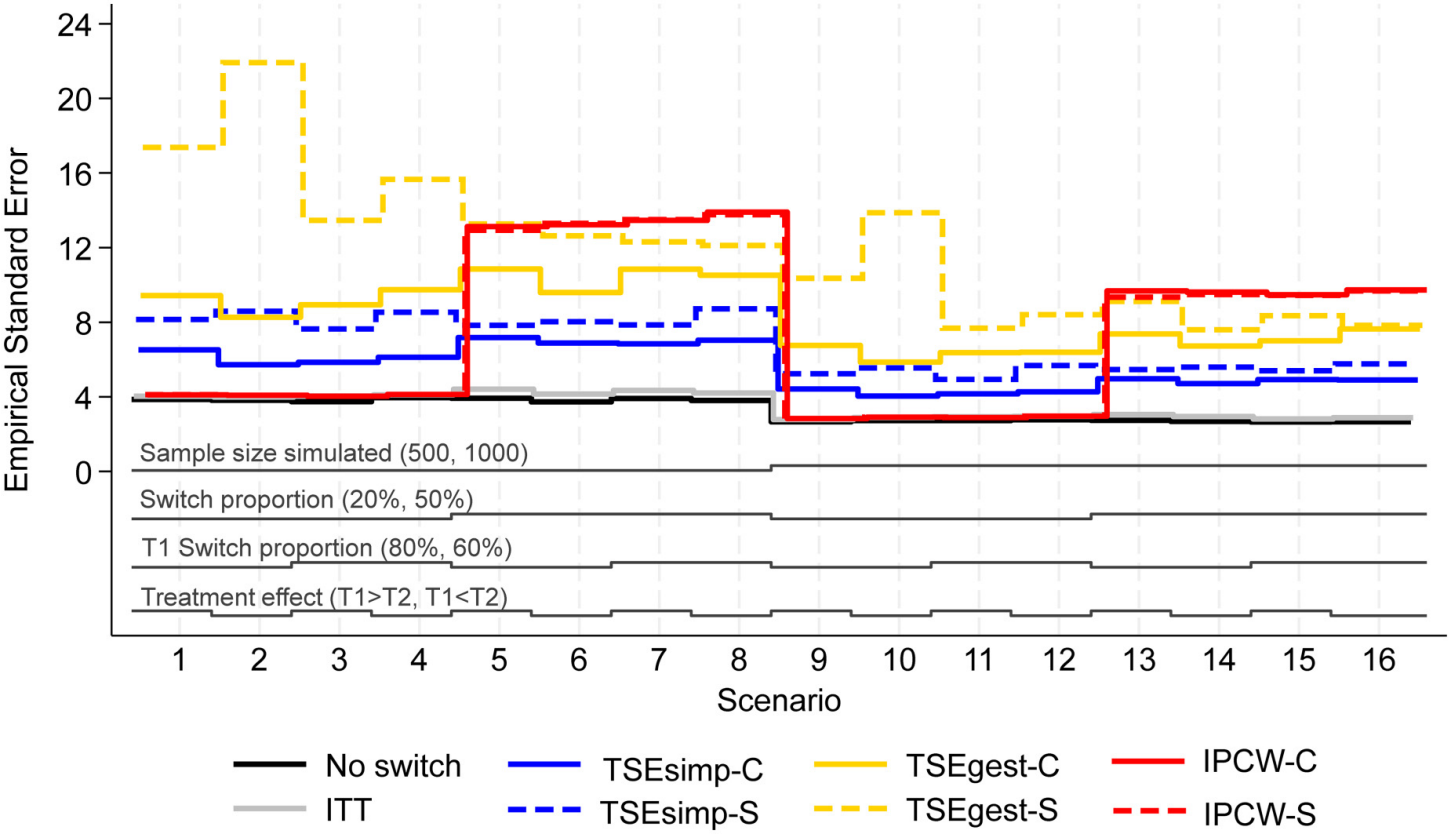
Empirical standard error, scenarios 1–16 (censoring approximately 20%).

**Figure 3.**
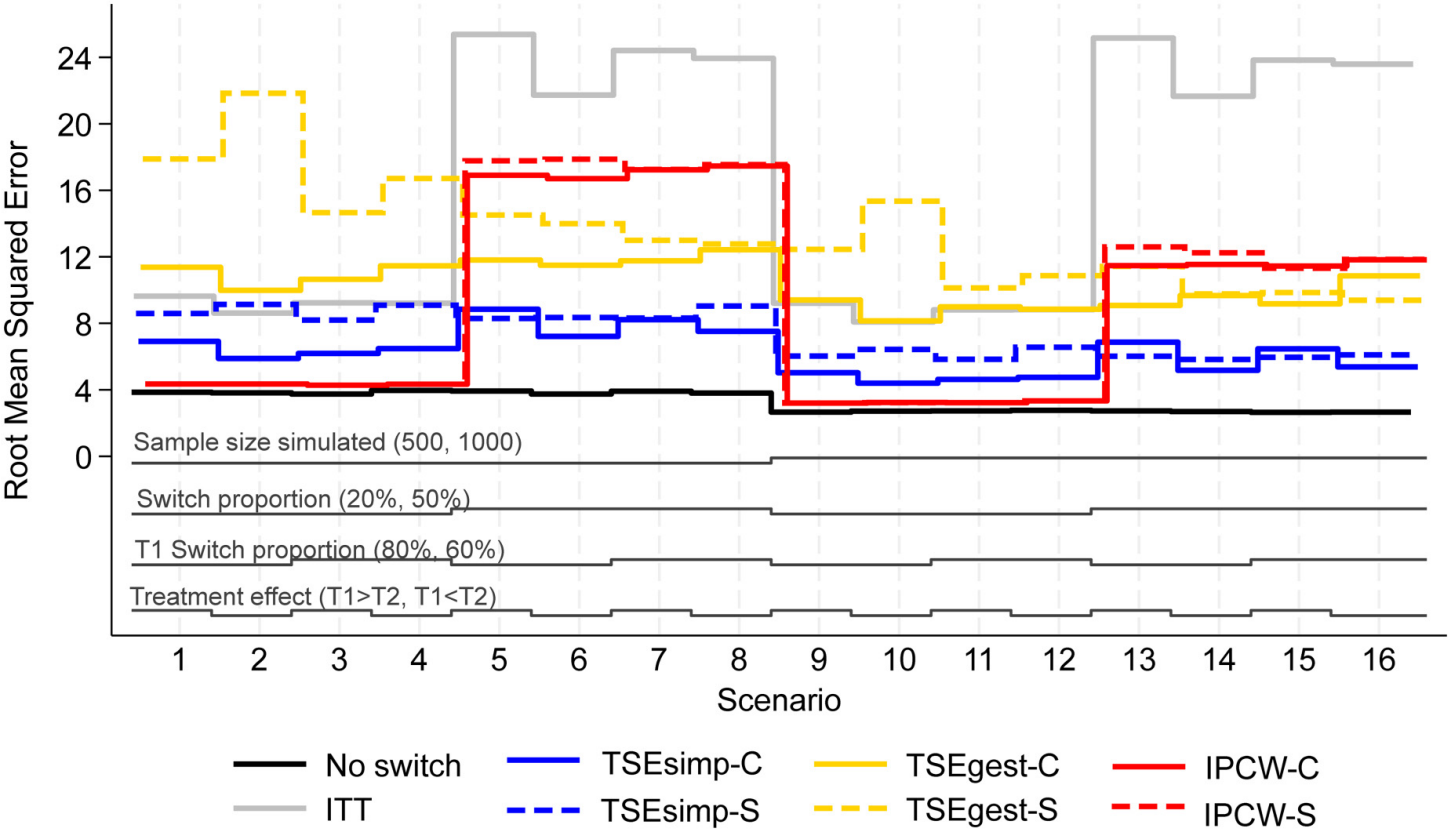
Root mean squared error, scenarios 1–16 (censoring approximately 20%).

**Figure 4.**
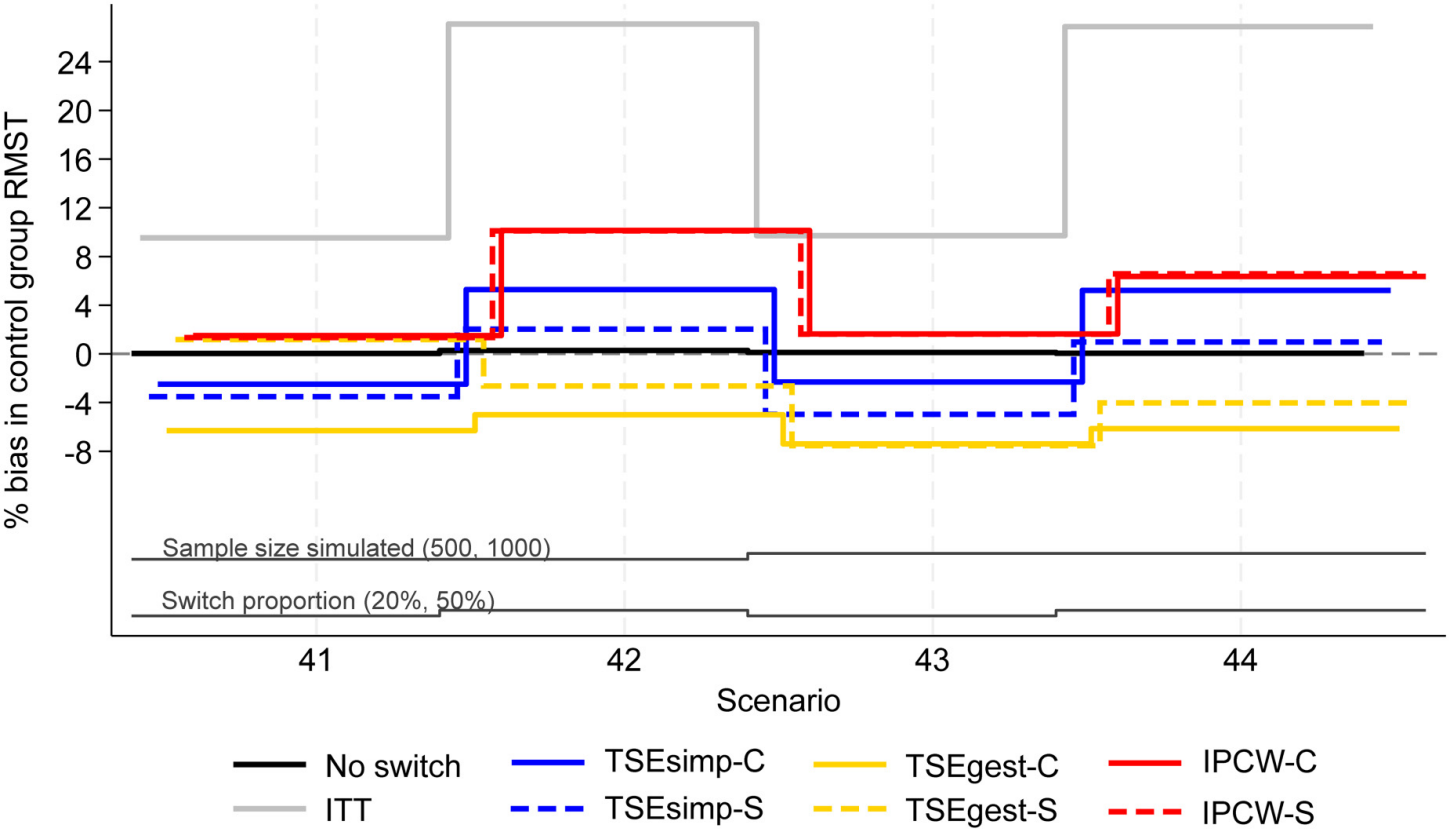
Percentage bias in control group RMST, scenarios 41–44 (censoring approximately 20%).

**Figure 5.**
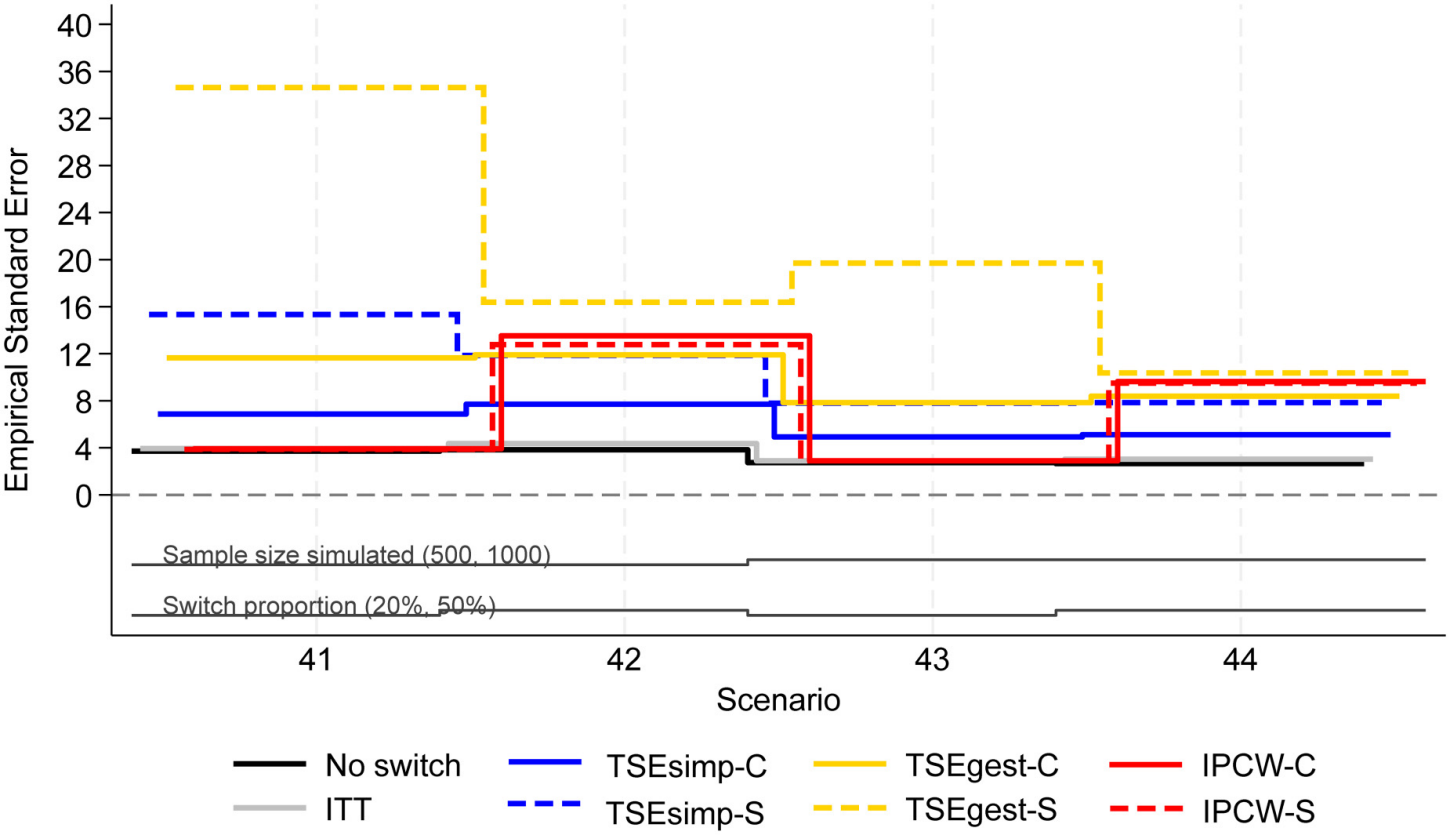
Empirical standard error, scenarios 41–44 (censoring approximately 20%).

**Figure 6.**
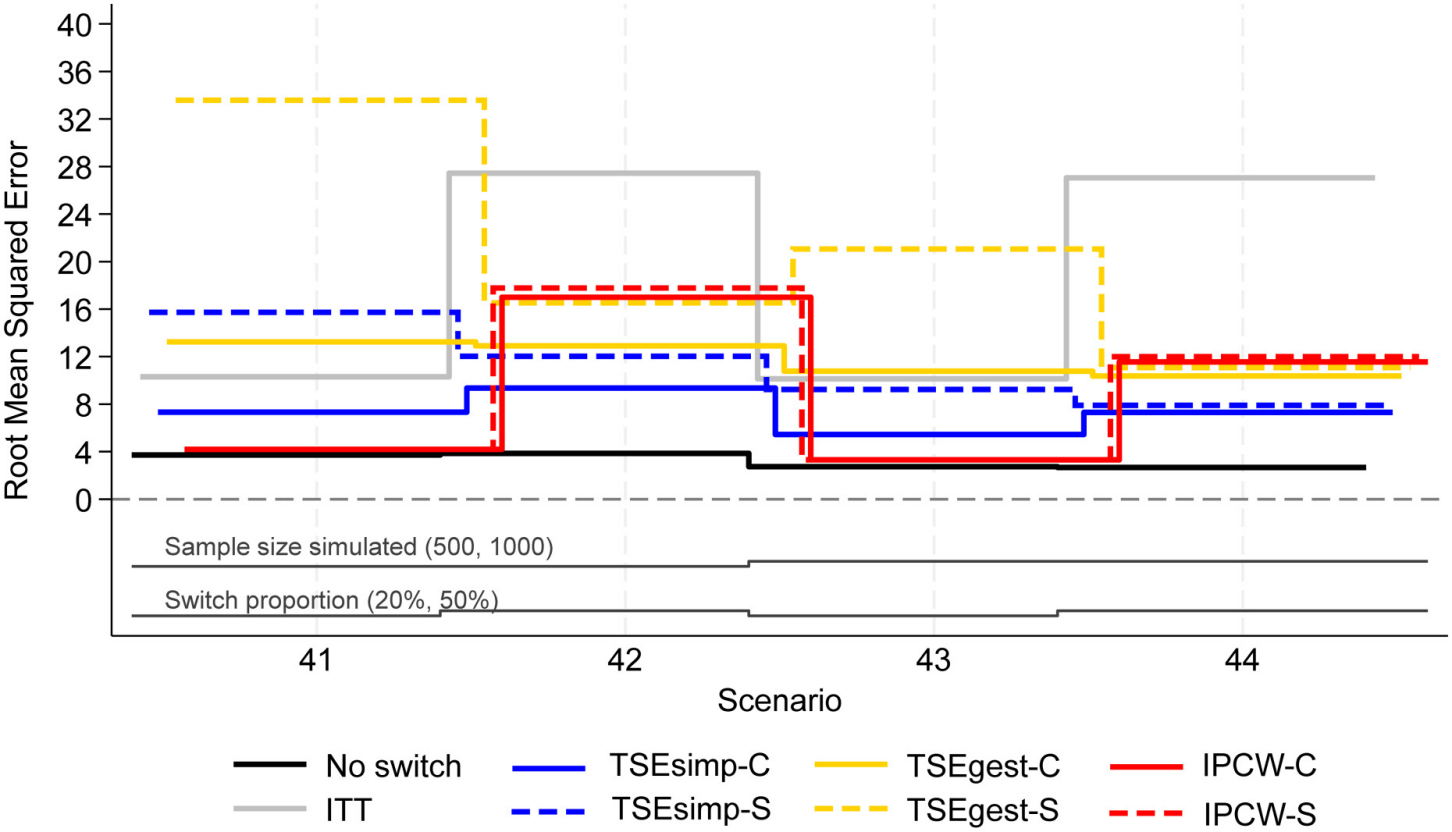
Root mean squared error, scenarios 41–44 (censoring approximately 20%).

## 3 Results

Results are firstly presented for scenarios allowing switching to two treatments, followed by scenarios that permitted switches to five treatments. Figures 1 to 6 depict results for IPCW with stabilised weights, TSEsimp with recensoring, and TSEgest with recensoring, along with the ITT and ‘No Switching’ results for comparison in scenarios 1–16 and 41–44. Further results, including scenarios 17–40 with different censoring proportions in the simulated data, and applications of IPCW with unstabilised weights, TSEsimp and TSEgest without recensoring can be found in Appendix 2.

**Switching to two treatments**

In scenarios 1–40, patients in the control group could switch to one of two treatments (treatment 1 or treatment 2) after disease progression. Scenarios 1–16, presented in Figures 1–3, had the following characteristics: a sample size of 500 or 1000 patients; switching proportions of 20% or 50%; proportions of switchers that switched to treatment 1 (T1) of 80% or 60%; relative treatment effects of T1 and treatment 2 (T2) such that the treatment effect of T1 was either greater than or less than T2; censoring time set at 730 days (approximately 20% of survival events censored). Scenarios 17–32 (Appendix 2) repeated scenarios 1–16 but with censoring set at 500 days (approximately 40% of survival events censored), and scenarios 33–40 repeated scenarios 9–16 (those with a sample size of 1000) with censoring set at 300 days (approximately 70% of survival events censored).

Across these scenarios, the comparison of primary interest showed very little difference between analyses that took a combined or separate approach to adjusting for treatment switching. Figure 1 depicts the percentage bias in control group RMST in scenarios 1–16. The ‘No Switching’ method produced close to zero bias, as expected. The ITT analysis produced much higher bias with higher proportions of switching. Each of the treatment switching adjustment methods produced lower bias than the ITT analysis in each scenario.

IPCW-C and IPCW-S resulted in almost identical control group RMST percentage bias in scenarios 1–16. Both IPCW approaches produced lower bias in scenarios with 20% switching, than in scenarios with 50% switching. In scenarios with 20% switching (scenarios 1–4 and 9–12), both applications of IPCW had around 2% bias. A high percentage bias (above 10%) was produced by applications of IPCW in scenarios 5–8, with 50% switching. This was slightly reduced in scenarios 13–16, when the sample size of each simulated dataset was increased from 500 to 1,000, but remained above 6%. IPCW applications that used unstabilised weights (as opposed to the applications that used stabilised weights presented in Figure 1), produced similarly low bias in scenarios with 20% switching, but produced higher levels of bias than the stabilised version in scenarios with 50% switching (see Appendix 2).

The two applications of TSEsimp resulted in similar levels of percentage bias in scenarios 1–16 (Figure 1). The bias produced by TSEsimp-C tended to be slightly more sensitive to changes in the treatment effect parameters than TSEsimp-S, particularly in scenarios with 50% switching. In addition, TSEsimp-S was slightly more biased than TSEsimp-C in scenarios 5 and 7 where the difference between the treatment effect of treatments 1 and 2 was larger. The MCSE on the bias in scenarios 5 and 7 ranged from 0.217 to 0.248, suggesting that the difference in bias between TSEsimp-S and TSEsimp-C was not explainable by the MCSE. In general, both applications resulted in low levels of bias. In scenarios with 20% switching, TSEsimp applications produced levels of bias of approximately +/−2% (similar to those associated with IPCW), increasing to 2–4% in scenarios with 50% switching (appreciably lower than IPCW). Applications of TSEsimp that did not incorporate recensoring led to an upward shift in estimated RMST (see Appendix 2). In scenarios where TSEsimp with recensoring led to negative bias (scenarios 1–4 and 9–12), excluding recensoring resulted in smaller bias. In contrast, in scenarios where TSEsimp with recensoring led to positive bias (scenarios 5–8 and 13–16), excluding recensoring led to increased bias.

Both applications of TSEgest (with recensoring) consistently produced between −8 and −4% bias in scenarios 1–16 (Figure 1). TSEgest was much less affected by the switching proportion than IPCW, but in scenarios with 20% switching the IPCW applications produced lower bias. Of note, applications of TSEgest that did not incorporate recensoring produced similarly consistent results, but, as for TSEsimp, excluding recensoring led to an upward shift in estimated RMST, resulting in a reduction in bias across all scenarios for TSEgest applications (percentage bias reduced to between 0 and 3%, results provided in Appendix 2).

Empirical standard error (EmpSE) (Figure 2) and root mean squared error (RMSE) (Figure 3) show closely matched results for the combined and separate applications of IPCW. In contrast, EmpSE and RMSE for the combined applications of TSEsimp and TSEgest were lower than for the separate applications of the methods. The TSEgest approaches consistently produced higher EmpSE and RMSE than their TSEsimp counterparts, which is expected due to the greater variability resulting from the g-estimation process. The IPCW approaches showed low EmpSE and RMSE in scenarios where bias in RMST was low, and high EmpSE and RMSE in scenarios where bias in RMST was high. This is likely to be due to IPCW generally resulting in more bias when the distribution of estimated weights was wide, and it is also in these scenarios where IPCW is prone to error and wider variability.

Convergence rates by scenario and method are provided in Appendix 6 Table 1. Convergence was achieved in 97–100% of iterations across all scenarios for IPCW-C. For IPCW-S, convergence was achieved in 71–100% of iterations across scenarios, with convergence rates at their lowest in scenarios 5–8, where 50% of the control group switched treatment. In the iterations where convergence failed, this typically happened in the denominator switch model. All TSEsimp applications converged in 100% of iterations across all scenarios and all TSEgest applications converged in 99.9–100% of iterations across all scenarios. Our results remain valid when IPCW-C and IPCW-S were compared for the iterations of the simulation where IPCW-S converged (see Appendix 7).

Scenarios 17–32 involved censoring at 500 days and scenarios 33–40 involved censoring at 300 days (see Tables 17–40 in Appendix 2). In these scenarios, each of the adjustment methods resulted in similar levels of bias, EmpSE and RMSE to those described for scenarios 1–16, with the later censoring time of 730 days.

Additional analyses presented in Appendix 8 were performed to test the sensitivity of the results to a larger difference between the effect of treatment 1 and treatment 2, with time ratios of 1.1 for treatment 1 and 2.2 for treatment 2. Even with a larger difference between treatment effects, the combined and separate approaches still produced similar results for each method (IPCW, TSEsimp and TSEgest), and patterns of results for each method were similar to those produced from scenarios 1–40.

**Switching to five alternative treatments**

Scenarios 41–44 allowed patients in the control group to switch to one of five different treatments after disease progression. Results followed a similar pattern to the two-treatment results, but with lower levels of model convergence.

In scenarios 41–44, both IPCW applications produced low bias (<2%) in scenarios with 20% switching, and high bias (6–10%) in scenarios with 50% switching (Figure 4). IPCW-C resulted in almost identical levels of bias, empirical standard error, and RMSE, to IPCW-S. However, there were substantial differences in the convergence of the IPCW applications. In scenarios 42 and 44 with 50% switching, the multinomial logistic model used for IPCW-S only converged in 21% and 53% of iterations respectively, and therefore the results presented for IPCW-S in Figures 4 to 6 only represent 211 iterations of the simulation in scenario 42 and 531 iterations in scenario 44 (see Appendix 6 for further details). In contrast, IPCW-C had far fewer convergence issues, achieving convergence in 97–100% of iterations in scenarios 41–44. Applications of IPCW with unstabilised weights again produced similar levels of bias to those with stabilised weights, with the exception that unstabilised applications were susceptible to higher levels of bias in scenarios with 50% switching.

The two applications of TSEsimp resulted in similar levels of bias in scenarios 41–44. TSEsimp-S resulted in marginally lower bias than TSEsimp-C in scenarios with 50% switching, whereas the opposite was true in scenarios with 20% switching (Figure 4). The MCSE on the bias in scenarios 42 and 44 ranged from 0.162 to 0.375, where the MCSE was around 0.1 higher for TSEsimp-S than TSEsimp-C. Bias associated with TSEsimp was consistently low, ranging between −4% and 5%, in these scenarios. All applications of TSEsimp converged in 100% of iterations in scenarios 41–44. Empirical standard error and RMSE was generally higher for TSEsimp-S than TSEsimp-C in these scenarios.

The two applications of TSEgest also resulted in similar levels of bias in scenarios 41–44, with the exception of scenario 41, in which TSEgest-C resulted in bias of approximately −6%, whereas TSEgest-S resulted in bias of approximately 1% (Figure 4). However, empirical standard error and RMSE was much higher for TSEgest-S than TSEgest-C in this scenario, and this was also the case (to a slightly lesser extent) in scenarios 42–44 (Figure 5). Excluding TSEgest-S in Scenario 41, the bias associated with TSEgest applications was consistent across scenarios 41–44, ranging between −8% and −4%. Convergence remained high – 99–100% across all simulations for TSEgest-C, and 93–100% for TSEgest-S. Applications of TSEgest that did not include recensoring resulted in very low levels of bias (0–2%) in scenarios 41–44 for TSEgest-C, but were much higher (8–20%) for TSEgest-S, indicating that the application of TSEgest without recensoring that separated switchers was much more prone to error when adjusting for five types of switching (see Appendix 2).

## 4 Discussion

This simulation study investigates applications of treatment switching adjustment methods when it is necessary to adjust for switches to more than one treatment. We explored whether it was important to adjust for each type of switch separately, or whether it was sufficient to combine all switchers before adjusting. The results of the simulation study indicate that there is no advantage associated with using separate models to capture the effects of switching to different treatments.

Our study showed that IPCW applications that dealt with switchers combined or separately resulted in almost identical levels of bias and standard errors. IPCW convergence rates were lower when participants who switched to different treatments were dealt with separately, especially when participants could switch to one of five (as opposed to one of two) alternative treatments. This is because the multinomial logistic switching models used in the ‘switchers separate’ applications of the IPCW converged less frequently than the binary logistic switching models used in the ‘switchers combined’ applications. An alternative approach for dealing with switchers separately was also applied, which involved obtaining weights from *n* binary logistic models (as described in Appendix 9). Again, levels of bias were similar and fewer iterations converged than for IPCW-C. The IPCW findings demonstrate that even if prognostic characteristics are different for patients who switch to different subsequent treatments, it is not necessary to fit switching models separately for each type of switching. It is sufficient to group switchers – a correctly specified switching model will still be able to identify and upweight non-switchers who have similar characteristics to switchers.

There was also no advantage to adjusting for each type of switching separately when using TSE methods – it was sufficient to combine switchers and estimate an average treatment effect associated with switching. This indicates that estimating one treatment effect across all switchers and using that to derive counterfactual survival times for all switchers is essentially equivalent to estimating separate treatment effects for each type of switching and using those effects to derive counterfactual survival times in a more granular manner. Although the two techniques will result in different counterfactual survival times estimated for individuals, the impact on adjusted survival times for the treatment group as a whole will be similar. As for the IPCW, but to a lesser extent, the application of TSEgest that separately adjusted for different types of switching resulted in more convergence issues, and also more variable results. Therefore, again, our findings suggest that it is appropriate and sufficient to combine switchers when using TSE to adjust for multiple types of switching, rather than adjusting for each switch type separately.

IPCW performed well in scenarios with 20% switching, outperforming the other adjustment methods. However, in scenarios with 50% switching, IPCW was notably worse – especially when unstabilised weights were used, and when switches were adjusted for separately. TSEsimp and TSEgest were shown to perform better than IPCW in scenarios with high levels of switching, which is in line with previous studies. TSEgest may be expected to perform better than TSEsimp, due to its ability to deal with time-dependent confounding, yet this was not always the case in this study.

The poor performance of IPCW in scenarios with high proportions of switchers occurred due to there being fewer non-switchers available to upweight in these scenarios. This resulted in large weights applied to few patient-time points. Typically, when an application of IPCW produces high weights such as these the analysis is known to be prone to error. This has been shown in previous studies and in these circumstances IPCW should not be relied upon.^10,11,23^

TSEgest performed consistently across scenarios in our study, and was less sensitive to changes in the proportion of switchers than the other adjustment methods. This reflects the findings in Latimer et al.^10^ It is important to note that although TSEgest performed reasonably well in our study, an appreciable level of bias (4–8%) remained. In a previous study that used a similar data generating mechanism to that used in the present study, it was noted that some level of bias associated with TSEgest might be expected due to the treatment effect heterogeneity simulated (because switchers who had not experienced metastatic disease at the time of switch experienced a dual treatment effect through an extended survival time and a reduced probability of developing metastatic disease, whereas those who had experienced metastatic disease at the time of switch only received the extended survival time benefit).^10^ However, this residual bias was lower in the previous study, and it is likely that some of the bias observed in our study is due to recensoring. Previous research has found that recensoring can lead to bias, particularly when substantial amounts of important follow-up time are lost, and when there are turning points in hazard functions or treatment effects over time – which was the case in our study.^24^ Although we have not focused on recensoring in this paper because investigating this was not our purpose, our study provides further evidence on the sometimes undesirable impacts of recensoring, and indeed, applications of TSEgest that did not incorporate recensoring generally led to very low levels of bias.

Based on previous research,^10^ we would have expected TSEsimp to be outperformed by TSEgest in our simulation study, because we simulated scenarios with time-dependent confounding, which can be dealt with using TSEgest but not with TSEsimp. However, TSEsimp performed consistently well across all scenarios, often producing less bias than TSEgest and performing better than IPCW in scenarios with large switching proportions. We believe that the reason TSEsimp appeared to outperform TSEgest in this study is due to recensoring. As noted for TSEgest, in this study recensoring appears to have caused negative bias in RMST estimates. In the previous study that investigated TSEsimp and TSEgest,^10^ TSEsimp consistently resulted in positive bias in RMST estimates, particularly in scenarios that did not involve censoring. Therefore, we believe that in our scenarios, the negative bias caused by recensoring may have counteracted positive bias associated with TSEsimp, such that net bias is low. Hence, we would not conclude that TSEsimp is a better performing adjustment method than TSEgest in scenarios where time-dependent confounding is present, but rather that both methods performed well and the impact of recensoring is important to consider on a case-by-case basis.

Our study has limitations. As with all simulation studies, a finite set of scenarios can never capture the infinite range of situations that may occur in reality. However, we have explored a number of clinically plausible scenarios that cover a range of situations, and additionally tested the sensitivity of our results in less clinically plausible scenarios. Related to this, we only simulated switching to occur in the control arm of the simulated RCTs. In practice, switching to subsequent treatments often happens in both arms of an RCT. We chose to simplify our simulation study in this way because the application of the adjustment methods would be the same in whichever trial arm that switching took place – if a method is able to adjust for switching accurately in the control arm of a trial, it will also be able to adjust for switching accurately in the experimental treatment arm, given similar levels of other parameters that affect the performance of adjustment methods – in particular switching proportions. Therefore, we felt that simulating switching in both treatment arms would add unnecessary complexity to our study without any considerable added value.

As a further simplification in our simulation study, we focused on the first switch away from the treatment pathway of interest. In reality, patients are likely to switch more than once over the course of their disease pathway. The methods assessed in the study remain valid in the presence of additional switches. The IPCW method is unaffected by additional switches as censoring occurs at first switch away from the treatment pathway of interest, and for TSE the effect of subsequent treatments is implicitly adjusted for as part of the first adjustment. Hence, the findings are more broadly applicable than cases where patients can only switch on one occasion.

In addition, we note that the simulated prognostic characteristics represent a simplified version of the data that may be collected as part of a real RCT. We have included variables to represent baseline prognosis and time-dependent metastatic disease. The relationship between these variables and the propensity to switch to each treatment varied in strength, allowing for divergences in the average characteristics of patients switching to each treatment. In a real RCT, more variables may have been collected, resulting in the potential for greater divergence of the characteristics of patients that switch onto different treatments. If there were extreme differences between the patients that switch onto the different treatments, the IPCW combined approach may not provide an accurate estimate of average outcomes without switching. A simulation study with a more complex data generating mechanism would be required to test this.

It is also important to note that we tested applications of IPCW, TSEgest and TSEsimp that had correctly specified models, given the data generating mechanism. In reality, where data generating mechanisms are not known, models may be specified incorrectly, which is likely to result in more biased results. Finally, it is a limitation of our study that we did not consider coverage. Because the applications of TSEgest and TSEsimp in our study involved first estimating the treatment effect(s) associated with switching, then using these to derive counterfactual survival times, and then fitting flexible parametric models to these to estimate RMST, appropriate coverage estimates could only be derived by bootstrapping the entire adjustment process. Similar is true for IPCW, because a flexible parametric model is fitted to weighted survival times to estimate RMST. Bootstrapping entire adjustment processes is not feasible in simulation studies of this complexity that investigate many scenarios. Other simulation studies investigating switching adjustment methods have also been subject to this limitation, and have either reported coverage in a limited way or not at all.^4,10,11,23,24^ One previous study has demonstrated that using bootstrapping to derive confidence intervals for the adjustment methods results in good levels of coverage in scenarios where adjustment methods produce low bias.^26^ Therefore, we believe that the adjustment methods considered in our study would provide adequate coverage in scenarios where they produced low bias, but sub-optimal coverage in scenarios where bias was appreciable.

In conclusion, our study has shown that IPCW, TSEsimp and TSEgest adjustment methods are all capable of adjusting for the multiple types of treatment switching that may occur in RCTs. When using these methods to adjust for multiple switches, we recommend combining switchers into one group and adjusting for them together, rather than attempting to adjust for each type of switching separately. When choosing between adjustment methods, the factors that should be considered are the same as those that are important when dealing with just one type of switching – in particular, the IPCW method is prone to higher levels of bias than TSEsimp and TSEgest when switching proportions are high, if this results in weights with high values. IPCW, TSEsimp and TSEgest are all reliant on the no unmeasured confounding assumption, and TSEsimp is problematic if time-dependent confounding is present. TSEsimp and TSEgest should be applied with and without recensoring, because recensoring has the potential to cause bias. Fundamentally, it is possible to use these adjustment methods to adjust for multiple switches in RCTs, but, as with any adjustment analysis, care must be taken in the application of methods, assumptions must be considered on a case-by-case basis, and results must be interpreted with due caution.

## Supplemental Material

sj-docx-1-smm-10.1177_09622802241300049 - Supplemental material for Adjusting for switches to multiple treatments: Should switches be handled separately or combined? Supplemental material, sj-docx-1-smm-10.1177_09622802241300049 for Adjusting for switches to multiple treatments: Should switches be handled separately or combined? by Helen Bell Gorrod, Shahrul Mt-Isa, Jingyi Xuan, Kristel Vandormael, William Malbecq, Victoria Yorke-Edwards, Ian R White and Nicholas Latimer in Statistical Methods in Medical Research

sj-txt-2-smm-10.1177_09622802241300049 - Supplemental material for Adjusting for switches to multiple treatments: Should switches be handled separately or combined? Supplemental material, sj-txt-2-smm-10.1177_09622802241300049 for Adjusting for switches to multiple treatments: Should switches be handled separately or combined? by Helen Bell Gorrod, Shahrul Mt-Isa, Jingyi Xuan, Kristel Vandormael, William Malbecq, Victoria Yorke-Edwards, Ian R White and Nicholas Latimer in Statistical Methods in Medical Research
