# Supplementary material for: Adjusting for switches to multiple treatments: Should switches be handled separately or combined?
Source: Stat Methods Med Res. 2025 Jan 17;34(2):322–35. doi: 10.1177/09622802241300049 (PMC11874486; doi:10.1177/09622802241300049)
Supplement: sj-docx-1-smm-10.1177_09622802241300049 - Supplemental material for Adjusting for switches to multiple treatments: Should switches be handled separately or combined? [file sj-docx-1-smm-10.1177_09622802241300049.docx]

# Supplementary materials

## Appendix 1: Adjustment methods

**IPCW**

The IPCW approach works by censoring patients at the time of switch, then applying a weight to inflate non-switching patients to represent themselves and switchers. In the case of multiple treatments, there are different ways that weights can be estimated.

To apply the IPCW approach, the data is censored at time of switch. Switching models are estimated to generate the weights, and then the outcome model is estimated with weights applied. Weights can be estimated as stabilised or unstabilised weights, where unstablised weights require the estimation of a denominator and stabilised weights require the estimation of a numerator and a denominator.

IPCW-C: Treatments combined –

The treatments combined approach groups together all treatments that are switched to by control group patients. The dependent variable for the switching weight models is binary, 1 if switch and 0 otherwise.

Numerator:

The numerator switch model (for stabilised weights) is estimated on patients in the control group at all time points, *t*, from baseline to time of switch using binary logistic model.

$logit \left( p_{num}^{1}\left( XO \right) \right)=\beta_{0}+\beta V$ if control group=1

Where $XO$ represents a binary time-dependent indicator of switch, and V represents baseline prognosis. Following estimation of the logistic model, the probabilities of switching were obtained using the “predict, pr” post-estimation command in Stata 17. For each patient at each time point t, the cumulative probability of remaining unswitched is calculated, where $p^{1}$ equals the probability of switching.

$$P_{num}=\sum_{t}^{t0} (1-p_{num}^{1})$$

Denominator:

The denominator switch model was estimated for patients in the control group at time points where switching was possible (i.e. visits 1-6 post-progression).

$logit \left( p_{denom}^{1}\left( XO \right) \right)=\beta_{0}+\sum_{m} {\beta_{m}VM}_{t-1}$ if control group=1

Where $VM$ represents a set of interaction variables of baseline prognosis V and metastatic disease M at the previous time point, t-1, and $\beta_{m}$ represents the associated coefficients, and $\beta_{0}$ represents the constant term. Again, the probabilities of switching were obtained using the “predict, pr” post-estimation command in Stata 17. Cumulative probabilities were calculated for each patient i at each time point t, as follows.

$$P_{denom}=\sum_{t}^{t0} (1-p_{denom}^{1})$$

Weights:

Weights were calculated as follows and set equal to 1 for treatment group patients.

$$SW=\frac{P_{num}}{P_{denom}}$$

$$UW=\frac{1}{P_{denom}}$$

Outcome model:

A logistic model with a binary indicator of survival as the dependent variable and incorporating stabilised or unstabilised weights, was applied to the time dependent data censored at time of switch. Models with stabilised weights additionally included baseline confounders as explanatory variables.

IPCW_S: Treatments separate – Multinomial logistic model using P0

A multinomial logistic model can be applied which distinguishes between the treatments switched to in the dependent variable. The dependent variable is categorical, representing switches to treatments 1, 2…N and 0 if no switch occurs at time t.

Numerator:

The numerator switch model is estimated on patients in the control group at all time points, *t*, from baseline to time of switch using a multinomial logistic model.

$logit \left( p_{num}^{1}\left( {XO}_{M} \right) \right)=\beta_{0}+\beta V$ if control group=1

Where ${XO}_{M}$ represents a categorical time-dependent indicator of switch to treatments *1-n*, and 0 if no switch at time t. Following estimation of the multinomial logistic model, the probabilities of not switching (i.e. P(switch=0)) were obtained using the “predict, pr outcome(0)” post-estimation command in Stata 17. For each patient at each time point t, the cumulative probability of remaining unswitched is calculated, where $p^{0}$ equals the probability of no switching.

$$P=\sum_{t}^{t0} (p^{0})$$

Denominator:

The denominator switch model was estimated for patients in the control group at time points where switching was possible (i.e. visits 1-6 post-progression).

$logit \left( p_{denom}^{1}\left( {XO}_{M} \right) \right)=\beta_{0}+\sum_{m} {\beta VM}_{t-1}$ if control group=1

Again, the probabilities of switching were obtained using the “predict, pr” post-estimation command in Stata 17. Cumulative probabilities were calculated for each patient i at each time point t. Weights were calculated using cumulative probabilities derived from the numerator and denominator models as described for application 1. Outcome models were also applied in the same way as described for application 1.

**TSE**

TSE can be applied using either as the simple TSE method, or TSE with g-estimation. Simple TSE requires that switching occurs shortly after a disease-related secondary baseline. In this study disease progression is used as the secondary baseline.

TSEsimp-C: combined treatments

An accelerated failure time (AFT) model with Weibull distribution was applied to estimate the post-progression time-ratio treatment effect of switchers compared to non-switchers in the control group. The data was set up with one observation per patient, i, and time point, t.

$$log\left( T_{i,t} \right)=\beta_{0}+\beta_{1}{XO}_{i,t}^{tdc}+\beta_{2}V_{i}+ z_{i}$$

Where $T_{i,t}$is the observed survival time for patient i at time t, ${XO}_{i,t}^{tdc}$ represents treatment received (switch vs no switch) at time t.

The data was transformed to represent one time to event observation per patient. The estimated treatment effect, derived as the coefficient on the treatment variable $-\beta_{1}$in the AFT model, was used as follows to calculate adjusted survival times for switchers.

$$U_{i}=T_{ci}+e^{\psi}T_{Si}$$

Where $U_{i}$represents counterfactual survival times, $T_{ci}$represents time on the control group treatment, $T_{Si}$ represents time on the switched to treatment, and $\psi$ represents the average acceleration factor captured by the coefficient on ${XO}_{i,t}^{tdc}$ in the AFT model.

Recensoring was applied by firstly identifying $D_{i}$, the minimum of the administrative censoring time C and C$e^{\psi}$. This minimum value $D_{i}$replaces the counterfactual survival time, $U_{i}$, if $D_{i}<U_{i}$.

A flexible parametric outcome model was applied to the counterfactual data, and restricted mean survival times (RMST) were subsequently estimated.

TSEgest-C - combined treatments

TSE application 1 can also be applied using g-estimation for accelerated failure time models.[10] The g-estimation method involves fitting a series of logistic models with for different values of $\psi$, with the dependent variable ${XO}_{t}^{tdc}$ indicating treatment received at time t (switched vs control treatment) and controlling for confounders.

$$logit\left( {XO}_{i,t}^{tdc} \right)=U\left( \psi\right)+\sum_{m} \beta_{m}{VM}_{m, i, t-1}+ \sum_{j} \beta_{j}X_{j,i,t}$$

$U\left( \psi\right)$ represents the counterfactual survival time for a specific value of $\psi$. G-estimation was applied using the stgest command in Stata 17.[26]

The data was transformed to represent one time to event observation per patient. The estimated value $\psi$ derived from the AFT model using g-estimation, was used to calculate adjusted survival times for switchers, as follows.

$$U_{i}=T_{ci}+e^{\psi}T_{Si}$$

Where $U_{i}$represents counterfactual survival times, $T_{ci}$represents time on the control group treatment, $T_{Si}$ represents time on the switched to treatment. Recensoring was applied, as previously described, in the applications with recensoring, and the outcome model was applied to the adjusted data.

TSEsimp-S – separate treatment effects (one model)

An accelerated failure time (AFT) model with Weibull distribution was applied to estimate the post-progression time-ratio treatment effect of switchers compared to non-switchers in the control group. The data was set up with one observation per patient, i, and time point, t.

$$log\left( T_{i,t} \right)=\beta_{0}+\beta_{1}{XOT1}_{i,t}^{tdc}+\beta_{2}{XOT2}_{i,t}^{tdc}\ldots+\beta_{2}{XOTN}_{i,t}^{tdc}+\beta_{N+1}V_{i}+ Z_{i}$$

Where $T_{i,t}$is the observed survival time for patient i at time t, ${XOT1}_{i,t}^{tdc}$ represents treatment 1 vs otherwise at time t, and ${XOT2}_{i,t}^{tdc}$ represents switch to treatment 2 vs otherwise at time t.

The data was transformed to represent one time to event observation per patient. The estimated treatment effect, derived as the coefficient on the treatment 1 variable $-\beta_{1}$in the AFT model, was used as follows to calculate adjusted survival times for switchers to treatment 1.

$U_{i}=T_{ci}+e^{\psi1}T_{Si}$ if ${XO}_{i}=1$

Where $U_{i}$represents counterfactual survival times, $T_{ci}$represents time on the control group treatment, $T_{Si}$ represents time on the switched to treatment, $\psi1$ represents the average acceleration factor captured by the coefficient on ${XOT1}_{i,t}^{tdc}$ in the AFT model, and ${XO}_{i}=1$ indicates if patient i switched to treatment 1.

Similarly, the estimated treatment effects $-\beta_{2}$to $-\beta_{N}$from the AFT model, were used as follows to calculate adjusted survival times for switchers to treatment 2 to N.

$U_{i}=T_{ci}+e^{\psi n}T_{Si}$ if ${XO}_{i}=n$

Recensoring was applied by firstly identifying $D_{i}$, the minimum of the administrative censoring time C and C$e^{\psi n}$, where $\psi n$ represents the $\psi$ with the largest treatment effect compared to the $\psi$ for the other switched to treatments. This minimum value $D_{i}$replaces the counterfactual survival time, $U_{i}$, if $D_{i}<U_{i}$.

A flexible parametric outcome model was applied to the counterfactual data, and RMST was calculated.

TSE application 2 was not estimated using g-estimation, because only one value of psi is obtained from the standard g-estimation model.

TSEgest-S –separate treatments (separate models)

A set of *N* accelerated failure time (AFT) models were applied using g-estimation to estimate the post-progression time-ratio treatment effects ψ1,ψ2,…ψN of switchers to treatment n compared to non-switchers in the control group, where n represents switched to treatments 1,2,..N. The data was set up with one observation per patient, i, and time point, t.

$logit\left( {XO}_{i,t}^{tdc} \right)=U\left( \psi\right)+\sum_{m} \beta_{m}{VM}_{m, i, t-1}+ \sum_{j} \beta_{j}X_{j,i,t}$ if ${XO}_{i}=0$ or ${XO}_{i}=n$

Where $T_{i,t}$is the observed survival time for patient i at time t, ${XOTn}_{i,t}^{tdc}$ represents treatment received (treatment *n* vs no switch) at time t.

The data was transformed to represent one time to event observation per patient. The estimated values of ψ1,ψ2,…ψN were used as follows to calculate adjusted survival times for switchers to each treatment n.

$U_{i}=T_{ci}+e^{\psi n}T_{Si}$ if ${XO}_{i}=n$

Where $U_{i}$represents counterfactual survival times, $T_{ci}$represents time on the control group treatment, $T_{Si}$ represents time on the switched to treatment, and $\psi n$ represents the set of average acceleration factors $\psi1,\psi2,\ldots\psi N$ derived from the switch to treatment n vs no switch AFT models.

Recensoring was applied as previously described for TSE application 2. Again, a flexible parametric outcome model was applied to the counterfactual data and RMST was estimated

**Appendix 2: Tables of results by scenario**

Table A2.1: Results for scenario 1

| Method & application | Bias in RMST | Bias  MCSE | EmpSE | EmpSE  MCSE | RMSE | RMSE  MCSE |
| --- | --- | --- | --- | --- | --- | --- |
| No switch | -0.022 | 0.122 | 3.858 | 0.086 | 3.856 | 0.082 |
| ITT | 8.751 | 0.128 | 4.037 | 0.090 | 9.637 | 0.119 |
| TSEsimp-C | -2.301 | 0.206 | 6.519 | 0.146 | 6.910 | 0.161 |
| TSEsimp-S | -2.704 | 0.258 | 8.155 | 0.182 | 8.588 | 0.373 |
| TSEsimp-C without rec | 1.902 | 0.127 | 4.031 | 0.090 | 4.455 | 0.095 |
| TSEsimp-S without rec | 1.881 | 0.127 | 4.027 | 0.090 | 4.443 | 0.094 |
| TSEgest-C | -6.346 | 0.298 | 9.433 | 0.211 | 11.365 | 0.241 |
| TSEgest-S | -5.474 | 0.560 | 17.371 | 0.396 | 17.884 | 0.912 |
| TSEgest-C without rec | 0.412 | 0.127 | 4.006 | 0.090 | 4.025 | 0.086 |
| TSEgest-S without rec | 0.459 | 0.130 | 4.025 | 0.092 | 4.059 | 0.088 |
| IPCW-C unstabilised | -0.166 | 0.128 | 4.032 | 0.090 | 4.034 | 0.086 |
| IPCW-C stabilised | 1.431 | 0.130 | 4.108 | 0.092 | 4.349 | 0.093 |
| IPCW-S unstabilised | -0.161 | 0.129 | 4.040 | 0.092 | 4.033 | 0.087 |
| IPCW-S stabilised | 1.435 | 0.132 | 4.123 | 0.093 | 4.349 | 0.094 |

Table A2.2: Results for scenario 2

| Method & application | Bias in RMST | Bias  MCSE | EmpSE | EmpSE  MCSE | RMSE | RMSE  MCSE |
| --- | --- | --- | --- | --- | --- | --- |
| No switch | -0.045 | 0.121 | 3.820 | 0.085 | 3.818 | 0.086 |
| ITT | 7.630 | 0.126 | 3.995 | 0.089 | 8.612 | 0.122 |
| TSEsimp-C | -1.382 | 0.181 | 5.724 | 0.128 | 5.886 | 0.140 |
| TSEsimp-S | -3.138 | 0.272 | 8.589 | 0.192 | 9.140 | 0.365 |
| TSEsimp-C without rec | 1.663 | 0.127 | 4.017 | 0.090 | 4.346 | 0.099 |
| TSEsimp-S without rec | 1.668 | 0.127 | 4.022 | 0.090 | 4.352 | 0.099 |
| TSEgest-C | -5.604 | 0.261 | 8.265 | 0.185 | 9.982 | 0.223 |
| TSEgest-S | -4.607 | 0.712 | 21.924 | 0.504 | 21.837 | 1.032 |
| TSEgest-C without rec | 0.224 | 0.127 | 4.017 | 0.090 | 4.021 | 0.090 |
| TSEgest-S without rec | 0.352 | 0.130 | 4.005 | 0.092 | 4.046 | 0.092 |
| IPCW-C unstabilised | -0.132 | 0.127 | 4.019 | 0.090 | 4.019 | 0.089 |
| IPCW-C stabilised | 1.508 | 0.129 | 4.091 | 0.092 | 4.358 | 0.098 |
| IPCW-S unstabilised | -0.153 | 0.128 | 4.014 | 0.090 | 4.016 | 0.090 |
| IPCW-S stabilised | 1.485 | 0.130 | 4.091 | 0.092 | 4.355 | 0.099 |

Table A2.3: Results for scenario 3

| Method & application | Bias in RMST | Bias  MCSE | EmpSE | EmpSE  MCSE | RMSE | RMSE  MCSE |
| --- | --- | --- | --- | --- | --- | --- |
| No switch | -0.060 | 0.118 | 3.746 | 0.084 | 3.745 | 0.085 |
| ITT | 8.310 | 0.128 | 4.041 | 0.090 | 9.239 | 0.122 |
| TSEsimp-C | -2.001 | 0.185 | 5.860 | 0.131 | 6.190 | 0.147 |
| TSEsimp-S | -2.967 | 0.242 | 7.641 | 0.171 | 8.193 | 0.226 |
| TSEsimp-C without rec | 1.818 | 0.126 | 3.996 | 0.089 | 4.388 | 0.099 |
| TSEsimp-S without rec | 1.800 | 0.126 | 3.991 | 0.089 | 4.377 | 0.099 |
| TSEgest-C | -5.811 | 0.283 | 8.937 | 0.200 | 10.651 | 0.237 |
| TSEgest-S | -5.871 | 0.426 | 13.459 | 0.302 | 14.654 | 0.667 |
| TSEgest-C without rec | 0.327 | 0.125 | 3.939 | 0.088 | 3.948 | 0.090 |
| TSEgest-S without rec | 0.565 | 0.125 | 3.954 | 0.089 | 4.006 | 0.092 |
| IPCW-C unstabilised | -0.159 | 0.125 | 3.968 | 0.089 | 3.970 | 0.088 |
| IPCW-C stabilised | 1.437 | 0.127 | 4.029 | 0.090 | 4.276 | 0.098 |
| IPCW-S unstabilised | -0.161 | 0.126 | 3.974 | 0.089 | 3.973 | 0.088 |
| IPCW-S stabilised | 1.433 | 0.128 | 4.033 | 0.090 | 4.277 | 0.098 |

Table A2.4: Results for scenario 4

| Method & application | Bias in RMST | Bias  MCSE | EmpSE | EmpSE  MCSE | RMSE | RMSE  MCSE |
| --- | --- | --- | --- | --- | --- | --- |
| No switch | -0.107 | 0.125 | 3.960 | 0.089 | 3.960 | 0.086 |
| ITT | 8.246 | 0.130 | 4.111 | 0.092 | 9.213 | 0.122 |
| TSEsimp-C | -2.113 | 0.194 | 6.125 | 0.137 | 6.476 | 0.157 |
| TSEsimp-S | -3.129 | 0.270 | 8.538 | 0.191 | 9.089 | 0.300 |
| TSEsimp-C without rec | 1.691 | 0.129 | 4.094 | 0.092 | 4.427 | 0.095 |
| TSEsimp-S without rec | 1.695 | 0.130 | 4.097 | 0.092 | 4.432 | 0.095 |
| TSEgest-C | -6.036 | 0.308 | 9.742 | 0.218 | 11.456 | 0.252 |
| TSEgest-S | -5.943 | 0.496 | 15.656 | 0.351 | 16.714 | 0.699 |
| TSEgest-C without rec | 0.176 | 0.128 | 4.039 | 0.090 | 4.041 | 0.087 |
| TSEgest-S without rec | 0.417 | 0.129 | 4.064 | 0.091 | 4.078 | 0.088 |
| IPCW-C unstabilised | -0.241 | 0.129 | 4.065 | 0.091 | 4.070 | 0.091 |
| IPCW-C stabilised | 1.341 | 0.131 | 4.131 | 0.092 | 4.341 | 0.094 |
| IPCW-S unstabilised | -0.245 | 0.128 | 4.063 | 0.091 | 4.069 | 0.091 |
| IPCW-S stabilised | 1.337 | 0.131 | 4.131 | 0.092 | 4.340 | 0.094 |

Table A2.5: Results for scenario 5

| Method & application | Bias in RMST | Bias  MCSE | EmpSE | EmpSE  MCSE | RMSE | RMSE  MCSE |
| --- | --- | --- | --- | --- | --- | --- |
| No switch | -0.073 | 0.124 | 3.932 | 0.088 | 3.930 | 0.087 |
| ITT | 24.983 | 0.139 | 4.408 | 0.099 | 25.369 | 0.136 |
| TSEsimp-C | 5.169 | 0.227 | 7.175 | 0.161 | 8.840 | 0.177 |
| TSEsimp-S | 2.689 | 0.248 | 7.841 | 0.175 | 8.285 | 0.167 |
| TSEsimp-C without rec | 8.531 | 0.169 | 5.345 | 0.120 | 10.066 | 0.158 |
| TSEsimp-S without rec | 7.322 | 0.167 | 5.276 | 0.118 | 9.024 | 0.153 |
| TSEgest-C | -4.683 | 0.344 | 10.853 | 0.243 | 11.804 | 0.275 |
| TSEgest-S | -5.898 | 0.420 | 13.266 | 0.297 | 14.510 | 0.319 |
| TSEgest-C without rec | 2.265 | 0.165 | 5.208 | 0.117 | 5.671 | 0.127 |
| TSEgest-S without rec | 2.030 | 0.164 | 5.183 | 0.116 | 5.574 | 0.131 |
| IPCW-C unstabilised | 16.501 | 0.585 | 18.195 | 0.414 | 24.593 | 0.493 |
| IPCW-C stabilised | 10.589 | 0.422 | 13.133 | 0.299 | 16.909 | 0.404 |
| IPCW-S unstabilised | 15.802 | 0.675 | 18.024 | 0.477 | 25.464 | 0.585 |
| IPCW-S stabilised | 10.177 | 0.484 | 12.935 | 0.343 | 17.780 | 0.490 |

Table A2.6: Results for scenario 6

| Method & application | Bias in RMST | Bias  MCSE | EmpSE | EmpSE  MCSE | RMSE | RMSE  MCSE |
| --- | --- | --- | --- | --- | --- | --- |
| No switch | -0.298 | 0.118 | 3.735 | 0.084 | 3.745 | 0.079 |
| ITT | 21.321 | 0.131 | 4.154 | 0.093 | 21.722 | 0.130 |
| TSEsimp-C | 2.149 | 0.218 | 6.889 | 0.154 | 7.213 | 0.153 |
| TSEsimp-S | 2.291 | 0.254 | 8.034 | 0.180 | 8.350 | 0.208 |
| TSEsimp-C without rec | 5.586 | 0.158 | 4.993 | 0.112 | 7.491 | 0.141 |
| TSEsimp-S without rec | 6.322 | 0.163 | 5.158 | 0.115 | 8.158 | 0.149 |
| TSEgest-C | -6.352 | 0.304 | 9.591 | 0.215 | 11.491 | 0.251 |
| TSEgest-S | -6.056 | 0.400 | 12.631 | 0.283 | 13.988 | 0.528 |
| TSEgest-C without rec | 0.104 | 0.158 | 4.989 | 0.112 | 4.988 | 0.119 |
| TSEgest-S without rec | 1.152 | 0.163 | 5.162 | 0.116 | 5.281 | 0.140 |
| IPCW-C unstabilised | 16.178 | 0.589 | 18.386 | 0.416 | 24.546 | 0.480 |
| IPCW-C stabilised | 10.168 | 0.423 | 13.221 | 0.299 | 16.705 | 0.400 |
| IPCW-S unstabilised | 15.927 | 0.693 | 18.461 | 0.491 | 25.530 | 0.573 |
| IPCW-S stabilised | 10.015 | 0.500 | 13.301 | 0.353 | 17.869 | 0.486 |

Table A2.7: Results for scenario 7

| Method & application | Bias in RMST | Bias  MCSE | EmpSE | EmpSE  MCSE | RMSE | RMSE  MCSE |
| --- | --- | --- | --- | --- | --- | --- |
| No switch | 0.158 | 0.124 | 3.912 | 0.088 | 3.914 | 0.087 |
| ITT | 24.021 | 0.137 | 4.345 | 0.097 | 24.410 | 0.136 |
| TSEsimp-C | 4.542 | 0.217 | 6.851 | 0.153 | 8.217 | 0.164 |
| TSEsimp-S | 2.752 | 0.248 | 7.856 | 0.176 | 8.321 | 0.188 |
| TSEsimp-C without rec | 7.949 | 0.165 | 5.220 | 0.117 | 9.509 | 0.155 |
| TSEsimp-S without rec | 7.246 | 0.164 | 5.186 | 0.116 | 8.909 | 0.153 |
| TSEgest-C | -4.535 | 0.343 | 10.848 | 0.243 | 11.757 | 0.279 |
| TSEgest-S | -4.259 | 0.390 | 12.305 | 0.276 | 12.989 | 0.330 |
| TSEgest-C without rec | 1.958 | 0.161 | 5.103 | 0.114 | 5.482 | 0.125 |
| TSEgest-S without rec | 3.123 | 0.155 | 4.897 | 0.110 | 5.804 | 0.124 |
| IPCW-C unstabilised | 16.616 | 0.593 | 18.485 | 0.419 | 24.913 | 0.496 |
| IPCW-C stabilised | 10.711 | 0.432 | 13.467 | 0.305 | 17.228 | 0.422 |
| IPCW-S unstabilised | 16.123 | 0.653 | 18.678 | 0.462 | 24.961 | 0.542 |
| IPCW-S stabilised | 10.502 | 0.472 | 13.506 | 0.334 | 17.258 | 0.459 |

Table A2.8: Results for scenario 8

| Method & application | Bias in RMST | Bias  MCSE | EmpSE | EmpSE  MCSE | RMSE | RMSE  MCSE |
| --- | --- | --- | --- | --- | --- | --- |
| No switch | -0.057 | 0.121 | 3.813 | 0.085 | 3.812 | 0.083 |
| ITT | 23.566 | 0.133 | 4.208 | 0.094 | 23.938 | 0.131 |
| TSEsimp-C | 2.639 | 0.223 | 7.037 | 0.157 | 7.512 | 0.168 |
| TSEsimp-S | 2.390 | 0.276 | 8.724 | 0.195 | 9.041 | 0.201 |
| TSEsimp-C without rec | 6.466 | 0.156 | 4.939 | 0.110 | 8.135 | 0.141 |
| TSEsimp-S without rec | 7.151 | 0.158 | 5.011 | 0.112 | 8.731 | 0.145 |
| TSEgest-C | -6.628 | 0.333 | 10.523 | 0.236 | 12.420 | 0.283 |
| TSEgest-S | -4.227 | 0.385 | 12.118 | 0.272 | 12.774 | 0.324 |
| TSEgest-C without rec | 0.797 | 0.155 | 4.908 | 0.110 | 4.967 | 0.122 |
| TSEgest-S without rec | 2.941 | 0.156 | 4.902 | 0.110 | 5.709 | 0.130 |
| IPCW-C unstabilised | 16.070 | 0.605 | 18.843 | 0.428 | 24.866 | 0.490 |
| IPCW-C stabilised | 10.438 | 0.446 | 13.902 | 0.316 | 17.463 | 0.421 |
| IPCW-S unstabilised | 15.787 | 0.654 | 18.778 | 0.463 | 24.948 | 0.533 |
| IPCW-S stabilised | 10.250 | 0.479 | 13.768 | 0.339 | 17.548 | 0.458 |

Table A2.9: Results for scenario 9

| Method & application | Bias in RMST | Bias  MCSE | EmpSE | EmpSE  MCSE | RMSE | RMSE  MCSE |
| --- | --- | --- | --- | --- | --- | --- |
| No switch | -0.029 | 0.084 | 2.667 | 0.060 | 2.666 | 0.062 |
| ITT | 8.780 | 0.088 | 2.788 | 0.062 | 9.211 | 0.087 |
| TSEsimp-C | -2.392 | 0.140 | 4.421 | 0.099 | 5.024 | 0.109 |
| TSEsimp-S | -2.981 | 0.166 | 5.244 | 0.117 | 6.029 | 0.136 |
| TSEsimp-C without rec | 1.956 | 0.088 | 2.790 | 0.062 | 3.406 | 0.072 |
| TSEsimp-S without rec | 1.937 | 0.088 | 2.788 | 0.062 | 3.394 | 0.072 |
| TSEgest-C | -6.539 | 0.214 | 6.765 | 0.151 | 9.406 | 0.186 |
| TSEgest-S | -6.928 | 0.328 | 10.362 | 0.232 | 12.443 | 0.800 |
| TSEgest-C without rec | 0.430 | 0.088 | 2.772 | 0.062 | 2.804 | 0.063 |
| TSEgest-S without rec | 0.563 | 0.088 | 2.778 | 0.062 | 2.834 | 0.063 |
| IPCW-C unstabilised | -0.081 | 0.088 | 2.774 | 0.062 | 2.773 | 0.065 |
| IPCW-C stabilised | 1.486 | 0.090 | 2.836 | 0.063 | 3.201 | 0.070 |
| IPCW-S unstabilised | -0.081 | 0.088 | 2.773 | 0.062 | 2.773 | 0.065 |
| IPCW-S stabilised | 1.485 | 0.090 | 2.836 | 0.063 | 3.200 | 0.070 |

Table A2.10: Results for scenario 10

| Method & application | Bias in RMST | Bias  MCSE | EmpSE | EmpSE  MCSE | RMSE | RMSE  MCSE |
| --- | --- | --- | --- | --- | --- | --- |
| No switch | -0.058 | 0.086 | 2.726 | 0.061 | 2.725 | 0.067 |
| ITT | 7.544 | 0.092 | 2.896 | 0.065 | 8.080 | 0.088 |
| TSEsimp-C | -1.744 | 0.128 | 4.043 | 0.090 | 4.402 | 0.097 |
| TSEsimp-S | -3.235 | 0.176 | 5.556 | 0.124 | 6.427 | 0.172 |
| TSEsimp-C without rec | 1.629 | 0.091 | 2.881 | 0.064 | 3.309 | 0.075 |
| TSEsimp-S without rec | 1.636 | 0.091 | 2.883 | 0.065 | 3.314 | 0.075 |
| TSEgest-C | -5.653 | 0.186 | 5.868 | 0.131 | 8.146 | 0.165 |
| TSEgest-S | -6.622 | 0.439 | 13.873 | 0.311 | 15.351 | 1.015 |
| TSEgest-C without rec | 0.187 | 0.090 | 2.862 | 0.064 | 2.866 | 0.070 |
| TSEgest-S without rec | 0.318 | 0.091 | 2.875 | 0.064 | 2.888 | 0.070 |
| IPCW-C unstabilised | -0.164 | 0.091 | 2.870 | 0.064 | 2.874 | 0.069 |
| IPCW-C stabilised | 1.407 | 0.092 | 2.913 | 0.065 | 3.234 | 0.073 |
| IPCW-S unstabilised | -0.165 | 0.091 | 2.870 | 0.064 | 2.873 | 0.069 |
| IPCW-S stabilised | 1.405 | 0.092 | 2.911 | 0.065 | 3.232 | 0.073 |

Table A2.11: Results for scenario 11

| Method & application | Bias in RMST | Bias  MCSE | EmpSE | EmpSE  MCSE | RMSE | RMSE  MCSE |
| --- | --- | --- | --- | --- | --- | --- |
| No switch | -0.042 | 0.086 | 2.732 | 0.061 | 2.731 | 0.063 |
| ITT | 8.304 | 0.093 | 2.933 | 0.066 | 8.806 | 0.091 |
| TSEsimp-C | -2.008 | 0.132 | 4.168 | 0.093 | 4.624 | 0.104 |
| TSEsimp-S | -3.132 | 0.156 | 4.935 | 0.110 | 5.843 | 0.132 |
| TSEsimp-C without rec | 1.847 | 0.090 | 2.861 | 0.064 | 3.404 | 0.073 |
| TSEsimp-S without rec | 1.830 | 0.090 | 2.858 | 0.064 | 3.392 | 0.073 |
| TSEgest-C | -6.345 | 0.201 | 6.370 | 0.143 | 8.988 | 0.167 |
| TSEgest-S | -6.595 | 0.243 | 7.685 | 0.172 | 10.124 | 0.204 |
| TSEgest-C without rec | 0.365 | 0.089 | 2.821 | 0.063 | 2.844 | 0.064 |
| TSEgest-S without rec | 0.613 | 0.090 | 2.831 | 0.063 | 2.896 | 0.065 |
| IPCW-C unstabilised | -0.118 | 0.089 | 2.827 | 0.063 | 2.828 | 0.064 |
| IPCW-C stabilised | 1.433 | 0.091 | 2.888 | 0.065 | 3.223 | 0.070 |
| IPCW-S unstabilised | -0.120 | 0.089 | 2.828 | 0.063 | 2.829 | 0.064 |
| IPCW-S stabilised | 1.430 | 0.091 | 2.889 | 0.065 | 3.222 | 0.070 |

Table A2.12: Results for scenario 12

| Method & application | Bias in RMST | Bias  MCSE | EmpSE | EmpSE  MCSE | RMSE | RMSE  MCSE |
| --- | --- | --- | --- | --- | --- | --- |
| No switch | 0.048 | 0.088 | 2.770 | 0.062 | 2.769 | 0.062 |
| ITT | 8.318 | 0.093 | 2.956 | 0.066 | 8.827 | 0.091 |
| TSEsimp-C | -2.070 | 0.135 | 4.272 | 0.096 | 4.746 | 0.109 |
| TSEsimp-S | -3.297 | 0.180 | 5.681 | 0.127 | 6.566 | 0.162 |
| TSEsimp-C without rec | 1.898 | 0.093 | 2.931 | 0.066 | 3.490 | 0.076 |
| TSEsimp-S without rec | 1.905 | 0.093 | 2.932 | 0.066 | 3.495 | 0.076 |
| TSEgest-C | -6.123 | 0.202 | 6.393 | 0.143 | 8.850 | 0.180 |
| TSEgest-S | -6.881 | 0.266 | 8.407 | 0.188 | 10.861 | 0.243 |
| TSEgest-C without rec | 0.383 | 0.091 | 2.892 | 0.065 | 2.916 | 0.066 |
| TSEgest-S without rec | 0.651 | 0.092 | 2.915 | 0.065 | 2.986 | 0.068 |
| IPCW-C unstabilised | -0.047 | 0.092 | 2.916 | 0.065 | 2.915 | 0.066 |
| IPCW-C stabilised | 1.514 | 0.094 | 2.973 | 0.067 | 3.335 | 0.073 |
| IPCW-S unstabilised | -0.047 | 0.092 | 2.916 | 0.065 | 2.915 | 0.066 |
| IPCW-S stabilised | 1.513 | 0.094 | 2.972 | 0.066 | 3.333 | 0.073 |

Table A2.13: Results for scenario 13

| Method & application | Bias in RMST | Bias  MCSE | EmpSE | EmpSE  MCSE | RMSE | RMSE  MCSE |
| --- | --- | --- | --- | --- | --- | --- |
| No switch | -0.042 | 0.087 | 2.737 | 0.061 | 2.736 | 0.065 |
| ITT | 24.966 | 0.096 | 3.048 | 0.068 | 25.151 | 0.096 |
| TSEsimp-C | 4.741 | 0.157 | 4.967 | 0.111 | 6.865 | 0.134 |
| TSEsimp-S | 2.527 | 0.173 | 5.463 | 0.122 | 6.017 | 0.134 |
| TSEsimp-C without rec | 8.463 | 0.117 | 3.686 | 0.082 | 9.231 | 0.116 |
| TSEsimp-S without rec | 7.298 | 0.115 | 3.641 | 0.081 | 8.155 | 0.114 |
| TSEgest-C | -5.302 | 0.234 | 7.376 | 0.166 | 9.069 | 0.194 |
| TSEgest-S | -6.886 | 0.289 | 9.123 | 0.204 | 11.420 | 0.244 |
| TSEgest-C without rec | 2.332 | 0.118 | 3.708 | 0.083 | 4.399 | 0.105 |
| TSEgest-S without rec | 2.011 | 0.116 | 3.679 | 0.082 | 4.189 | 0.100 |
| IPCW-C unstabilised | 9.958 | 0.479 | 14.964 | 0.339 | 18.111 | 0.433 |
| IPCW-C stabilised | 6.078 | 0.310 | 9.676 | 0.219 | 11.466 | 0.329 |
| IPCW-S unstabilised | 9.508 | 0.524 | 14.529 | 0.370 | 19.132 | 0.520 |
| IPCW-S stabilised | 5.937 | 0.337 | 9.349 | 0.238 | 12.587 | 0.433 |

Table A2.14: Results for scenario 14

| Method & application | Bias in RMST | Bias  MCSE | EmpSE | EmpSE  MCSE | RMSE | RMSE  MCSE |
| --- | --- | --- | --- | --- | --- | --- |
| No switch | -0.051 | 0.085 | 2.690 | 0.060 | 2.689 | 0.060 |
| ITT | 21.454 | 0.093 | 2.942 | 0.066 | 21.655 | 0.092 |
| TSEsimp-C | 2.124 | 0.149 | 4.718 | 0.106 | 5.172 | 0.109 |
| TSEsimp-S | 1.631 | 0.177 | 5.598 | 0.125 | 5.828 | 0.127 |
| TSEsimp-C without rec | 5.603 | 0.108 | 3.415 | 0.076 | 6.561 | 0.103 |
| TSEsimp-S without rec | 6.286 | 0.111 | 3.525 | 0.079 | 7.206 | 0.107 |
| TSEgest-C | -6.988 | 0.214 | 6.730 | 0.151 | 9.661 | 0.194 |
| TSEgest-S | -6.189 | 0.241 | 7.599 | 0.171 | 9.769 | 0.204 |
| TSEgest-C without rec | 0.268 | 0.110 | 3.455 | 0.078 | 3.476 | 0.086 |
| TSEgest-S without rec | 1.205 | 0.113 | 3.577 | 0.080 | 3.765 | 0.096 |
| IPCW-C unstabilised | 10.395 | 0.482 | 15.089 | 0.341 | 18.512 | 0.435 |
| IPCW-C stabilised | 6.253 | 0.308 | 9.617 | 0.218 | 11.541 | 0.325 |
| IPCW-S unstabilised | 9.767 | 0.537 | 14.931 | 0.380 | 19.240 | 0.507 |
| IPCW-S stabilised | 5.942 | 0.341 | 9.482 | 0.241 | 12.240 | 0.408 |

Table A2.15: Results for scenario 15

| Method & application | Bias in RMST | Bias  MCSE | EmpSE | EmpSE  MCSE | RMSE | RMSE  MCSE |
| --- | --- | --- | --- | --- | --- | --- |
| No switch | -0.187 | 0.084 | 2.649 | 0.059 | 2.655 | 0.058 |
| ITT | 23.660 | 0.089 | 2.819 | 0.063 | 23.827 | 0.089 |
| TSEsimp-C | 4.177 | 0.156 | 4.934 | 0.110 | 6.463 | 0.119 |
| TSEsimp-S | 2.528 | 0.171 | 5.401 | 0.121 | 5.961 | 0.120 |
| TSEsimp-C without rec | 7.619 | 0.111 | 3.513 | 0.079 | 8.389 | 0.106 |
| TSEsimp-S without rec | 6.895 | 0.110 | 3.487 | 0.078 | 7.725 | 0.104 |
| TSEgest-C | -5.947 | 0.222 | 7.006 | 0.157 | 9.173 | 0.203 |
| TSEgest-S | -5.235 | 0.264 | 8.350 | 0.187 | 9.852 | 0.231 |
| TSEgest-C without rec | 1.636 | 0.109 | 3.430 | 0.077 | 3.830 | 0.081 |
| TSEgest-S without rec | 2.764 | 0.105 | 3.336 | 0.075 | 4.331 | 0.084 |
| IPCW-C unstabilised | 10.070 | 0.476 | 14.789 | 0.336 | 18.313 | 0.423 |
| IPCW-C stabilised | 5.981 | 0.305 | 9.471 | 0.215 | 11.446 | 0.321 |
| IPCW-S unstabilised | 10.282 | 0.472 | 14.737 | 0.334 | 18.258 | 0.471 |
| IPCW-S stabilised | 6.141 | 0.302 | 9.438 | 0.214 | 11.306 | 0.320 |

Table A2.16: Results for scenario 16

| Method & application | Bias in RMST | Bias  MCSE | EmpSE | EmpSE  MCSE | RMSE | RMSE  MCSE |
| --- | --- | --- | --- | --- | --- | --- |
| No switch | -0.129 | 0.084 | 2.661 | 0.060 | 2.663 | 0.060 |
| ITT | 23.415 | 0.091 | 2.880 | 0.064 | 23.591 | 0.091 |
| TSEsimp-C | 2.207 | 0.155 | 4.902 | 0.110 | 5.374 | 0.111 |
| TSEsimp-S | 1.988 | 0.183 | 5.774 | 0.129 | 6.104 | 0.136 |
| TSEsimp-C without rec | 6.280 | 0.110 | 3.483 | 0.078 | 7.180 | 0.105 |
| TSEsimp-S without rec | 6.950 | 0.112 | 3.546 | 0.079 | 7.801 | 0.108 |
| TSEgest-C | -7.727 | 0.242 | 7.643 | 0.171 | 10.847 | 0.218 |
| TSEgest-S | -5.196 | 0.248 | 7.835 | 0.176 | 9.387 | 0.205 |
| TSEgest-C without rec | 0.595 | 0.109 | 3.438 | 0.077 | 3.522 | 0.085 |
| TSEgest-S without rec | 2.704 | 0.108 | 3.418 | 0.077 | 4.356 | 0.093 |
| IPCW-C unstabilised | 10.471 | 0.468 | 14.674 | 0.331 | 18.196 | 0.436 |
| IPCW-C stabilised | 6.558 | 0.310 | 9.730 | 0.220 | 11.812 | 0.344 |
| IPCW-S unstabilised | 10.506 | 0.474 | 14.663 | 0.335 | 18.248 | 0.443 |
| IPCW-S stabilised | 6.582 | 0.313 | 9.682 | 0.221 | 11.843 | 0.348 |

Table A2.17: Results for scenario 17

| Method & application | Bias in RMST | Bias  MCSE | EmpSE | EmpSE  MCSE | RMSE | RMSE  MCSE |
| --- | --- | --- | --- | --- | --- | --- |
| No switch | 0.060 | 0.097 | 3.070 | 0.069 | 3.069 | 0.069 |
| ITT | 5.929 | 0.098 | 3.108 | 0.070 | 6.694 | 0.093 |
| TSEsimp-C | -2.526 | 0.249 | 7.885 | 0.176 | 8.276 | 0.257 |
| TSEsimp-S | -3.322 | 0.314 | 9.916 | 0.222 | 10.453 | 0.320 |
| TSEsimp-C without rec | 1.732 | 0.101 | 3.202 | 0.072 | 3.639 | 0.078 |
| TSEsimp-S without rec | 1.717 | 0.101 | 3.200 | 0.072 | 3.630 | 0.078 |
| TSEgest-C | -5.676 | 0.437 | 13.806 | 0.309 | 14.921 | 0.405 |
| TSEgest-S | -4.648 | 0.661 | 19.709 | 0.468 | 19.152 | 0.632 |
| TSEgest-C without rec | 0.560 | 0.102 | 3.211 | 0.072 | 3.258 | 0.070 |
| TSEgest-S without rec | 0.621 | 0.107 | 3.201 | 0.076 | 3.281 | 0.076 |
| IPCW-C unstabilised | -0.115 | 0.105 | 3.305 | 0.074 | 3.305 | 0.073 |
| IPCW-C stabilised | 0.878 | 0.104 | 3.276 | 0.073 | 3.390 | 0.076 |
| IPCW-S unstabilised | -0.119 | 0.106 | 3.316 | 0.075 | 3.305 | 0.074 |
| IPCW-S stabilised | 0.870 | 0.106 | 3.290 | 0.075 | 3.389 | 0.077 |

Table A2.18: Results for scenario 18

| Method & application | Bias in RMST | Bias  MCSE | EmpSE | EmpSE  MCSE | RMSE | RMSE  MCSE |
| --- | --- | --- | --- | --- | --- | --- |
| No switch | -0.179 | 0.101 | 3.183 | 0.071 | 3.187 | 0.068 |
| ITT | 4.908 | 0.101 | 3.189 | 0.071 | 5.852 | 0.092 |
| TSEsimp-C | -2.156 | 0.195 | 6.171 | 0.138 | 6.534 | 0.179 |
| TSEsimp-S | -2.952 | 0.410 | 12.950 | 0.290 | 13.370 | 0.490 |
| TSEsimp-C without rec | 1.288 | 0.104 | 3.292 | 0.074 | 3.534 | 0.075 |
| TSEsimp-S without rec | 1.281 | 0.104 | 3.297 | 0.074 | 3.535 | 0.075 |
| TSEgest-C | -4.938 | 0.341 | 10.781 | 0.241 | 11.853 | 0.315 |
| TSEgest-S | -3.285 | 0.830 | 23.725 | 0.587 | 21.787 | 0.762 |
| TSEgest-C without rec | 0.235 | 0.105 | 3.327 | 0.074 | 3.334 | 0.072 |
| TSEgest-S without rec | 0.319 | 0.119 | 3.398 | 0.084 | 3.377 | 0.081 |
| IPCW-C unstabilised | -0.249 | 0.108 | 3.400 | 0.076 | 3.407 | 0.074 |
| IPCW-C stabilised | 0.722 | 0.104 | 3.302 | 0.074 | 3.378 | 0.071 |
| IPCW-S unstabilised | -0.238 | 0.109 | 3.396 | 0.077 | 3.406 | 0.075 |
| IPCW-S stabilised | 0.733 | 0.106 | 3.296 | 0.075 | 3.376 | 0.072 |

Table A2.19: Results for scenario 19

| Method & application | Bias in RMST | Bias  MCSE | EmpSE | EmpSE  MCSE | RMSE | RMSE  MCSE |
| --- | --- | --- | --- | --- | --- | --- |
| No switch | 0.010 | 0.102 | 3.222 | 0.072 | 3.220 | 0.071 |
| ITT | 5.591 | 0.102 | 3.218 | 0.072 | 6.450 | 0.094 |
| TSEsimp-C | -2.323 | 0.218 | 6.899 | 0.154 | 7.276 | 0.202 |
| TSEsimp-S | -3.443 | 0.312 | 9.860 | 0.221 | 10.439 | 0.285 |
| TSEsimp-C without rec | 1.608 | 0.106 | 3.343 | 0.075 | 3.708 | 0.079 |
| TSEsimp-S without rec | 1.592 | 0.106 | 3.342 | 0.075 | 3.701 | 0.079 |
| TSEgest-C | -5.445 | 0.406 | 12.826 | 0.287 | 13.915 | 0.362 |
| TSEgest-S | -4.973 | 0.585 | 18.271 | 0.414 | 18.724 | 0.571 |
| TSEgest-C without rec | 0.501 | 0.106 | 3.357 | 0.075 | 3.394 | 0.075 |
| TSEgest-S without rec | 0.622 | 0.108 | 3.378 | 0.077 | 3.415 | 0.076 |
| IPCW-C unstabilised | -0.142 | 0.109 | 3.433 | 0.077 | 3.434 | 0.078 |
| IPCW-C stabilised | 0.859 | 0.106 | 3.354 | 0.075 | 3.461 | 0.076 |
| IPCW-S unstabilised | -0.143 | 0.109 | 3.431 | 0.077 | 3.432 | 0.078 |
| IPCW-S stabilised | 0.855 | 0.106 | 3.353 | 0.075 | 3.459 | 0.076 |

Table A2.20: Results for scenario 20

| Method & application | Bias in RMST | Bias  MCSE | EmpSE | EmpSE  MCSE | RMSE | RMSE  MCSE |
| --- | --- | --- | --- | --- | --- | --- |
| No switch | -0.003 | 0.100 | 3.166 | 0.071 | 3.165 | 0.072 |
| ITT | 5.520 | 0.099 | 3.122 | 0.070 | 6.341 | 0.092 |
| TSEsimp-C | -2.536 | 0.219 | 6.922 | 0.155 | 7.369 | 0.209 |
| TSEsimp-S | -3.624 | 0.331 | 10.480 | 0.234 | 11.084 | 0.288 |
| TSEsimp-C without rec | 1.553 | 0.105 | 3.314 | 0.074 | 3.658 | 0.079 |
| TSEsimp-S without rec | 1.548 | 0.105 | 3.315 | 0.074 | 3.657 | 0.079 |
| TSEgest-C | -5.680 | 0.411 | 12.988 | 0.291 | 14.163 | 0.337 |
| TSEgest-S | -5.567 | 0.751 | 23.073 | 0.532 | 23.081 | 0.753 |
| TSEgest-C without rec | 0.435 | 0.105 | 3.324 | 0.074 | 3.350 | 0.074 |
| TSEgest-S without rec | 0.491 | 0.110 | 3.366 | 0.078 | 3.379 | 0.077 |
| IPCW-C unstabilised | -0.144 | 0.109 | 3.462 | 0.077 | 3.464 | 0.077 |
| IPCW-C stabilised | 0.861 | 0.107 | 3.384 | 0.076 | 3.491 | 0.077 |
| IPCW-S unstabilised | -0.144 | 0.109 | 3.461 | 0.077 | 3.462 | 0.077 |
| IPCW-S stabilised | 0.857 | 0.107 | 3.384 | 0.076 | 3.489 | 0.077 |

Table A2.21: Results for scenario 21

| Method & application | Bias in RMST | Bias  MCSE | EmpSE | EmpSE  MCSE | RMSE | RMSE  MCSE |
| --- | --- | --- | --- | --- | --- | --- |
| No switch | 0.047 | 0.101 | 3.195 | 0.071 | 3.194 | 0.071 |
| ITT | 16.888 | 0.099 | 3.143 | 0.070 | 17.178 | 0.098 |
| TSEsimp-C | 3.861 | 0.225 | 7.119 | 0.159 | 8.095 | 0.175 |
| TSEsimp-S | 1.863 | 0.276 | 8.741 | 0.196 | 8.933 | 0.215 |
| TSEsimp-C without rec | 7.255 | 0.131 | 4.136 | 0.093 | 8.350 | 0.124 |
| TSEsimp-S without rec | 6.457 | 0.130 | 4.117 | 0.092 | 7.657 | 0.122 |
| TSEgest-C | -4.525 | 0.439 | 13.890 | 0.311 | 14.596 | 0.396 |
| TSEgest-S | -5.117 | 0.543 | 17.092 | 0.384 | 17.770 | 0.415 |
| TSEgest-C without rec | 2.704 | 0.136 | 4.298 | 0.096 | 5.078 | 0.112 |
| TSEgest-S without rec | 2.653 | 0.134 | 4.211 | 0.095 | 4.987 | 0.114 |
| IPCW-C unstabilised | 9.044 | 0.385 | 12.051 | 0.273 | 15.076 | 0.268 |
| IPCW-C stabilised | 6.534 | 0.304 | 9.503 | 0.215 | 11.530 | 0.244 |
| IPCW-S unstabilised | 9.247 | 0.451 | 11.955 | 0.319 | 15.546 | 0.319 |
| IPCW-S stabilised | 6.680 | 0.356 | 9.442 | 0.252 | 11.990 | 0.298 |

Table A2.22: Results for scenario 22

| Method & application | Bias in RMST | Bias  MCSE | EmpSE | EmpSE  MCSE | RMSE | RMSE  MCSE |
| --- | --- | --- | --- | --- | --- | --- |
| No switch | -0.016 | 0.097 | 3.072 | 0.069 | 3.070 | 0.066 |
| ITT | 14.504 | 0.098 | 3.107 | 0.070 | 14.833 | 0.096 |
| TSEsimp-C | 1.482 | 0.215 | 6.792 | 0.152 | 6.948 | 0.147 |
| TSEsimp-S | 1.537 | 0.303 | 9.586 | 0.214 | 9.704 | 0.246 |
| TSEsimp-C without rec | 4.940 | 0.123 | 3.888 | 0.087 | 6.285 | 0.107 |
| TSEsimp-S without rec | 5.371 | 0.126 | 3.996 | 0.089 | 6.693 | 0.112 |
| TSEgest-C | -6.299 | 0.404 | 12.754 | 0.286 | 14.199 | 0.369 |
| TSEgest-S | -4.819 | 0.560 | 17.604 | 0.396 | 18.137 | 0.485 |
| TSEgest-C without rec | 0.735 | 0.127 | 4.005 | 0.090 | 4.070 | 0.091 |
| TSEgest-S without rec | 1.456 | 0.134 | 4.218 | 0.095 | 4.446 | 0.107 |
| IPCW-C unstabilised | 8.589 | 0.382 | 11.864 | 0.270 | 14.718 | 0.256 |
| IPCW-C stabilised | 5.768 | 0.295 | 9.163 | 0.208 | 10.908 | 0.233 |
| IPCW-S unstabilised | 8.189 | 0.451 | 11.861 | 0.319 | 15.216 | 0.324 |
| IPCW-S stabilised | 5.642 | 0.345 | 9.073 | 0.244 | 11.337 | 0.279 |

Table A2.23: Results for scenario 23

| Method & application | Bias in RMST | Bias  MCSE | EmpSE | EmpSE  MCSE | RMSE | RMSE  MCSE |
| --- | --- | --- | --- | --- | --- | --- |
| No switch | 0.103 | 0.099 | 3.126 | 0.070 | 3.126 | 0.067 |
| ITT | 16.171 | 0.099 | 3.132 | 0.070 | 16.472 | 0.098 |
| TSEsimp-C | 3.487 | 0.221 | 6.973 | 0.156 | 7.793 | 0.189 |
| TSEsimp-S | 1.888 | 0.276 | 8.736 | 0.195 | 8.933 | 0.225 |
| TSEsimp-C without rec | 6.694 | 0.127 | 4.006 | 0.090 | 7.800 | 0.120 |
| TSEsimp-S without rec | 6.199 | 0.125 | 3.953 | 0.088 | 7.351 | 0.117 |
| TSEgest-C | -5.003 | 0.431 | 13.618 | 0.305 | 14.482 | 0.395 |
| TSEgest-S | -4.120 | 0.522 | 16.486 | 0.369 | 16.962 | 0.441 |
| TSEgest-C without rec | 2.224 | 0.135 | 4.268 | 0.096 | 4.810 | 0.110 |
| TSEgest-S without rec | 3.040 | 0.126 | 3.979 | 0.089 | 5.005 | 0.108 |
| IPCW-C unstabilised | 8.481 | 0.392 | 12.109 | 0.277 | 14.767 | 0.268 |
| IPCW-C stabilised | 6.020 | 0.301 | 9.305 | 0.213 | 11.060 | 0.234 |
| IPCW-S unstabilised | 8.080 | 0.416 | 12.011 | 0.295 | 14.785 | 0.291 |
| IPCW-S stabilised | 5.734 | 0.320 | 9.226 | 0.226 | 11.098 | 0.252 |

Table A2.24: Results for scenario 24

| Method & application | Bias in RMST | Bias  MCSE | EmpSE | EmpSE  MCSE | RMSE | RMSE  MCSE |
| --- | --- | --- | --- | --- | --- | --- |
| No switch | -0.068 | 0.099 | 3.137 | 0.070 | 3.136 | 0.071 |
| ITT | 15.811 | 0.099 | 3.138 | 0.070 | 16.119 | 0.099 |
| TSEsimp-C | 1.701 | 0.228 | 7.219 | 0.161 | 7.413 | 0.166 |
| TSEsimp-S | 2.118 | 0.307 | 9.713 | 0.217 | 9.936 | 0.258 |
| TSEsimp-C without rec | 5.451 | 0.124 | 3.919 | 0.088 | 6.713 | 0.116 |
| TSEsimp-S without rec | 5.861 | 0.126 | 3.979 | 0.089 | 7.083 | 0.118 |
| TSEgest-C | -6.883 | 0.466 | 14.735 | 0.330 | 16.249 | 0.407 |
| TSEgest-S | -3.984 | 0.533 | 16.836 | 0.377 | 17.279 | 0.401 |
| TSEgest-C without rec | 1.167 | 0.129 | 4.092 | 0.092 | 4.251 | 0.112 |
| TSEgest-S without rec | 2.535 | 0.126 | 3.977 | 0.089 | 4.721 | 0.110 |
| IPCW-C unstabilised | 8.219 | 0.380 | 11.771 | 0.269 | 14.366 | 0.267 |
| IPCW-C stabilised | 5.520 | 0.292 | 9.054 | 0.207 | 10.632 | 0.235 |
| IPCW-S unstabilised | 7.960 | 0.414 | 11.836 | 0.293 | 14.497 | 0.293 |
| IPCW-S stabilised | 5.387 | 0.317 | 9.064 | 0.224 | 10.758 | 0.258 |

Table A2.25: Results for scenario 25

| Method & application | Bias in RMST | Bias  MCSE | EmpSE | EmpSE  MCSE | RMSE | RMSE  MCSE |
| --- | --- | --- | --- | --- | --- | --- |
| No switch | -0.017 | 0.070 | 2.214 | 0.050 | 2.213 | 0.048 |
| ITT | 5.891 | 0.070 | 2.227 | 0.050 | 6.298 | 0.069 |
| TSEsimp-C | -2.898 | 0.163 | 5.142 | 0.115 | 5.900 | 0.157 |
| TSEsimp-S | -3.224 | 0.186 | 5.896 | 0.132 | 6.718 | 0.165 |
| TSEsimp-C without rec | 1.707 | 0.073 | 2.302 | 0.051 | 2.865 | 0.061 |
| TSEsimp-S without rec | 1.696 | 0.073 | 2.299 | 0.051 | 2.856 | 0.061 |
| TSEgest-C | -6.650 | 0.306 | 9.662 | 0.216 | 11.726 | 0.255 |
| TSEgest-S | -6.936 | 0.492 | 15.388 | 0.348 | 16.864 | 0.663 |
| TSEgest-C without rec | 0.527 | 0.073 | 2.318 | 0.052 | 2.376 | 0.052 |
| TSEgest-S without rec | 0.574 | 0.074 | 2.305 | 0.052 | 2.393 | 0.053 |
| IPCW-C unstabilised | -0.128 | 0.075 | 2.371 | 0.053 | 2.373 | 0.052 |
| IPCW-C stabilised | 0.820 | 0.073 | 2.318 | 0.052 | 2.458 | 0.054 |
| IPCW-S unstabilised | -0.129 | 0.075 | 2.371 | 0.053 | 2.373 | 0.052 |
| IPCW-S stabilised | 0.818 | 0.073 | 2.319 | 0.052 | 2.458 | 0.055 |

Table A2.26: Results for scenario 26

| Method & application | Bias in RMST | Bias  MCSE | EmpSE | EmpSE  MCSE | RMSE | RMSE  MCSE |
| --- | --- | --- | --- | --- | --- | --- |
| No switch | -0.046 | 0.072 | 2.272 | 0.051 | 2.271 | 0.051 |
| ITT | 5.089 | 0.071 | 2.252 | 0.050 | 5.564 | 0.068 |
| TSEsimp-C | -2.492 | 0.141 | 4.469 | 0.100 | 5.115 | 0.134 |
| TSEsimp-S | -3.922 | 0.239 | 7.571 | 0.169 | 8.524 | 0.245 |
| TSEsimp-C without rec | 1.374 | 0.073 | 2.313 | 0.052 | 2.689 | 0.059 |
| TSEsimp-S without rec | 1.373 | 0.073 | 2.314 | 0.052 | 2.689 | 0.059 |
| TSEgest-C | -5.774 | 0.250 | 7.898 | 0.177 | 9.780 | 0.221 |
| TSEgest-S | -6.139 | 0.600 | 18.453 | 0.424 | 19.087 | 0.808 |
| TSEgest-C without rec | 0.291 | 0.074 | 2.326 | 0.052 | 2.343 | 0.055 |
| TSEgest-S without rec | 0.317 | 0.076 | 2.331 | 0.054 | 2.359 | 0.056 |
| IPCW-C unstabilised | -0.246 | 0.075 | 2.377 | 0.053 | 2.389 | 0.055 |
| IPCW-C stabilised | 0.758 | 0.075 | 2.361 | 0.053 | 2.478 | 0.058 |
| IPCW-S unstabilised | -0.247 | 0.075 | 2.377 | 0.053 | 2.388 | 0.055 |
| IPCW-S stabilised | 0.756 | 0.075 | 2.360 | 0.053 | 2.477 | 0.058 |

Table A2.27: Results for scenario 27

| Method & application | Bias in RMST | Bias  MCSE | EmpSE | EmpSE  MCSE | RMSE | RMSE  MCSE |
| --- | --- | --- | --- | --- | --- | --- |
| No switch | -0.064 | 0.069 | 2.183 | 0.049 | 2.183 | 0.047 |
| ITT | 5.556 | 0.069 | 2.191 | 0.049 | 5.972 | 0.067 |
| TSEsimp-C | -2.475 | 0.149 | 4.698 | 0.105 | 5.308 | 0.142 |
| TSEsimp-S | -3.327 | 0.192 | 6.077 | 0.136 | 6.925 | 0.179 |
| TSEsimp-C without rec | 1.570 | 0.072 | 2.264 | 0.051 | 2.754 | 0.059 |
| TSEsimp-S without rec | 1.562 | 0.072 | 2.263 | 0.051 | 2.748 | 0.059 |
| TSEgest-C | -6.609 | 0.270 | 8.530 | 0.191 | 10.788 | 0.240 |
| TSEgest-S | -6.716 | 0.367 | 11.611 | 0.260 | 13.402 | 0.351 |
| TSEgest-C without rec | 0.426 | 0.072 | 2.277 | 0.051 | 2.315 | 0.051 |
| TSEgest-S without rec | 0.568 | 0.072 | 2.278 | 0.051 | 2.346 | 0.052 |
| IPCW-C unstabilised | -0.225 | 0.074 | 2.333 | 0.052 | 2.343 | 0.051 |
| IPCW-C stabilised | 0.757 | 0.073 | 2.299 | 0.051 | 2.419 | 0.054 |
| IPCW-S unstabilised | -0.226 | 0.074 | 2.334 | 0.052 | 2.344 | 0.051 |
| IPCW-S stabilised | 0.754 | 0.073 | 2.299 | 0.051 | 2.419 | 0.054 |

Table A2.28: Results for scenario 28

| Method & application | Bias in RMST | Bias  MCSE | EmpSE | EmpSE  MCSE | RMSE | RMSE  MCSE |
| --- | --- | --- | --- | --- | --- | --- |
| No switch | -0.064 | 0.070 | 2.215 | 0.050 | 2.215 | 0.049 |
| ITT | 5.495 | 0.070 | 2.210 | 0.049 | 5.922 | 0.066 |
| TSEsimp-C | -2.649 | 0.151 | 4.773 | 0.107 | 5.457 | 0.144 |
| TSEsimp-S | -3.299 | 0.201 | 6.368 | 0.142 | 7.169 | 0.180 |
| TSEsimp-C without rec | 1.526 | 0.072 | 2.279 | 0.051 | 2.742 | 0.058 |
| TSEsimp-S without rec | 1.524 | 0.072 | 2.279 | 0.051 | 2.741 | 0.058 |
| TSEgest-C | -6.202 | 0.265 | 8.391 | 0.188 | 10.431 | 0.246 |
| TSEgest-S | -6.307 | 0.466 | 14.726 | 0.330 | 16.042 | 0.521 |
| TSEgest-C without rec | 0.380 | 0.072 | 2.292 | 0.051 | 2.322 | 0.052 |
| TSEgest-S without rec | 0.492 | 0.073 | 2.301 | 0.052 | 2.349 | 0.052 |
| IPCW-C unstabilised | -0.214 | 0.075 | 2.359 | 0.053 | 2.367 | 0.052 |
| IPCW-C stabilised | 0.755 | 0.073 | 2.313 | 0.052 | 2.432 | 0.053 |
| IPCW-S unstabilised | -0.215 | 0.075 | 2.359 | 0.053 | 2.367 | 0.052 |
| IPCW-S stabilised | 0.753 | 0.073 | 2.314 | 0.052 | 2.432 | 0.053 |

Table A2.29: Results for scenario 29

| Method & application | Bias in RMST | Bias  MCSE | EmpSE | EmpSE  MCSE | RMSE | RMSE  MCSE |
| --- | --- | --- | --- | --- | --- | --- |
| No switch | -0.048 | 0.071 | 2.243 | 0.050 | 2.242 | 0.050 |
| ITT | 16.719 | 0.070 | 2.209 | 0.049 | 16.864 | 0.069 |
| TSEsimp-C | 3.395 | 0.154 | 4.874 | 0.109 | 5.938 | 0.105 |
| TSEsimp-S | 1.477 | 0.192 | 6.059 | 0.136 | 6.234 | 0.135 |
| TSEsimp-C without rec | 6.997 | 0.088 | 2.779 | 0.062 | 7.528 | 0.084 |
| TSEsimp-S without rec | 6.208 | 0.088 | 2.780 | 0.062 | 6.801 | 0.084 |
| TSEgest-C | -5.489 | 0.323 | 10.200 | 0.229 | 11.553 | 0.289 |
| TSEgest-S | -5.974 | 0.376 | 11.883 | 0.266 | 13.295 | 0.306 |
| TSEgest-C without rec | 2.388 | 0.092 | 2.915 | 0.065 | 3.768 | 0.076 |
| TSEgest-S without rec | 2.283 | 0.090 | 2.831 | 0.063 | 3.636 | 0.074 |
| IPCW-C unstabilised | 5.922 | 0.317 | 9.921 | 0.225 | 11.630 | 0.225 |
| IPCW-C stabilised | 3.838 | 0.217 | 6.794 | 0.154 | 7.855 | 0.179 |
| IPCW-S unstabilised | 5.867 | 0.360 | 10.149 | 0.254 | 12.275 | 0.287 |
| IPCW-S stabilised | 3.869 | 0.246 | 6.939 | 0.174 | 8.508 | 0.232 |

Table A2.30: Results for scenario 30

| Method & application | Bias in RMST | Bias  MCSE | EmpSE | EmpSE  MCSE | RMSE | RMSE  MCSE |
| --- | --- | --- | --- | --- | --- | --- |
| No switch | -0.145 | 0.069 | 2.168 | 0.049 | 2.172 | 0.051 |
| ITT | 14.435 | 0.069 | 2.182 | 0.049 | 14.598 | 0.068 |
| TSEsimp-C | 1.123 | 0.152 | 4.818 | 0.108 | 4.945 | 0.106 |
| TSEsimp-S | 0.968 | 0.193 | 6.107 | 0.137 | 6.180 | 0.144 |
| TSEsimp-C without rec | 4.739 | 0.086 | 2.728 | 0.061 | 5.468 | 0.079 |
| TSEsimp-S without rec | 5.173 | 0.088 | 2.786 | 0.062 | 5.875 | 0.082 |
| TSEgest-C | -7.284 | 0.294 | 9.294 | 0.208 | 11.785 | 0.292 |
| TSEgest-S | -6.772 | 0.362 | 11.442 | 0.256 | 13.291 | 0.395 |
| TSEgest-C without rec | 0.619 | 0.089 | 2.803 | 0.063 | 2.880 | 0.065 |
| TSEgest-S without rec | 1.215 | 0.091 | 2.880 | 0.064 | 3.124 | 0.071 |
| IPCW-C unstabilised | 4.943 | 0.313 | 9.790 | 0.221 | 10.998 | 0.227 |
| IPCW-C stabilised | 3.285 | 0.220 | 6.886 | 0.156 | 7.626 | 0.187 |
| IPCW-S unstabilised | 4.653 | 0.340 | 9.672 | 0.241 | 11.502 | 0.270 |
| IPCW-S stabilised | 3.186 | 0.238 | 6.783 | 0.169 | 8.284 | 0.237 |

Table A2.31: Results for scenario 31

| Method & application | Bias in RMST | Bias  MCSE | EmpSE | EmpSE  MCSE | RMSE | RMSE  MCSE |
| --- | --- | --- | --- | --- | --- | --- |
| No switch | -0.133 | 0.071 | 2.241 | 0.050 | 2.244 | 0.049 |
| ITT | 15.916 | 0.069 | 2.192 | 0.049 | 16.066 | 0.069 |
| TSEsimp-C | 2.924 | 0.148 | 4.678 | 0.105 | 5.515 | 0.101 |
| TSEsimp-S | 1.582 | 0.178 | 5.622 | 0.126 | 5.838 | 0.131 |
| TSEsimp-C without rec | 6.374 | 0.089 | 2.830 | 0.063 | 6.974 | 0.084 |
| TSEsimp-S without rec | 5.867 | 0.089 | 2.802 | 0.063 | 6.502 | 0.082 |
| TSEgest-C | -5.942 | 0.299 | 9.437 | 0.212 | 11.119 | 0.280 |
| TSEgest-S | -5.403 | 0.355 | 11.219 | 0.251 | 12.447 | 0.297 |
| TSEgest-C without rec | 1.896 | 0.092 | 2.915 | 0.065 | 3.481 | 0.077 |
| TSEgest-S without rec | 2.721 | 0.088 | 2.784 | 0.062 | 3.892 | 0.078 |
| IPCW-C unstabilised | 5.473 | 0.319 | 9.963 | 0.225 | 11.434 | 0.235 |
| IPCW-C stabilised | 3.686 | 0.224 | 6.992 | 0.158 | 7.949 | 0.188 |
| IPCW-S unstabilised | 5.446 | 0.320 | 9.918 | 0.227 | 11.371 | 0.237 |
| IPCW-S stabilised | 3.667 | 0.224 | 6.936 | 0.159 | 7.903 | 0.190 |

Table A2.32: Results for scenario 32

| Method & application | Bias in RMST | Bias  MCSE | EmpSE | EmpSE  MCSE | RMSE | RMSE  MCSE |
| --- | --- | --- | --- | --- | --- | --- |
| No switch | -0.078 | 0.068 | 2.158 | 0.048 | 2.158 | 0.047 |
| ITT | 15.708 | 0.067 | 2.104 | 0.047 | 15.848 | 0.066 |
| TSEsimp-C | 1.550 | 0.154 | 4.859 | 0.109 | 5.098 | 0.108 |
| TSEsimp-S | 1.039 | 0.197 | 6.226 | 0.139 | 6.309 | 0.149 |
| TSEsimp-C without rec | 5.473 | 0.085 | 2.689 | 0.060 | 6.097 | 0.081 |
| TSEsimp-S without rec | 5.923 | 0.086 | 2.732 | 0.061 | 6.522 | 0.082 |
| TSEgest-C | -7.239 | 0.312 | 9.832 | 0.221 | 12.167 | 0.278 |
| TSEgest-S | -5.455 | 0.350 | 11.064 | 0.248 | 12.331 | 0.279 |
| TSEgest-C without rec | 1.193 | 0.090 | 2.836 | 0.064 | 3.081 | 0.070 |
| TSEgest-S without rec | 2.557 | 0.089 | 2.805 | 0.063 | 3.794 | 0.076 |
| IPCW-C unstabilised | 5.912 | 0.320 | 10.007 | 0.227 | 11.688 | 0.230 |
| IPCW-C stabilised | 3.948 | 0.226 | 7.052 | 0.160 | 8.106 | 0.181 |
| IPCW-S unstabilised | 5.812 | 0.325 | 10.021 | 0.230 | 11.817 | 0.246 |
| IPCW-S stabilised | 3.870 | 0.229 | 7.045 | 0.162 | 8.180 | 0.189 |

Table A2.33: Results for scenario 33

| Method & application | Bias in RMST | Bias  MCSE | EmpSE | EmpSE  MCSE | RMSE | RMSE  MCSE |
| --- | --- | --- | --- | --- | --- | --- |
| No switch | -0.006 | 0.046 | 1.469 | 0.033 | 1.468 | 0.037 |
| ITT | 2.406 | 0.045 | 1.419 | 0.032 | 2.793 | 0.042 |
| TSEsimp-C | -2.286 | 0.193 | 6.107 | 0.137 | 6.518 | 0.244 |
| TSEsimp-S | -2.586 | 0.268 | 8.487 | 0.190 | 8.868 | 0.414 |
| TSEsimp-C without rec | 0.869 | 0.047 | 1.496 | 0.033 | 1.730 | 0.039 |
| TSEsimp-S without rec | 0.864 | 0.047 | 1.496 | 0.033 | 1.727 | 0.039 |
| TSEgest-C | -4.189 | 0.386 | 12.202 | 0.273 | 12.878 | 0.473 |
| TSEgest-S | -4.553 | 0.621 | 17.681 | 0.439 | 16.485 | 0.890 |
| TSEgest-C without rec | 0.374 | 0.048 | 1.513 | 0.034 | 1.567 | 0.037 |
| TSEgest-S without rec | 0.354 | 0.053 | 1.519 | 0.038 | 1.575 | 0.041 |
| IPCW-C unstabilised | -0.110 | 0.050 | 1.596 | 0.036 | 1.599 | 0.040 |
| IPCW-C stabilised | 0.231 | 0.049 | 1.546 | 0.035 | 1.562 | 0.038 |
| IPCW-S unstabilised | -0.109 | 0.050 | 1.593 | 0.036 | 1.600 | 0.040 |
| IPCW-S stabilised | 0.231 | 0.049 | 1.545 | 0.035 | 1.562 | 0.038 |

Table A2.34: Results for scenario 34

| Method & application | Bias in RMST | Bias  MCSE | EmpSE | EmpSE  MCSE | RMSE | RMSE  MCSE |
| --- | --- | --- | --- | --- | --- | --- |
| No switch | -0.031 | 0.047 | 1.481 | 0.033 | 1.480 | 0.034 |
| ITT | 2.092 | 0.046 | 1.445 | 0.032 | 2.542 | 0.041 |
| TSEsimp-C | -2.035 | 0.167 | 5.289 | 0.118 | 5.664 | 0.284 |
| TSEsimp-S | -2.944 | 0.342 | 10.819 | 0.242 | 11.207 | 0.614 |
| TSEsimp-C without rec | 0.675 | 0.048 | 1.511 | 0.034 | 1.655 | 0.037 |
| TSEsimp-S without rec | 0.668 | 0.048 | 1.512 | 0.034 | 1.653 | 0.037 |
| TSEgest-C | -3.916 | 0.330 | 10.421 | 0.233 | 11.122 | 0.434 |
| TSEgest-S | -2.985 | 0.648 | 16.933 | 0.459 | 14.251 | 1.025 |
| TSEgest-C without rec | 0.228 | 0.049 | 1.553 | 0.035 | 1.568 | 0.036 |
| TSEgest-S without rec | 0.228 | 0.059 | 1.539 | 0.042 | 1.569 | 0.044 |
| IPCW-C unstabilised | -0.214 | 0.051 | 1.621 | 0.036 | 1.634 | 0.038 |
| IPCW-C stabilised | 0.143 | 0.050 | 1.590 | 0.036 | 1.596 | 0.037 |
| IPCW-S unstabilised | -0.217 | 0.051 | 1.621 | 0.036 | 1.635 | 0.038 |
| IPCW-S stabilised | 0.139 | 0.050 | 1.590 | 0.036 | 1.596 | 0.037 |

Table A2.35: Results for scenario 35

| Method & application | Bias in RMST | Bias  MCSE | EmpSE | EmpSE  MCSE | RMSE | RMSE  MCSE |
| --- | --- | --- | --- | --- | --- | --- |
| No switch | -0.018 | 0.047 | 1.491 | 0.033 | 1.490 | 0.032 |
| ITT | 2.303 | 0.045 | 1.438 | 0.032 | 2.715 | 0.042 |
| TSEsimp-C | -1.631 | 0.170 | 5.361 | 0.120 | 5.601 | 0.229 |
| TSEsimp-S | -2.017 | 0.233 | 7.370 | 0.165 | 7.637 | 0.295 |
| TSEsimp-C without rec | 0.802 | 0.048 | 1.530 | 0.034 | 1.727 | 0.037 |
| TSEsimp-S without rec | 0.798 | 0.048 | 1.529 | 0.034 | 1.724 | 0.037 |
| TSEgest-C | -3.911 | 0.363 | 11.456 | 0.257 | 12.089 | 0.460 |
| TSEgest-S | -4.191 | 0.530 | 16.199 | 0.375 | 16.185 | 0.702 |
| TSEgest-C without rec | 0.329 | 0.049 | 1.561 | 0.035 | 1.601 | 0.035 |
| TSEgest-S without rec | 0.319 | 0.051 | 1.572 | 0.036 | 1.609 | 0.037 |
| IPCW-C unstabilised | -0.174 | 0.052 | 1.643 | 0.037 | 1.652 | 0.036 |
| IPCW-C stabilised | 0.181 | 0.051 | 1.601 | 0.036 | 1.611 | 0.036 |
| IPCW-S unstabilised | -0.178 | 0.052 | 1.644 | 0.037 | 1.652 | 0.036 |
| IPCW-S stabilised | 0.177 | 0.051 | 1.602 | 0.036 | 1.611 | 0.036 |

Table A2.36: Results for scenario 36

| Method & application | Bias in RMST | Bias  MCSE | EmpSE | EmpSE  MCSE | RMSE | RMSE  MCSE |
| --- | --- | --- | --- | --- | --- | --- |
| No switch | -0.111 | 0.049 | 1.536 | 0.034 | 1.539 | 0.038 |
| ITT | 2.180 | 0.048 | 1.504 | 0.034 | 2.648 | 0.044 |
| TSEsimp-C | -1.977 | 0.166 | 5.255 | 0.118 | 5.612 | 0.214 |
| TSEsimp-S | -2.819 | 0.277 | 8.761 | 0.196 | 9.199 | 0.352 |
| TSEsimp-C without rec | 0.689 | 0.050 | 1.570 | 0.035 | 1.713 | 0.041 |
| TSEsimp-S without rec | 0.683 | 0.050 | 1.570 | 0.035 | 1.712 | 0.041 |
| TSEgest-C | -3.755 | 0.334 | 10.549 | 0.236 | 11.170 | 0.397 |
| TSEgest-S | -4.131 | 0.600 | 17.461 | 0.425 | 16.532 | 0.855 |
| TSEgest-C without rec | 0.229 | 0.051 | 1.604 | 0.036 | 1.618 | 0.041 |
| TSEgest-S without rec | 0.229 | 0.055 | 1.600 | 0.039 | 1.625 | 0.045 |
| IPCW-C unstabilised | -0.250 | 0.053 | 1.666 | 0.037 | 1.684 | 0.043 |
| IPCW-C stabilised | 0.121 | 0.052 | 1.629 | 0.036 | 1.633 | 0.041 |
| IPCW-S unstabilised | -0.254 | 0.053 | 1.667 | 0.037 | 1.685 | 0.043 |
| IPCW-S stabilised | 0.117 | 0.052 | 1.629 | 0.036 | 1.633 | 0.041 |

Table A2.37: Results for scenario 37

| Method & application | Bias in RMST | Bias  MCSE | EmpSE | EmpSE  MCSE | RMSE | RMSE  MCSE |
| --- | --- | --- | --- | --- | --- | --- |
| No switch | -0.039 | 0.047 | 1.494 | 0.033 | 1.494 | 0.035 |
| ITT | 7.222 | 0.042 | 1.319 | 0.030 | 7.341 | 0.041 |
| TSEsimp-C | 1.378 | 0.150 | 4.752 | 0.106 | 4.945 | 0.178 |
| TSEsimp-S | 0.090 | 0.210 | 6.652 | 0.149 | 6.650 | 0.249 |
| TSEsimp-C without rec | 3.488 | 0.054 | 1.710 | 0.038 | 3.884 | 0.051 |
| TSEsimp-S without rec | 3.162 | 0.054 | 1.709 | 0.038 | 3.594 | 0.051 |
| TSEgest-C | -3.203 | 0.375 | 11.837 | 0.265 | 12.233 | 0.475 |
| TSEgest-S | -3.198 | 0.438 | 13.554 | 0.310 | 13.639 | 0.538 |
| TSEgest-C without rec | 1.728 | 0.058 | 1.839 | 0.041 | 2.520 | 0.052 |
| TSEgest-S without rec | 1.729 | 0.058 | 1.786 | 0.041 | 2.480 | 0.051 |
| IPCW-C unstabilised | 1.221 | 0.158 | 4.958 | 0.112 | 5.117 | 0.094 |
| IPCW-C stabilised | 1.075 | 0.114 | 3.569 | 0.081 | 3.738 | 0.078 |
| IPCW-S unstabilised | 1.279 | 0.175 | 4.936 | 0.124 | 5.691 | 0.222 |
| IPCW-S stabilised | 1.149 | 0.126 | 3.541 | 0.089 | 4.238 | 0.212 |

Table A2.38: Results for scenario 38

| Method & application | Bias in RMST | Bias  MCSE | EmpSE | EmpSE  MCSE | RMSE | RMSE  MCSE |
| --- | --- | --- | --- | --- | --- | --- |
| No switch | -0.112 | 0.048 | 1.506 | 0.034 | 1.510 | 0.033 |
| ITT | 6.367 | 0.042 | 1.326 | 0.030 | 6.504 | 0.042 |
| TSEsimp-C | 0.142 | 0.143 | 4.521 | 0.101 | 4.521 | 0.148 |
| TSEsimp-S | -0.282 | 0.237 | 7.491 | 0.168 | 7.492 | 0.305 |
| TSEsimp-C without rec | 2.491 | 0.055 | 1.733 | 0.039 | 3.034 | 0.051 |
| TSEsimp-S without rec | 2.634 | 0.055 | 1.752 | 0.039 | 3.163 | 0.051 |
| TSEgest-C | -4.050 | 0.317 | 10.016 | 0.224 | 10.784 | 0.370 |
| TSEgest-S | -4.885 | 0.506 | 15.354 | 0.358 | 15.472 | 0.699 |
| TSEgest-C without rec | 0.792 | 0.058 | 1.841 | 0.041 | 2.006 | 0.045 |
| TSEgest-S without rec | 0.928 | 0.060 | 1.833 | 0.043 | 2.102 | 0.051 |
| IPCW-C unstabilised | 1.118 | 0.164 | 5.097 | 0.116 | 5.224 | 0.098 |
| IPCW-C stabilised | 0.933 | 0.122 | 3.810 | 0.087 | 3.929 | 0.085 |
| IPCW-S unstabilised | 1.193 | 0.185 | 5.104 | 0.131 | 6.426 | 0.394 |
| IPCW-S stabilised | 1.014 | 0.137 | 3.788 | 0.097 | 4.697 | 0.277 |

Table A2.39: Results for scenario 39

| Method & application | Bias in RMST | Bias  MCSE | EmpSE | EmpSE  MCSE | RMSE | RMSE  MCSE |
| --- | --- | --- | --- | --- | --- | --- |
| No switch | -0.044 | 0.049 | 1.546 | 0.035 | 1.546 | 0.034 |
| ITT | 6.930 | 0.043 | 1.352 | 0.030 | 7.061 | 0.042 |
| TSEsimp-C | 0.838 | 0.152 | 4.793 | 0.107 | 4.863 | 0.191 |
| TSEsimp-S | 0.346 | 0.190 | 5.999 | 0.134 | 6.006 | 0.191 |
| TSEsimp-C without rec | 3.228 | 0.055 | 1.731 | 0.039 | 3.662 | 0.050 |
| TSEsimp-S without rec | 3.031 | 0.055 | 1.727 | 0.039 | 3.488 | 0.050 |
| TSEgest-C | -3.002 | 0.333 | 10.522 | 0.236 | 10.916 | 0.439 |
| TSEgest-S | -2.388 | 0.382 | 12.056 | 0.270 | 12.268 | 0.487 |
| TSEgest-C without rec | 1.436 | 0.060 | 1.884 | 0.042 | 2.368 | 0.051 |
| TSEgest-S without rec | 1.755 | 0.057 | 1.793 | 0.040 | 2.512 | 0.050 |
| IPCW-C unstabilised | 1.515 | 0.168 | 5.243 | 0.119 | 5.487 | 0.094 |
| IPCW-C stabilised | 1.215 | 0.123 | 3.833 | 0.087 | 4.030 | 0.082 |
| IPCW-S unstabilised | 1.443 | 0.171 | 5.279 | 0.121 | 5.601 | 0.156 |
| IPCW-S stabilised | 1.203 | 0.124 | 3.834 | 0.088 | 4.099 | 0.111 |

Table A2.40: Results for scenario 40

| Method & application | Bias in RMST | Bias  MCSE | EmpSE | EmpSE  MCSE | RMSE | RMSE  MCSE |
| --- | --- | --- | --- | --- | --- | --- |
| No switch | -0.035 | 0.047 | 1.498 | 0.034 | 1.497 | 0.034 |
| ITT | 6.788 | 0.042 | 1.342 | 0.030 | 6.919 | 0.042 |
| TSEsimp-C | -0.185 | 0.170 | 5.369 | 0.120 | 5.370 | 0.240 |
| TSEsimp-S | -0.008 | 0.231 | 7.321 | 0.164 | 7.317 | 0.275 |
| TSEsimp-C without rec | 2.687 | 0.055 | 1.725 | 0.039 | 3.193 | 0.049 |
| TSEsimp-S without rec | 2.838 | 0.055 | 1.746 | 0.039 | 3.331 | 0.050 |
| TSEgest-C | -4.520 | 0.369 | 11.652 | 0.261 | 12.463 | 0.444 |
| TSEgest-S | -3.538 | 0.455 | 14.382 | 0.322 | 14.804 | 0.582 |
| TSEgest-C without rec | 1.048 | 0.058 | 1.822 | 0.041 | 2.104 | 0.045 |
| TSEgest-S without rec | 1.472 | 0.057 | 1.795 | 0.040 | 2.321 | 0.046 |
| IPCW-C unstabilised | 1.068 | 0.164 | 5.126 | 0.116 | 5.238 | 0.099 |
| IPCW-C stabilised | 0.890 | 0.122 | 3.820 | 0.087 | 3.915 | 0.081 |
| IPCW-S unstabilised | 1.114 | 0.166 | 5.120 | 0.117 | 5.269 | 0.102 |
| IPCW-S stabilised | 0.906 | 0.123 | 3.800 | 0.087 | 3.927 | 0.085 |

Table A2.41: Results for scenario 41

| Method & application | Bias in RMST | Bias  MCSE | EmpSE | EmpSE  MCSE | RMSE | RMSE  MCSE |
| --- | --- | --- | --- | --- | --- | --- |
| No switch | 0.040 | 0.117 | 3.710 | 0.083 | 3.709 | 0.086 |
| ITT | 9.522 | 0.125 | 3.944 | 0.088 | 10.306 | 0.124 |
| TSEsimp-C | -2.481 | 0.218 | 6.882 | 0.154 | 7.312 | 0.169 |
| TSEsimp-S | -3.508 | 0.485 | 15.336 | 0.343 | 15.724 | 0.551 |
| TSEsimp-C without rec | 2.145 | 0.123 | 3.894 | 0.087 | 4.444 | 0.104 |
| TSEsimp-S without rec | 2.105 | 0.123 | 3.891 | 0.087 | 4.422 | 0.103 |
| TSEgest-C | -6.298 | 0.369 | 11.649 | 0.261 | 13.237 | 0.301 |
| TSEgest-S | 1.181 | 1.135 | 34.636 | 0.803 | 33.577 | 1.091 |
| TSEgest-C without rec | 0.553 | 0.123 | 3.886 | 0.087 | 3.923 | 0.091 |
| TSEgest-S without rec | 7.474 | 0.130 | 3.971 | 0.092 | 8.533 | 0.128 |
| IPCW-C unstabilised | -0.069 | 0.122 | 3.856 | 0.086 | 3.855 | 0.086 |
| IPCW-C stabilised | 1.500 | 0.124 | 3.915 | 0.088 | 4.191 | 0.103 |
| IPCW-S unstabilised | -0.252 | 0.136 | 3.835 | 0.096 | 3.859 | 0.096 |
| IPCW-S stabilised | 1.343 | 0.137 | 3.864 | 0.097 | 4.187 | 0.115 |

Table A2.42: Results for scenario 42

| Method & application | Bias in RMST | Bias  MCSE | EmpSE | EmpSE  MCSE | RMSE | RMSE  MCSE |
| --- | --- | --- | --- | --- | --- | --- |
| No switch | 0.278 | 0.121 | 3.836 | 0.086 | 3.844 | 0.089 |
| ITT | 27.088 | 0.138 | 4.357 | 0.097 | 27.436 | 0.136 |
| TSEsimp-C | 5.293 | 0.244 | 7.704 | 0.172 | 9.344 | 0.188 |
| TSEsimp-S | 2.038 | 0.375 | 11.859 | 0.265 | 12.027 | 0.349 |
| TSEsimp-C without rec | 9.283 | 0.167 | 5.266 | 0.118 | 10.671 | 0.161 |
| TSEsimp-S without rec | 7.973 | 0.164 | 5.190 | 0.116 | 9.512 | 0.157 |
| TSEgest-C | -5.000 | 0.378 | 11.917 | 0.267 | 12.903 | 0.316 |
| TSEgest-S | -2.632 | 0.520 | 16.381 | 0.368 | 16.533 | 0.499 |
| TSEgest-C without rec | 2.847 | 0.164 | 5.166 | 0.116 | 5.908 | 0.138 |
| TSEgest-S without rec | 21.520 | 0.142 | 4.459 | 0.100 | 22.003 | 0.139 |
| IPCW-C unstabilised | 15.233 | 0.597 | 18.568 | 0.422 | 24.159 | 0.502 |
| IPCW-C stabilised | 10.133 | 0.435 | 13.526 | 0.307 | 16.997 | 0.422 |
| IPCW-S unstabilised | 14.253 | 1.263 | 18.349 | 0.895 | 24.806 | 1.066 |
| IPCW-S stabilised | 10.103 | 0.880 | 12.777 | 0.623 | 17.773 | 0.995 |

Table A2.43: Results for scenario 43

| Method & application | Bias in RMST | Bias  MCSE | EmpSE | EmpSE  MCSE | RMSE | RMSE  MCSE |
| --- | --- | --- | --- | --- | --- | --- |
| No switch | 0.121 | 0.087 | 2.738 | 0.061 | 2.739 | 0.062 |
| ITT | 9.705 | 0.092 | 2.903 | 0.065 | 10.130 | 0.092 |
| TSEsimp-C | -2.304 | 0.156 | 4.932 | 0.110 | 5.441 | 0.118 |
| TSEsimp-S | -4.962 | 0.247 | 7.801 | 0.175 | 9.243 | 0.215 |
| TSEsimp-C without rec | 2.274 | 0.091 | 2.878 | 0.064 | 3.667 | 0.080 |
| TSEsimp-S without rec | 2.243 | 0.091 | 2.879 | 0.064 | 3.648 | 0.080 |
| TSEgest-C | -7.385 | 0.248 | 7.844 | 0.175 | 10.771 | 0.206 |
| TSEgest-S | -7.540 | 0.624 | 19.700 | 0.441 | 21.064 | 0.987 |
| TSEgest-C without rec | 0.686 | 0.090 | 2.833 | 0.063 | 2.914 | 0.067 |
| TSEgest-S without rec | 7.698 | 0.093 | 2.924 | 0.065 | 8.234 | 0.092 |
| IPCW-C unstabilised | 0.040 | 0.090 | 2.837 | 0.063 | 2.836 | 0.063 |
| IPCW-C stabilised | 1.631 | 0.091 | 2.893 | 0.065 | 3.320 | 0.078 |
| IPCW-S unstabilised | 0.036 | 0.090 | 2.832 | 0.063 | 2.835 | 0.064 |
| IPCW-S stabilised | 1.624 | 0.092 | 2.892 | 0.065 | 3.317 | 0.078 |

Table A2.44: Results for scenario 44

| Method & application | Bias in RMST | Bias  MCSE | EmpSE | EmpSE  MCSE | RMSE | RMSE  MCSE |
| --- | --- | --- | --- | --- | --- | --- |
| No switch | 0.060 | 0.084 | 2.665 | 0.060 | 2.664 | 0.059 |
| ITT | 26.874 | 0.096 | 3.029 | 0.068 | 27.044 | 0.096 |
| TSEsimp-C | 5.220 | 0.162 | 5.119 | 0.115 | 7.309 | 0.128 |
| TSEsimp-S | 0.976 | 0.248 | 7.840 | 0.175 | 7.897 | 0.186 |
| TSEsimp-C without rec | 9.290 | 0.115 | 3.644 | 0.082 | 9.978 | 0.111 |
| TSEsimp-S without rec | 8.005 | 0.114 | 3.589 | 0.080 | 8.772 | 0.108 |
| TSEgest-C | -6.131 | 0.266 | 8.393 | 0.188 | 10.372 | 0.218 |
| TSEgest-S | -4.033 | 0.330 | 10.363 | 0.233 | 11.074 | 0.261 |
| TSEgest-C without rec | 2.739 | 0.115 | 3.628 | 0.081 | 4.552 | 0.107 |
| TSEgest-S without rec | 21.369 | 0.101 | 3.165 | 0.071 | 21.637 | 0.101 |
| IPCW-C unstabilised | 10.238 | 0.485 | 15.148 | 0.343 | 18.316 | 0.431 |
| IPCW-C stabilised | 6.362 | 0.309 | 9.636 | 0.218 | 11.542 | 0.316 |
| IPCW-S unstabilised | 10.267 | 0.651 | 15.000 | 0.461 | 18.826 | 0.597 |
| IPCW-S stabilised | 6.569 | 0.412 | 9.500 | 0.292 | 11.992 | 0.446 |

## Appendix 3: Generation of Simulated Data

The data generation procedure builds upon steps 1-6 for complex scenarios described by Latimer et al (2020), online appendices, Box A2. An additional step is required to distinguish between patients that switch onto treatment 1 and those that switch to treatment 2 (or treatments 1-5). Simulations were performed in Stata 17.

Step 1: Underlying overall survival (OS) times

Underlying OS times were derived using Crowther and Lambert’s (2012) general simulation framework using the survsim command in Stata,[19] with a 2-component mixture Weibull baseline survival function. All scenarios had lambda scale and gamma shape parameters of lambda1=0.000008 gamma1=1.96 and lambda2=0.000015 gamma2=1.71 Survsim simulated overall survival times for control and experimental group with a log hazard ratio treatment effect, and a binary variable indicating good or bad prognosis at baseline (bprog=1 for patients with bad prognosis at baseline). Time was divided into periods of 21-days, from randomisation to death.

Step 2: Progression-free survival times

Disease progression times were generated by multiplying the underlying OS times with a value from a beta distribution with shape parameters 5 and 10.

Step 3: Time-dependent confounding variable

The time dependent variable in this study is a binary indicator of metastatic disease, which can occur at any of the 4 visits following disease progression (i.e. 21, 42, 63 or 84 days post-progression). The probability of metastatic disease depends on the treatment received, A, and baseline prognosis, (p=0.24 control treatment and good prognosis, p=0.4 control group and bad prognosis, p=0.144 experimental group and good prognosis, p=0.24 experimental group and bad prognosis). A binary time dependent variable was generated for each possible time-point based on binomial random number and the probability of metastatic disease. If M occurs, the remaining survival time is reduced according to parameter *catmult*, which was set to 0.4 in our scenarios.

Step 4: Switching mechanism

1. (a) Switching is permitted in the control group at the time of disease progression and the 5 subsequent visits after disease progression. The probability of switch $Ps$ is a function of baseline prognosis, V, metastatic event at the patient’s previous visit, M, and parameters Psg, Psb and Psm.

$$Ps_{t}=f(V, M_{t-1}, Psg, Psb, Psm)$$

Where Psg influences the probability of switch for patients with baseline good prognosis, Psb influences the probability of switch for patients with bad prognosis and Psm influences the probability of switch for patients with previous metastatic event.

(b) For each patient i at time t, a binary switch indicator was assigned using a binomial random draw with the probability of success (i.e. switch equals 1) of $Ps_{t}$.

$$St=binomial (1, Ps_{t})$$

Either (ii) Two-treatment scenarios:

Switchers were assigned to a treatment using a binomial random draw based on a parameter representing the probability of switching to treatment 1 for patients with bad prognosis, Ps1b, and another binomial random draw based on a parameter probability of switching to treatment 1 for patients with good prognosis, Ps1g.

$S1t=binomial \left( 1, Ps1b \right)$ if V=1 (bad prognosis)

$S1t=binomial \left( 1, Ps1g \right)$ if V=0 (good prognosis)

$$S2t=1 if S=1,S1t=0$$

Switchers to treatment 2 were identified as the switchers that did not switch to treatment 1.

Or (ii) Five-treatment scenarios:

Switchers were assigned to treatments 1-5, based on parameters Ps1b-Ps4b, Ps1g-Ps4g and baseline prognosis.

$S1t=binomial \left( 1, Ps1b \right)$ if V=1 (bad prognosis)

$S2t=binomial \left( 1, Ps2b \right)$ if V=1 (bad prognosis), $S1t\neq1$

$S3t=binomial \left( 1, Ps3b \right)$ if V=1 (bad prognosis), $S1t\neq1, S2t\neq1$

$S4t=binomial \left( 1, Ps4b \right)$ if V=1 (bad prognosis) $S1t\neq1, S2t\neq1,S3t\neq1$

$S1t=binomial \left( 1, Ps1g \right)$ if V=0 (good prognosis)

$S2t=binomial \left( 1, Ps2g \right)$ if V=0 (good prognosis) $S1t\neq1$

$S3t=binomial \left( 1, Ps3g \right)$ if V=0 (good prognosis) $S1t\neq1, S2t\neq1$

$S4t=binomial \left( 1, Ps4g \right)$ if V=0 (good prognosis) $S1t\neq1, S2t\neq1,S3t\neq1$

$$S5t=1 if St=1,S1t=0,S2t=0,S3t=0,S4t=0$$

Note that Ps1b -Ps4b and Ps1g-Ps4g parameters were not set equal to each other, to ensure that the different treatment groups varied in their composition, in terms of proportions of good and bad prognosis patients.

Step 5: Effect of switching

For patients that switched and had not incurred M prior to or at time of switch, the occurrence on M was recalculated on the premise that the probability of M occurring was lower after treatment had been switched. For these patients the binomial random draw was repeated to update the binary indicator of metastatic event, M. If M occurs, survival time is reduced according to parameter *catmult*. If M no longer occurs (i.e. had occurred before the effect of switching was incorporated, but no longer occurred when the random draw was repeated with lower probability of M), the potential for M to occur at additional visits, that had not occurred with the previous M, must be considered. If M occurs during the additional visits, remaining survival times are reduced according to catmult.

Survival times are extended by a treatment effect time ratio, which differs depending on treatment received (*xomult1* and *xomult2* for treatments 1 and 2, respectively). Where additional visits are created, the probability of M at the additional visit is assigned based on prognosis and treatment received, and binomial random draws are used to determine if M occurs. Again, remaining survival times are reduced according to catmult if M occurs.

Step 6: Apply censoring

A study end date was set at 720 days for scenarios 1-16, which resulted in 20% censoring, 500 days for scenarios 17-32, which achieved 40% censoring and 300 days for scenarios 33-40 to achieve 70% censoring. Any switching, progression or death times that occurred after the study end date were censored at this imposed administrative censoring time.

**Appendix 4: Examples of a typical underlying K-M curve and K-M curve with switching**

Figure A4.1: Underlying K-M curve for scenario 1

Figure A4.2: K-M curve with switching for scenario 1

## Appendix 5: Simulation Study Scenario Parameter Values

Table A5.1: Scenario description and parameters (scenarios 1-40)

| scenario | N | switch  proportion | T1:T2  ratio | Treatment  effects | Censoring  proportion | Admin.  Censor days | Psb | Psg | Ps1b | Ps1g | xomult1 | xomult2 | Psm |
| --- | --- | --- | --- | --- | --- | --- | --- | --- | --- | --- | --- | --- | --- |
| 1 | 500 | 20% | 80:20 | T1 1.5; T2 1.2 | 20% | 730 | 0.12 | 0.01 | 0.86 | 0.52 | 2.71 | 1.87 | 1.23 |
| 2 | 500 | 20% | 80:20 | T1 1.2; T2 1.7 | 20% | 730 | 0.12 | 0.01 | 0.86 | 0.52 | 2.15 | 2.71 | 1.23 |
| 3 | 500 | 20% | 60:40 | T1 1.5; T2 1.2 | 20% | 730 | 0.12 | 0.01 | 0.66 | 0.35 | 2.74 | 1.96 | 1.23 |
| 4 | 500 | 20% | 60:40 | T1 1.2; T2 1.7 | 20% | 730 | 0.12 | 0.01 | 0.66 | 0.35 | 2.16 | 2.81 | 1.23 |
| 5 | 500 | 50% | 80:20 | T1 1.5; T2 1.2 | 20% | 730 | 0.6 | 0.05 | 0.92 | 0.58 | 2.72 | 1.76 | 1.23 |
| 6 | 500 | 50% | 80:20 | T1 1.2; T2 1.7 | 20% | 730 | 0.6 | 0.05 | 0.92 | 0.58 | 2.16 | 2.61 | 1.23 |
| 7 | 500 | 50% | 60:40 | T1 1.5; T2 1.2 | 20% | 730 | 0.6 | 0.05 | 0.7 | 0.44 | 2.71 | 1.95 | 1.23 |
| 8 | 500 | 50% | 60:40 | T1 1.2; T2 1.7 | 20% | 730 | 0.6 | 0.05 | 0.7 | 0.44 | 2.15 | 2.83 | 1.23 |
| 9 | 1000 | 20% | 80:20 | T1 1.5; T2 1.2 | 20% | 730 | 0.12 | 0.01 | 0.86 | 0.52 | 2.71 | 1.87 | 1.23 |
| 10 | 1000 | 20% | 80:20 | T1 1.2; T2 1.7 | 20% | 730 | 0.12 | 0.01 | 0.86 | 0.52 | 2.15 | 2.71 | 1.23 |
| 11 | 1000 | 20% | 60:40 | T1 1.5; T2 1.2 | 20% | 730 | 0.12 | 0.01 | 0.66 | 0.35 | 2.74 | 1.96 | 1.23 |
| 12 | 1000 | 20% | 60:40 | T1 1.2; T2 1.7 | 20% | 730 | 0.12 | 0.01 | 0.66 | 0.35 | 2.16 | 2.81 | 1.23 |
| 13 | 1000 | 50% | 80:20 | T1 1.5; T2 1.2 | 20% | 730 | 0.6 | 0.05 | 0.92 | 0.58 | 2.72 | 1.76 | 1.23 |
| 14 | 1000 | 50% | 80:20 | T1 1.2; T2 1.7 | 20% | 730 | 0.6 | 0.05 | 0.92 | 0.58 | 2.16 | 2.61 | 1.23 |
| 15 | 1000 | 50% | 60:40 | T1 1.5; T2 1.2 | 20% | 730 | 0.6 | 0.05 | 0.7 | 0.44 | 2.71 | 1.95 | 1.23 |
| 16 | 1000 | 50% | 60:40 | T1 1.2; T2 1.7 | 20% | 730 | 0.6 | 0.05 | 0.7 | 0.44 | 2.15 | 2.83 | 1.23 |
| 17 | 500 | 20% | 80:20 | T1 1.5; T2 1.2 | 40% | 500 | 0.12 | 0.01 | 0.86 | 0.52 | 2.71 | 1.87 | 1.23 |
| 18 | 500 | 20% | 80:20 | T1 1.2; T2 1.7 | 40% | 500 | 0.12 | 0.01 | 0.86 | 0.52 | 2.15 | 2.71 | 1.23 |
| 19 | 500 | 20% | 60:40 | T1 1.5; T2 1.2 | 40% | 500 | 0.12 | 0.01 | 0.66 | 0.35 | 2.74 | 1.96 | 1.23 |
| 20 | 500 | 20% | 60:40 | T1 1.2; T2 1.7 | 40% | 500 | 0.12 | 0.01 | 0.66 | 0.35 | 2.16 | 2.81 | 1.23 |
| 21 | 500 | 50% | 80:20 | T1 1.5; T2 1.2 | 40% | 500 | 0.6 | 0.05 | 0.92 | 0.58 | 2.72 | 1.76 | 1.23 |
| 22 | 500 | 50% | 80:20 | T1 1.2; T2 1.7 | 40% | 500 | 0.6 | 0.05 | 0.92 | 0.58 | 2.16 | 2.61 | 1.23 |
| 23 | 500 | 50% | 60:40 | T1 1.5; T2 1.2 | 40% | 500 | 0.6 | 0.05 | 0.7 | 0.44 | 2.71 | 1.95 | 1.23 |
| 24 | 500 | 50% | 60:40 | T1 1.2; T2 1.7 | 40% | 500 | 0.6 | 0.05 | 0.7 | 0.44 | 2.15 | 2.83 | 1.23 |
| 25 | 1000 | 20% | 80:20 | T1 1.5; T2 1.2 | 40% | 500 | 0.12 | 0.01 | 0.86 | 0.52 | 2.71 | 1.87 | 1.23 |
| 26 | 1000 | 20% | 80:20 | T1 1.2; T2 1.7 | 40% | 500 | 0.12 | 0.01 | 0.86 | 0.52 | 2.15 | 2.71 | 1.23 |
| 27 | 1000 | 20% | 60:40 | T1 1.5; T2 1.2 | 40% | 500 | 0.12 | 0.01 | 0.66 | 0.35 | 2.74 | 1.96 | 1.23 |
| 28 | 1000 | 20% | 60:40 | T1 1.2; T2 1.7 | 40% | 500 | 0.12 | 0.01 | 0.66 | 0.35 | 2.16 | 2.81 | 1.23 |
| 29 | 1000 | 50% | 80:20 | T1 1.5; T2 1.2 | 40% | 500 | 0.6 | 0.05 | 0.92 | 0.58 | 2.72 | 1.76 | 1.23 |
| 30 | 1000 | 50% | 80:20 | T1 1.2; T2 1.7 | 40% | 500 | 0.6 | 0.05 | 0.92 | 0.58 | 2.16 | 2.61 | 1.23 |
| 31 | 1000 | 50% | 60:40 | T1 1.5; T2 1.2 | 40% | 500 | 0.6 | 0.05 | 0.7 | 0.44 | 2.71 | 1.95 | 1.23 |
| 32 | 1000 | 50% | 60:40 | T1 1.2; T2 1.7 | 40% | 500 | 0.6 | 0.05 | 0.7 | 0.44 | 2.15 | 2.83 | 1.23 |
| 33 | 1000 | 20% | 80:20 | T1 1.5; T2 1.2 | 70% | 300 | 0.12 | 0.01 | 0.86 | 0.52 | 2.71 | 1.87 | 1.23 |
| 34 | 1000 | 20% | 80:20 | T1 1.2; T2 1.7 | 70% | 300 | 0.12 | 0.01 | 0.86 | 0.52 | 2.15 | 2.71 | 1.23 |
| 35 | 1000 | 20% | 60:40 | T1 1.5; T2 1.2 | 70% | 300 | 0.12 | 0.01 | 0.66 | 0.35 | 2.74 | 1.96 | 1.23 |
| 36 | 1000 | 20% | 60:40 | T1 1.2; T2 1.7 | 70% | 300 | 0.12 | 0.01 | 0.66 | 0.35 | 2.16 | 2.81 | 1.23 |
| 37 | 1000 | 50% | 80:20 | T1 1.5; T2 1.2 | 70% | 300 | 0.6 | 0.05 | 0.92 | 0.58 | 2.72 | 1.76 | 1.23 |
| 38 | 1000 | 50% | 80:20 | T1 1.2; T2 1.7 | 70% | 300 | 0.6 | 0.05 | 0.92 | 0.58 | 2.16 | 2.61 | 1.23 |
| 39 | 1000 | 50% | 60:40 | T1 1.5; T2 1.2 | 70% | 300 | 0.6 | 0.05 | 0.7 | 0.44 | 2.71 | 1.95 | 1.23 |
| 40 | 1000 | 50% | 60:40 | T1 1.2; T2 1.7 | 70% | 300 | 0.6 | 0.05 | 0.7 | 0.44 | 2.15 | 2.83 | 1.23 |

N represents the number of patients in each iteration of the RCT, T1:T2 ratio represent the ratio of switchers that switch to treatment 1 to switchers that switch to treatment 2, Treatment effects represent the time ratio treatment effects for treatment 1 (T1) and Treatment 2 (T2), censoring proportion represents the proportion of patients that are administratively censored, admin censor days represents the number of days after follow-up that patients are administratively censored, Psb, Psg, Ps1b, Ps1g, xomult1, xomult2, Psm are parameters in the DGM as described in Appendix A. Psb represents the probability of switching with bad prognosis, Psg represents probability of switching with good prognosis, Ps1b represents probability of switching to treatment 1 with bad prognosis, Ps1g represents the probability of switching to treatment 1 with good prognosis, xomult1 represents the treatment effect time ratio associated with receiving treatment 1, and xomult2 represents the treatment effect time ratio associated with receiving treatment 2. DGM parameters not listed in the Table remain the same across all scenarios (*lambda1=0.000008, gamma1=1.96, pmix=0.5, lambda=20.000015, gamma=21.71, trtlghr=-0.25, bprog=0.35, pcatnotrtbprog=0.4, pcattrtbprog=0.24, pcattrtbprog1=0.24, pcattrtbprog2=0.2, pcatnotrt=0.24, pcattrt=0.144, pcattrt1=0.144, pcattrt2=0.12, catmult=0.4*).

Table A5.2: Scenario description and parameters (scenarios 41-44)

| scenario | N | switch  proportion | T1:T2:T3:T4:T5  ratio | Treatment  effects | Censoring  proportion | Admin.  Censor days | Psb | Psg | Ps1b | Ps1g | xomult1 | xomult2 | xomult3 | xomult4 | xomult5 |
| --- | --- | --- | --- | --- | --- | --- | --- | --- | --- | --- | --- | --- | --- | --- | --- |
| 41 | 500 | 20% | 10:15:20:25:30 | T1 1.2, T2 1.3, T3 1.4, T4 1.6, T5 1.8 | 20% | 730 | 0.12 | 0.01 | 0.1 | 0.3 | 2 | 2.3 | 2.6 | 2.9 | 3.2 |
| 42 | 500 | 50% | 10:15:20:25:30 | T1 1.2, T2 1.3, T3 1.4, T4 1.6, T5 1.8 | 20% | 730 | 0.6 | 0.05 | 0.1 | 0.3 | 2 | 2.3 | 2.6 | 2.9 | 3.2 |
| 43 | 1000 | 20% | 10:15:20:25:30 | T1 1.2, T2 1.3, T3 1.4, T4 1.6, T5 1.8 | 20% | 730 | 0.12 | 0.01 | 0.1 | 0.3 | 2 | 2.3 | 2.6 | 2.9 | 3.2 |
| 44 | 1000 | 50% | 10:15:20:25:30 | T1 1.2, T2 1.3, T3 1.4, T4 1.6, T5 1.8 | 20% | 730 | 0.6 | 0.05 | 0.1 | 0.3 | 2 | 2.3 | 2.6 | 2.9 | 3.2 |

DGM parameters not listed in the Table remain the same across all scenarios as described in the footnote in Appendix B: Table B1 and additional parameters included in the 5-treatment DGM are listed as follows: pcattrtbprog3=0.24, pcattrtbprog4=0.2, pcattrtbprog5=0.2, pcattrt3=0.144, pcattrt4=0.12, pcattrt5=0.12

## Appendix 6: Method convergence

Table A6.1: Percentage convergence in 1000 iterations by scenario and application of method

| Scenario | No switch | ITT | TSEsimp-C | TSEsimp-S | TSEsimp-C  without rec | TSEsimp-S  without rec | TSEgest-C | TSEgest-S | TSEgest-C without rec | TSEgest-S without rec | IPCW-C unstabilised | IPCW-C  stabilised | IPCW-S  unstabilised | IPCW-S  stabilised |
| --- | --- | --- | --- | --- | --- | --- | --- | --- | --- | --- | --- | --- | --- | --- |
| 1 | 100.0% | 100.0% | 100.0% | 100.0% | 100.0% | 100.0% | 100.0% | 96.3% | 100.0% | 96.3% | 100.0% | 100.0% | 97.4% | 97.4% |
| 2 | 100.0% | 100.0% | 100.0% | 100.0% | 100.0% | 100.0% | 100.0% | 94.9% | 100.0% | 94.9% | 100.0% | 100.0% | 98.6% | 98.6% |
| 3 | 100.0% | 100.0% | 100.0% | 100.0% | 100.0% | 100.0% | 99.9% | 99.6% | 99.9% | 99.6% | 100.0% | 100.0% | 99.9% | 99.9% |
| 4 | 100.0% | 100.0% | 100.0% | 100.0% | 100.0% | 100.0% | 100.0% | 99.7% | 100.0% | 99.7% | 100.0% | 100.0% | 100.0% | 100.0% |
| 5 | 100.0% | 100.0% | 100.0% | 100.0% | 100.0% | 100.0% | 99.8% | 99.9% | 99.8% | 99.9% | 96.7% | 96.7% | 71.4% | 71.4% |
| 6 | 100.0% | 100.0% | 100.0% | 100.0% | 100.0% | 100.0% | 99.8% | 99.8% | 99.8% | 99.8% | 97.6% | 97.6% | 70.9% | 70.9% |
| 7 | 100.0% | 100.0% | 100.0% | 100.0% | 100.0% | 100.0% | 99.9% | 99.5% | 99.9% | 99.5% | 97.3% | 97.3% | 81.8% | 81.8% |
| 8 | 100.0% | 100.0% | 100.0% | 100.0% | 100.0% | 100.0% | 99.8% | 99.0% | 99.8% | 99.0% | 97.0% | 97.0% | 82.5% | 82.4% |
| 9 | 100.0% | 100.0% | 100.0% | 100.0% | 100.0% | 100.0% | 100.0% | 99.7% | 100.0% | 99.7% | 100.0% | 100.0% | 100.0% | 100.0% |
| 10 | 100.0% | 100.0% | 100.0% | 100.0% | 100.0% | 100.0% | 100.0% | 99.8% | 100.0% | 99.8% | 100.0% | 100.0% | 100.0% | 100.0% |
| 11 | 100.0% | 100.0% | 100.0% | 100.0% | 100.0% | 100.0% | 100.0% | 100.0% | 100.0% | 100.0% | 100.0% | 100.0% | 100.0% | 100.0% |
| 12 | 100.0% | 100.0% | 100.0% | 100.0% | 100.0% | 100.0% | 100.0% | 100.0% | 100.0% | 100.0% | 100.0% | 100.0% | 100.0% | 100.0% |
| 13 | 100.0% | 100.0% | 100.0% | 100.0% | 100.0% | 100.0% | 99.4% | 99.9% | 99.4% | 99.9% | 97.4% | 97.4% | 77.0% | 77.0% |
| 14 | 100.0% | 100.0% | 100.0% | 100.0% | 100.0% | 100.0% | 99.0% | 99.4% | 99.0% | 99.4% | 97.8% | 97.8% | 77.3% | 77.3% |
| 15 | 100.0% | 100.0% | 100.0% | 100.0% | 100.0% | 100.0% | 99.3% | 100.0% | 99.3% | 100.0% | 96.7% | 96.7% | 97.5% | 97.5% |
| 16 | 100.0% | 100.0% | 100.0% | 100.0% | 100.0% | 100.0% | 99.4% | 99.7% | 99.4% | 99.7% | 98.3% | 98.3% | 95.8% | 95.8% |
| 17 | 100.0% | 100.0% | 100.0% | 100.0% | 100.0% | 100.0% | 100.0% | 88.9% | 100.0% | 89.0% | 100.0% | 100.0% | 97.2% | 97.2% |
| 18 | 100.0% | 100.0% | 100.0% | 99.8% | 100.0% | 100.0% | 100.0% | 81.7% | 100.0% | 82.0% | 100.0% | 100.0% | 97.3% | 97.3% |
| 19 | 100.0% | 100.0% | 100.0% | 100.0% | 100.0% | 100.0% | 99.8% | 97.4% | 99.8% | 97.5% | 100.0% | 100.0% | 100.0% | 100.0% |
| 20 | 100.0% | 100.0% | 100.0% | 100.0% | 100.0% | 100.0% | 99.9% | 94.3% | 99.9% | 94.4% | 100.0% | 100.0% | 100.0% | 100.0% |
| 21 | 100.0% | 100.0% | 100.0% | 100.0% | 100.0% | 100.0% | 99.9% | 99.2% | 99.9% | 99.2% | 97.8% | 97.8% | 70.4% | 70.4% |
| 22 | 100.0% | 100.0% | 100.0% | 100.0% | 100.0% | 100.0% | 99.7% | 98.8% | 99.7% | 98.8% | 96.7% | 96.8% | 69.3% | 69.2% |
| 23 | 100.0% | 100.0% | 100.0% | 100.0% | 100.0% | 100.0% | 99.7% | 99.7% | 99.7% | 99.7% | 95.5% | 95.5% | 83.2% | 83.2% |
| 24 | 100.0% | 100.0% | 100.0% | 100.0% | 100.0% | 100.0% | 99.9% | 99.8% | 99.9% | 99.8% | 96.0% | 96.1% | 81.9% | 81.8% |
| 25 | 100.0% | 100.0% | 100.0% | 100.0% | 100.0% | 100.0% | 100.0% | 97.7% | 100.0% | 98.1% | 100.0% | 100.0% | 99.8% | 99.8% |
| 26 | 100.0% | 100.0% | 100.0% | 100.0% | 100.0% | 100.0% | 100.0% | 94.6% | 100.0% | 95.0% | 100.0% | 100.0% | 100.0% | 100.0% |
| 27 | 100.0% | 100.0% | 100.0% | 100.0% | 100.0% | 100.0% | 100.0% | 99.9% | 100.0% | 99.9% | 100.0% | 100.0% | 100.0% | 100.0% |
| 28 | 100.0% | 100.0% | 100.0% | 100.0% | 100.0% | 100.0% | 100.0% | 99.7% | 100.0% | 99.8% | 100.0% | 100.0% | 100.0% | 100.0% |
| 29 | 100.0% | 100.0% | 100.0% | 100.0% | 100.0% | 100.0% | 99.5% | 100.0% | 99.5% | 100.0% | 97.7% | 97.7% | 79.7% | 79.7% |
| 30 | 100.0% | 100.0% | 100.0% | 100.0% | 100.0% | 100.0% | 99.6% | 100.0% | 99.6% | 100.0% | 98.1% | 98.1% | 80.9% | 80.9% |
| 31 | 100.0% | 100.0% | 100.0% | 100.0% | 100.0% | 100.0% | 99.4% | 100.0% | 99.4% | 100.0% | 97.7% | 97.7% | 95.8% | 95.8% |
| 32 | 100.0% | 100.0% | 100.0% | 100.0% | 100.0% | 100.0% | 99.3% | 100.0% | 99.3% | 100.0% | 97.5% | 97.5% | 94.8% | 94.8% |
| 33 | 100.0% | 100.0% | 100.0% | 100.0% | 100.0% | 100.0% | 99.7% | 81.1% | 99.7% | 81.6% | 100.0% | 100.0% | 99.7% | 99.7% |
| 34 | 100.0% | 100.0% | 100.0% | 100.0% | 100.0% | 100.0% | 99.9% | 68.2% | 99.9% | 68.6% | 100.0% | 100.0% | 100.0% | 100.0% |
| 35 | 100.0% | 100.0% | 100.0% | 100.0% | 100.0% | 100.0% | 99.8% | 93.5% | 99.8% | 93.6% | 100.0% | 100.0% | 100.0% | 100.0% |
| 36 | 100.0% | 100.0% | 100.0% | 100.0% | 100.0% | 100.0% | 99.6% | 84.7% | 99.6% | 84.9% | 100.0% | 100.0% | 100.0% | 100.0% |
| 37 | 100.0% | 100.0% | 100.0% | 100.0% | 100.0% | 100.0% | 99.6% | 95.9% | 99.6% | 95.9% | 98.1% | 98.1% | 79.3% | 79.3% |
| 38 | 100.0% | 100.0% | 100.0% | 100.0% | 100.0% | 100.0% | 99.7% | 92.0% | 99.7% | 92.1% | 97.0% | 97.0% | 76.0% | 76.0% |
| 39 | 100.0% | 100.0% | 100.0% | 100.0% | 100.0% | 100.0% | 99.6% | 99.7% | 99.6% | 99.7% | 97.2% | 97.2% | 95.5% | 95.5% |
| 40 | 100.0% | 100.0% | 100.0% | 100.0% | 100.0% | 100.0% | 99.5% | 100.0% | 99.5% | 100.0% | 97.3% | 97.3% | 95.5% | 95.5% |
| 41 | 99.8% | 99.8% | 99.8% | 99.8% | 99.8% | 99.8% | 99.8% | 93.2% | 99.8% | 93.3% | 99.8% | 99.8% | 79.4% | 79.4% |
| 42 | 100.0% | 100.0% | 100.0% | 100.0% | 100.0% | 100.0% | 99.6% | 99.2% | 99.6% | 99.2% | 96.8% | 96.9% | 21.1% | 21.1% |
| 43 | 100.0% | 100.0% | 100.0% | 100.0% | 100.0% | 100.0% | 100.0% | 99.8% | 100.0% | 99.8% | 100.0% | 100.0% | 99.6% | 99.6% |
| 44 | 100.0% | 100.0% | 100.0% | 100.0% | 100.0% | 100.0% | 99.5% | 98.8% | 99.5% | 98.8% | 97.5% | 97.5% | 53.1% | 53.1% |

## Appendix 7: IPCW-S compared with IPCW-C for the iterations of the simulation when IPCW-S converged.

| scenario | Bias in RMST | | Bias MCSE | | ModSE | | ModSE MCSE | | EmpSE | | EmpSE | | RMSE | | RMSE | | Number of iterations compared |  |
| --- | --- | --- | --- | --- | --- | --- | --- | --- | --- | --- | --- | --- | --- | --- | --- | --- | --- | --- |
|  |  |  |  |  |  |  |  |  |  |  |  |  |  |  |  |  |  |  |
|  | IPCW-C | IPCW-S | IPCW-C | IPCW-S | IPCW-C | IPCW-S | IPCW-C | IPCW-S | IPCW-C | IPCW-S | IPCW-C | IPCW-S | IPCW-C | IPCW-S | IPCW-C | IPCW-S |  |  |
| 1 | 1.437 | 1.435 | 0.132 | 0.132 | 3.663 | 3.663 | 0.000 | 0.000 | 4.122 | 4.123 | 0.093 | 0.093 | 4.349 | 4.349 | 0.094 | 0.094 | 974 |  |
| 2 | 1.488 | 1.485 | 0.130 | 0.130 | 3.661 | 3.661 | 0.000 | 0.000 | 4.094 | 4.091 | 0.092 | 0.092 | 4.358 | 4.355 | 0.099 | 0.099 | 986 |  |
| 3 | 1.439 | 1.433 | 0.128 | 0.128 | 3.665 | 3.666 | 0.000 | 0.000 | 4.031 | 4.033 | 0.090 | 0.090 | 4.276 | 4.277 | 0.098 | 0.098 | 999 |  |
| 4 | 1.341 | 1.337 | 0.131 | 0.131 | 3.665 | 3.666 | 0.000 | 0.000 | 4.131 | 4.131 | 0.092 | 0.092 | 4.341 | 4.340 | 0.094 | 0.094 | 1000 |  |
| 5 | 10.324 | 10.177 | 0.484 | 0.484 | 4.324 | 4.317 | 0.000 | 0.001 | 12.937 | 12.935 | 0.343 | 0.343 | 16.909 | 17.780 | 0.470 | 0.490 | 714 |  |
| 6 | 9.994 | 10.015 | 0.500 | 0.500 | 4.329 | 4.331 | 0.000 | 0.000 | 13.309 | 13.301 | 0.354 | 0.353 | 16.705 | 17.869 | 0.469 | 0.486 | 709 |  |
| 7 | 10.608 | 10.502 | 0.473 | 0.472 | 4.298 | 4.294 | 0.000 | 0.000 | 13.518 | 13.506 | 0.334 | 0.334 | 17.228 | 17.258 | 0.460 | 0.459 | 818 |  |
| 8 | 10.322 | 10.250 | 0.479 | 0.479 | 4.288 | 4.284 | 0.000 | 0.000 | 13.745 | 13.768 | 0.339 | 0.339 | 17.463 | 17.548 | 0.457 | 0.458 | 825 |  |
| 9 | 1.486 | 1.485 | 0.090 | 0.090 | 3.082 | 3.083 | 0.000 | 0.000 | 2.836 | 2.836 | 0.063 | 0.063 | 3.201 | 3.200 | 0.070 | 0.070 | 1000 |  |
| 10 | 1.407 | 1.405 | 0.092 | 0.092 | 3.078 | 3.079 | 0.000 | 0.000 | 2.913 | 2.911 | 0.065 | 0.065 | 3.234 | 3.232 | 0.073 | 0.073 | 1000 |  |
| 11 | 1.433 | 1.430 | 0.091 | 0.091 | 3.081 | 3.081 | 0.000 | 0.000 | 2.888 | 2.889 | 0.065 | 0.065 | 3.223 | 3.222 | 0.070 | 0.070 | 1000 |  |
| 12 | 1.514 | 1.513 | 0.094 | 0.094 | 3.082 | 3.082 | 0.000 | 0.000 | 2.973 | 2.972 | 0.067 | 0.066 | 3.335 | 3.333 | 0.073 | 0.073 | 1000 |  |
| 13 | 5.965 | 5.937 | 0.336 | 0.337 | 3.579 | 3.577 | 0.000 | 0.000 | 9.327 | 9.349 | 0.238 | 0.238 | 11.466 | 12.587 | 0.370 | 0.433 | 770 |  |
| 14 | 5.939 | 5.942 | 0.338 | 0.341 | 3.580 | 3.578 | 0.000 | 0.000 | 9.400 | 9.482 | 0.239 | 0.241 | 11.541 | 12.240 | 0.365 | 0.408 | 773 |  |
| 15 | 6.349 | 6.141 | 0.306 | 0.302 | 3.601 | 3.594 | 0.000 | 0.000 | 9.556 | 9.438 | 0.217 | 0.214 | 11.446 | 11.306 | 0.320 | 0.320 | 975 |  |
| 16 | 6.637 | 6.582 | 0.313 | 0.313 | 3.602 | 3.599 | 0.000 | 0.000 | 9.699 | 9.682 | 0.222 | 0.221 | 11.812 | 11.843 | 0.348 | 0.348 | 958 |  |
| 17 | 0.873 | 0.870 | 0.106 | 0.106 | 3.180 | 3.181 | 0.000 | 0.000 | 3.290 | 3.290 | 0.075 | 0.075 | 3.390 | 3.389 | 0.077 | 0.077 | 972 |  |
| 18 | 0.738 | 0.733 | 0.106 | 0.106 | 3.181 | 3.181 | 0.000 | 0.000 | 3.298 | 3.296 | 0.075 | 0.075 | 3.378 | 3.376 | 0.072 | 0.072 | 973 |  |
| 19 | 0.859 | 0.855 | 0.106 | 0.106 | 3.179 | 3.179 | 0.000 | 0.000 | 3.354 | 3.353 | 0.075 | 0.075 | 3.461 | 3.459 | 0.076 | 0.076 | 1000 |  |
| 20 | 0.861 | 0.857 | 0.107 | 0.107 | 3.180 | 3.181 | 0.000 | 0.000 | 3.384 | 3.384 | 0.076 | 0.076 | 3.491 | 3.489 | 0.077 | 0.077 | 1000 |  |
| 21 | 6.653 | 6.680 | 0.356 | 0.356 | 3.544 | 3.546 | 0.000 | 0.000 | 9.454 | 9.442 | 0.252 | 0.252 | 11.530 | 11.990 | 0.288 | 0.298 | 704 |  |
| 22 | 5.647 | 5.642 | 0.344 | 0.345 | 3.537 | 3.536 | 0.000 | 0.000 | 9.049 | 9.073 | 0.243 | 0.244 | 10.908 | 11.337 | 0.275 | 0.279 | 693 |  |
| 23 | 5.891 | 5.734 | 0.319 | 0.320 | 3.527 | 3.523 | 0.000 | 0.000 | 9.201 | 9.226 | 0.226 | 0.226 | 11.060 | 11.098 | 0.250 | 0.252 | 832 |  |
| 24 | 5.412 | 5.387 | 0.317 | 0.317 | 3.536 | 3.534 | 0.000 | 0.000 | 9.068 | 9.064 | 0.224 | 0.224 | 10.632 | 10.758 | 0.254 | 0.258 | 819 |  |
| 25 | 0.819 | 0.818 | 0.073 | 0.073 | 2.676 | 2.676 | 0.000 | 0.000 | 2.318 | 2.319 | 0.052 | 0.052 | 2.458 | 2.458 | 0.055 | 0.055 | 998 |  |
| 26 | 0.758 | 0.756 | 0.075 | 0.075 | 2.675 | 2.675 | 0.000 | 0.000 | 2.361 | 2.360 | 0.053 | 0.053 | 2.478 | 2.477 | 0.058 | 0.058 | 1000 |  |
| 27 | 0.757 | 0.754 | 0.073 | 0.073 | 2.674 | 2.674 | 0.000 | 0.000 | 2.299 | 2.299 | 0.051 | 0.051 | 2.419 | 2.419 | 0.054 | 0.054 | 1000 |  |
| 28 | 0.755 | 0.753 | 0.073 | 0.073 | 2.675 | 2.675 | 0.000 | 0.000 | 2.313 | 2.314 | 0.052 | 0.052 | 2.432 | 2.432 | 0.053 | 0.053 | 1000 |  |
| 29 | 3.926 | 3.869 | 0.245 | 0.246 | 3.001 | 3.000 | 0.000 | 0.000 | 6.920 | 6.939 | 0.173 | 0.174 | 7.855 | 8.508 | 0.199 | 0.232 | 797 |  |
| 30 | 3.217 | 3.186 | 0.238 | 0.238 | 2.991 | 2.989 | 0.000 | 0.000 | 6.776 | 6.783 | 0.169 | 0.169 | 7.626 | 8.284 | 0.206 | 0.237 | 809 |  |
| 31 | 3.762 | 3.667 | 0.225 | 0.224 | 2.999 | 2.997 | 0.000 | 0.000 | 6.976 | 6.936 | 0.159 | 0.159 | 7.949 | 7.903 | 0.190 | 0.190 | 958 |  |
| 32 | 3.957 | 3.870 | 0.228 | 0.229 | 3.002 | 2.998 | 0.000 | 0.000 | 7.009 | 7.045 | 0.161 | 0.162 | 8.106 | 8.180 | 0.184 | 0.189 | 948 |  |
| 33 | 0.234 | 0.231 | 0.049 | 0.049 | 1.949 | 1.950 | 0.000 | 0.000 | 1.544 | 1.545 | 0.035 | 0.035 | 1.562 | 1.562 | 0.038 | 0.038 | 997 |  |
| 34 | 0.143 | 0.139 | 0.050 | 0.050 | 1.950 | 1.950 | 0.000 | 0.000 | 1.590 | 1.590 | 0.036 | 0.036 | 1.596 | 1.596 | 0.037 | 0.037 | 1000 |  |
| 35 | 0.181 | 0.177 | 0.051 | 0.051 | 1.950 | 1.950 | 0.000 | 0.000 | 1.601 | 1.602 | 0.036 | 0.036 | 1.611 | 1.611 | 0.036 | 0.036 | 1000 |  |
| 36 | 0.121 | 0.117 | 0.052 | 0.052 | 1.950 | 1.951 | 0.000 | 0.000 | 1.629 | 1.629 | 0.036 | 0.036 | 1.633 | 1.633 | 0.041 | 0.041 | 1000 |  |
| 37 | 1.148 | 1.149 | 0.125 | 0.126 | 2.137 | 2.137 | 0.000 | 0.000 | 3.532 | 3.541 | 0.089 | 0.089 | 3.738 | 4.238 | 0.087 | 0.212 | 793 |  |
| 38 | 1.039 | 1.014 | 0.137 | 0.137 | 2.138 | 2.137 | 0.000 | 0.000 | 3.766 | 3.788 | 0.097 | 0.097 | 3.929 | 4.697 | 0.096 | 0.277 | 760 |  |
| 39 | 1.251 | 1.203 | 0.124 | 0.124 | 2.131 | 2.131 | 0.000 | 0.000 | 3.829 | 3.834 | 0.088 | 0.088 | 4.030 | 4.099 | 0.083 | 0.111 | 955 |  |
| 40 | 0.960 | 0.906 | 0.123 | 0.123 | 2.133 | 2.133 | 0.001 | 0.000 | 3.804 | 3.800 | 0.087 | 0.087 | 3.915 | 3.927 | 0.082 | 0.085 | 955 |  |
| Note: IPCW-C and IPCW-S results displayed in this table used stabilised weights. | | | | | | | | | | | | | | | | | |  |

**Appendix 8: Scenario with time ratios of 1.1 for treatment 1 and 2.2 for treatment 2**

To test the sensitivity of our conclusions to a larger difference between the treatment effect of treatment 1 and treatment 2, we ran 4 additional scenarios. Results of these scenarios (45-48) are presented in figures A8.1-A8.3. The parameters of these scenarios (shown in table A8.1) reflected the sample size, switch proportions, T1:T2 ratio, censoring proportion and administrative censor days used in scenarios 1-8, and therefore scenario 45 is directly comparable to scenarios 1 and 2, scenario 46 is comparable to 3 and 4, scenario 47 to 5 and 6 and scenario 48 to 7 and 8.

Table A8.1: Scenario description and parameters for additional scenarios 45-48

| scenario | N | switch  proportion | T1:T2  ratio | Treatment  effects | Censoring  proportion | Admin.  Censor days |
| --- | --- | --- | --- | --- | --- | --- |
| 45 | 500 | 20% | 80:20 | T1 1.1; T2 2.2 | 20% | 730 |
| 46 | 500 | 20% | 60:40 | T1 1.1; T2 2.2 | 20% | 730 |
| 47 | 500 | 50% | 80:20 | T1 1.1; T2 2.2 | 20% | 730 |
| 48 | 500 | 50% | 60:40 | T1 1.1; T2 2.2 | 20% | 730 |

N represents the number of patients in each iteration of the RCT, T1:T2 ratio represent the ratio of switchers that switch to treatment 1 to switchers that switch to treatment 2, Treatment effects represent the time ratio treatment effects for treatment 1 (T1) and Treatment 2 (T2), censoring proportion represents the proportion of patients that are administratively censored, admin censor days represents the number of days after follow-up that patients are administratively censored.

**Figure A8.1: Percentage bias in control group RMST, Scenarios 45-48 (censoring approximately 20%)**

**Figure A8.2: Empirical Standard Error, scenarios 45-48 (censoring approximately 20%)**

**Figure A8.3: Root Mean Squared Error, scenarios 45-48 (censoring approximately 20%)**

## Appendix 9: IPCW using n separate binary logistic models

We also compared an alternative approach to obtain a weight from n binary logistic models, where n represents the n different types of switch in the control group. Separate probabilities of switching were derived using n binary logistic models.

Numerator:

$logit \left( p_{num}^{1}\left( {XO}^{1} \right) \right)=\beta_{0}+\beta V$ if control group=1

$logit \left( p_{num}^{2}\left( {XO}^{2} \right) \right)=\beta_{0}+\beta V$ if control group=1

…..

$logit \left( p_{num}^{n}\left( {XO}^{n} \right) \right)=\beta_{0}+\beta V$ if control group=1

Where ${XO}^{n}$ represents a binary time-dependent indicator of switch to treatment n, and V represents baseline prognosis. Following estimation of each logistic model, the probabilities of switching to treatment n (i.e. P(switch=n)) were obtained using the “predict, pr” post-estimation command in Stata 17. For each patient at each time point t, the cumulative probability of switching to treatment n was calculated. This is repeated for each treatment n, = 1,2,…N.

$$p_{num-C}^{1}=\sum_{t}^{t0} (1-p_{num}^{1})$$

$$p_{num-C}^{2}=\sum_{t}^{t0} (1-p_{num}^{2})$$

…..

$$p_{num-C}^{n}=\sum_{t}^{t0} (1-p_{num}^{n})$$

Denominator:

A set of denominator models were estimated for each type of switch n, for patients in the control group at time points where switch was possible (i.e. visit 1-6 post-progression)

$logit \left( p_{denom}^{1}\left( {XO}^{1} \right) \right)=\beta_{0}+\sum_{m} {\beta_{m}VM}_{t-1}$ if control group=1

$logit \left( p_{denom}^{2}\left( XO^{2} \right) \right)=\beta_{0}+\sum_{m} {\beta_{m}VM}_{t-1}$ if control group=1

…..

$logit \left( p_{denom}^{n}\left( {XO}^{n} \right) \right)=\beta_{0}+\sum_{m} {\beta_{m}VM}_{t-1}$ if control group=1

Again, a separate model was estimated for each treatment n=1,2,…N. Probabilities P(switch=n)) were extracted from each model, and cumulative probabilities were calculated.

$$p_{denom-C}^{1}=\sum_{t}^{t0} (1-p_{denom}^{1})$$

$$p_{denom-C}^{2}=\sum_{t}^{t0} (1-p_{denom}^{2})$$

…..

$$p_{denom-C}^{n}=\sum_{t}^{t0} (1-p_{denom}^{n})$$

Weights:

Weights were calculated as follows and set equal to 1 for treatment group patients.

$$SW=\frac{p_{num-C}^{1}}{p_{denom-C}^{1}}.\frac{p_{num-C}^{2}}{p_{denom-C}^{2}}\ldots\frac{p_{num-C}^{n}}{p_{denom-C}^{n}}$$

$$UW=\frac{1}{p_{denom-C}^{1}}.\frac{1}{p_{denom-C}^{2}}\ldots\frac{1}{p_{denom-C}^{n}}$$

Outcome model:

A logistic model with a binary indicator of survival as the dependent variable and incorporating stabilised or unstabilised weights, was applied to the time dependent data censored at time of switch. Models with stabilised weights additionally included baseline confounders as explanatory variables.

**Figure A9.1: Percentage bias in control group RMST, (censoring approximately 20%)**

**
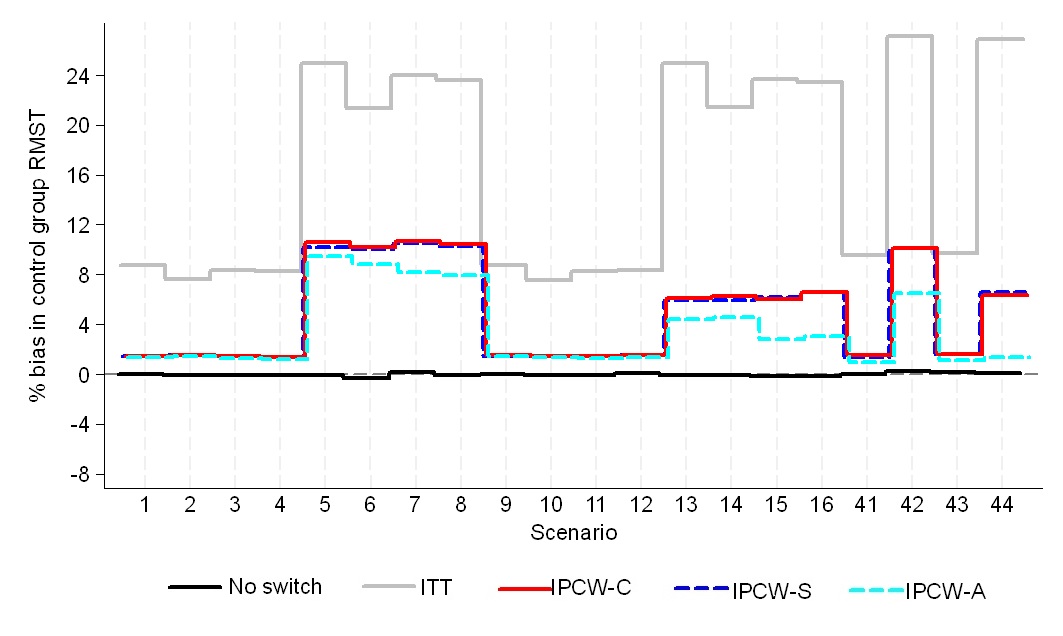
**

**Footnote:** IPCW-C represents combined strategy, IPCW-S represents separate strategy using the multinomial logit as described in the main paper and Appendix 1, and IPCW-A represents separate strategy using n binary logistic models as described in Appendix A9, with stabilised weights.

Figure A9.2: Empirical standard error


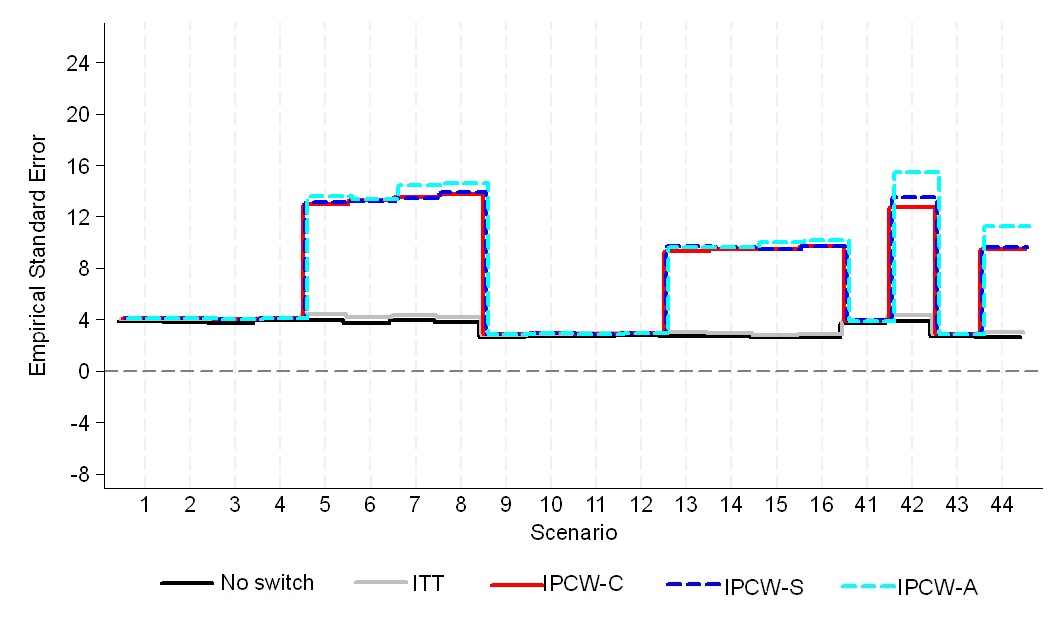


Figure A9.3: Root Squared standard error


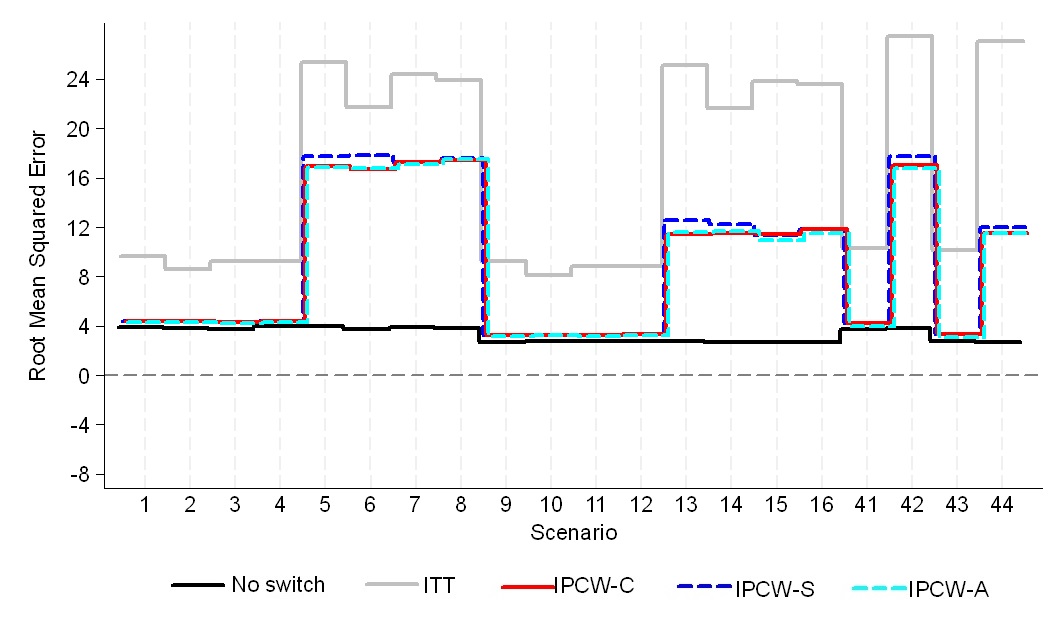


**Footnote:** IPCW-C represents combined strategy, IPCW-S represents separate strategy using the multinomial logit as described in the main paper and Appendix 1, and IPCW-A represents separate strategy using n binary logistic models as described in Appendix 9, with stabilised weights.

Figures A9.1-A9.3 indicate the IPCW using n separate binary logistic models produces a similar pattern of results to IPCW-C and IPCW-S, in terms of bias, empirical standard error and root mean squared standard error. IPCW-A produces slightly lower bias than IPCW-C and IPCW-S in scenarios with high proportions of switchers, but still producing high bias. None of the IPCW applications perform well in scenarios with high proportions of switchers.

Table A9.1 indicates the proportion of iterations that converged using each method. Although IPCW-A converged in more iterations than IPCW-S, the convergence proportions were up to 15 percentage points lower than IPCW-C.

Table A9.1: IPCW convergence proportions

| Scenario | IPCW-C | IPCW-S | IPCW-A |
| --- | --- | --- | --- |
| 1 | 100 | 97.4 | 100 |
| 2 | 100 | 98.6 | 100 |
| 3 | 100 | 99.9 | 100 |
| 4 | 100 | 100 | 100 |
| 5 | 96.7 | 71.4 | 86.6 |
| 6 | 97.6 | 70.9 | 87.1 |
| 7 | 97.3 | 81.8 | 87.4 |
| 8 | 97 | 82.5 | 85.7 |
| 9 | 100 | 100 | 100 |
| 10 | 100 | 100 | 100 |
| 11 | 100 | 100 | 100 |
| 12 | 100 | 100 | 100 |
| 13 | 97.4 | 77 | 83 |
| 14 | 97.8 | 77.3 | 82.2 |
| 15 | 96.7 | 97.5 | 83.6 |
| 16 | 98.3 | 95.8 | 82.5 |
| 17 | 99.8 | 79.4 | 95.5 |
| 18 | 96.9 | 21.1 | 99.7 |
| 19 | 100 | 99.6 | 100 |
| 20 | 97.5 | 53.1 | 99.8 |

## Appendix 10: Acceleration factors for TSEgest and TSEsimp summary statistics

Table 10.1: Acceleration factors (AF) summary statistics for scenario 1

| Variable | Mean AF | Std. dev. | Min AF | Max AF |
| --- | --- | --- | --- | --- |
| TSEgest-C AF | 2.866 | 0.556 | 1.394 | 7.338 |
| TSEgest-S AF1 | 3.040 | 0.643 | 1.411 | 6.782 |
| TSEgest-S AF1 | 2.771 | 4.661 | 0.518 | 73.111 |
| TSEsimp-C AF | 2.206 | 0.343 | 1.337 | 3.493 |
| TSEsimp-S AF1 | 2.339 | 0.392 | 1.396 | 3.808 |
| TSEsimp-S AF2 | 1.795 | 0.594 | 0.560 | 5.772 |
| Difference between TSEgest-C AF and TSEsimp-C AF | 0.660 | 0.331 | -0.069 | 3.845 |
| Difference between TSEgest-S AF1 and TSEsimp-S AF1 | 0.701 | 0.398 | -0.116 | 3.071 |
| Difference between TSEgest-S AF2 and TSEsimp-S AF2 | 0.981 | 4.521 | -0.445 | 71.243 |
